# A pattern recognition artificial olfactory system based on human olfactory receptors and organic synaptic devices

**DOI:** 10.1126/sciadv.adl2882

**Published:** 2024-05-23

**Authors:** Hyun Woo Song, Dongseok Moon, Yousang Won, Yeon Kyung Cha, Jin Yoo, Tai Hyun Park, Joon Hak Oh

**Affiliations:** ^1^ School of Chemical and Biological Engineering, Institute of Chemical Processes, Seoul National University, Seoul 08826, Republic of Korea.; ^2^ Interdisciplinary Program in Bioengineering, Seoul National University, Seoul 08826, Republic of Korea.; ^3^ Korea Institute of Science and Technology (KIST), Seoul 02792, Republic of Korea.; ^4^ Department of Nutritional Science and Food Management, Ewha Womans University, Seoul 03760, Republic of Korea.

## Abstract

Neuromorphic sensors, designed to emulate natural sensory systems, hold the promise of revolutionizing data extraction by facilitating rapid and energy-efficient analysis of extensive datasets. However, a challenge lies in accurately distinguishing specific analytes within mixtures of chemically similar compounds using existing neuromorphic chemical sensors. In this study, we present an artificial olfactory system (AOS), developed through the integration of human olfactory receptors (hORs) and artificial synapses. This AOS is engineered by interfacing an hOR-functionalized extended gate with an organic synaptic device. The AOS generates distinct patterns for odorants and mixtures thereof, at the molecular chain length level, attributed to specific hOR-odorant binding affinities. This approach enables precise pattern recognition via training and inference simulations. These findings establish a foundation for the development of high-performance sensor platforms and artificial sensory systems, which are ideal for applications in wearable and implantable devices.

## Teaser

Convergence of human olfactory receptors and synaptic devices enables odorant discrimination at the molecular chain length level.

## INTRODUCTION

The exponential growth in the realm of internet of things (IoT) technologies has led to increasing demand for state-of-the-art electronic sensors that can rapidly process and store intricate data while extracting vital information (*1*). To address this demand, there have been notable advancements in the field of neuromorphic sensors (*2*–*4*), inspired by the complexity and efficiency of biological sensory systems. The biological sensory system seamlessly integrates numerous functionalities, including sensing, processing, storage, and inference, thereby achieving exceptional accuracy in terms of perceiving stimuli and delivering appropriate responses to motor divisions (*5*). Despite considerable progress in neuromorphic sensors, their performance remains suboptimal compared with the performance of biological sensory systems. For example, the discrimination capabilities of neuromorphic olfactory sensors are not comparable with mammalian olfactory systems, which can identify an extensive range of odorants and corresponding mixtures. Consequently, existing neuromorphic olfactory sensors are primarily focused on the detection of single molecules or compounds with easily distinguishable compositions (*6*–*18*). To usher in the era of next-generation electronics, there is a need to develop neuromorphic olfactory sensors that can discern between molecules with subtle differences such as chain length or functional groups (*19*). There are several sensing platforms detecting or analyzing odorants in gas phase, such as, gas chromatography–mass spectrometry (GC-MS), surface-enhanced Raman spectroscopy, conventional transistor (*19*–*24*), resistor (*25*, *26*), electromechanical gadget (*27*, *28*), and plasmonic resonance gadget (*29*–*36*). Although GC-MS is widely used for volatile organic compounds analysis, it cannot mimic the human olfactory system because natural human olfaction recognizes the odorants in the environment as a pattern of olfactory receptor signals, but GC/MS detects odorants as chemical molecule. Other types of sensors also have some limitations to be used to future IoT applications, including difficulty in miniaturization and high energy consumption in deep learning due to the von Neumann bottleneck.

**Fig. 1.**
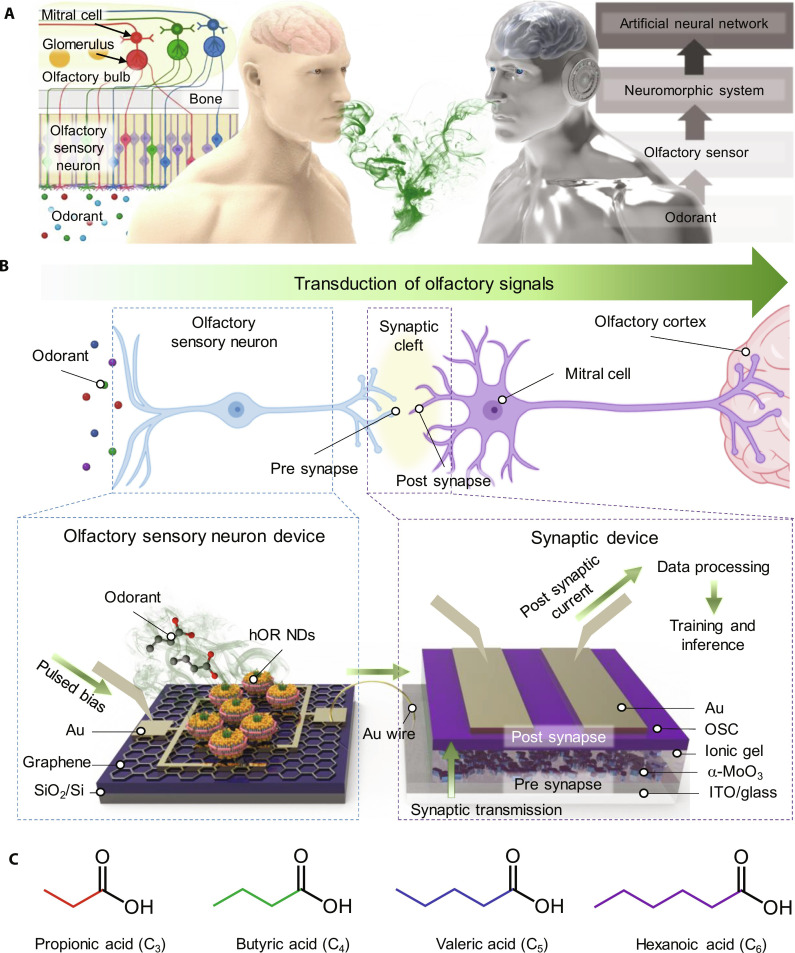
Schematic overview. (**A**) Illustration of the human olfactory system (left) and AOS (right). (**B**) Correlation between the human olfactory system and AOS and structural overview of the AOS. (**C**) Molecular structures of SCFAs.

In this study, we introduce an artificial olfactory system (AOS) that emulates the sophisticated human olfactory system through the synergistic integration of human olfactory receptors (hORs) with artificial synaptic devices. This AOS is capable of identifying odorants with remarkable precision at the chain length level, through specific binding interactions between hORs and odorants, followed by training focused on feature patterns within these odorants. As shown in Fig. 1A, the human olfactory system operates through the binding of odorants in the nasal cavity to olfactory receptors present on the surface of olfactory sensory neurons in the main olfactory epithelium. This binding triggers olfactory sensory neurons depolarization, leading to the transmission of odorant information to the glomerulus in the olfactory bulb, where distinct patterns are formed (*37*, *38*). Furthermore, mitral cells in the olfactory bulb refine and optimize these patterns to improve odor recognition and transmit the processed patterns to the olfactory cortex (*39*–*41*). Inspired by the complexity of the human olfactory system, our AOS comprises three integral components: (i) an hOR-functionalized olfactory sensor for detecting target odorants, (ii) an artificial synapse that emulates the neuroplasticity of biological synapses, forming a neuromorphic system (*42*), and (iii) an artificial neural network (ANN) proficient in learning and identifying distinct odor patterns with high accuracy. The similarities of human olfactory system and AOS components are illustrated in Fig. 1B.

In our approach, we used hORs as biomaterials to detect target odorants. This approach was achieved through an effective refolding process that transformed hORs purified from *Escherichia coli* into stable structures. To accomplish functional reconstitution of membrane proteins with ligand-binding capabilities, we conducted nanodisc (ND) assembly, a technique gaining prominence in various applications including structural analysis of membrane proteins, binding affinity analysis, and biosensors, because of its exceptional stability and prolonged functionality (*43*–*48*). By using membrane scaffold proteins (MSPs) in ND assembly, we ensured the stable incorporation of properly folded hORs within the lipid bilayer. Specifically, three hORs—hOR51E1, hOR51E2, and hOR52D1—were successfully reconstituted into NDs and functionalized on a graphene extended-gate device for combinatorial detection of various odorants.

Upon odorant exposure, the olfactory sensory neuron device, which includes hOR NDs on a graphene device, undergoes intricate conformational interactions, leading to substantial OR modification. These molecular interactions result in a detectable change in olfactory sensory neuron device resistance, which acts as a transducing event. The transduced signal is then transmitted along a gold wire to the presynaptic electrode, where ions in the ionic gel are suitably stimulated, inducing a modulated channel conductance change in the postsynaptic layer. The channel conductance patterns displayed by the postsynaptic layer are strongly dependent on the nature of the encountered odorant, rendering a unique representation of the odorant’s properties. Ultimately, with the assistance of the ANN, the postsynaptic conductance patterns are interpreted into pattern learning and recognition processes that mirror analogous signal transmission pathways present in the human olfactory system, where sensory neurons convey signals to the olfactory cortex through the olfactory signal transduction pathway (*49*).

Using the hOR-embedded AOS, we successfully identified short-chain fatty acids (SCFAs), including propionic acid (PA), butyric acid (BA), valeric acid (VA), and hexanoic acid (HA) (Fig. 1C). These SCFAs are notable either individually or in combination as biomarkers or microbial environmental indicators at the molecular chain length level (*50*–*52*). Notably, in training and inference simulations, the AOS exhibited greater than 90% accuracy in identifying single SCFAs and their mixtures.

## RESULTS

### Characteristics of hOR-embedded NDs for detecting SCFAs

**Fig. 2.**
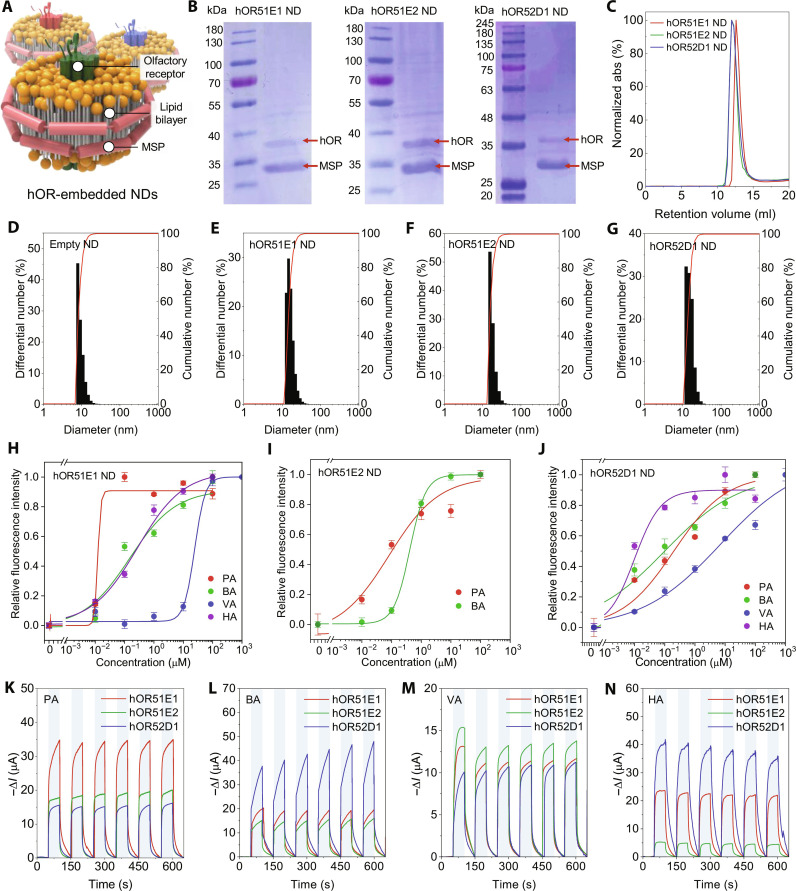
Characteristics of hOR NDs. (**A**) Illustration of NDs embedded with hORs. (**B**) Coomassie blue staining analysis of hOR51E1, hOR51E2, and hOR52D1 NDs. (**C**) Size exclusion chromatography profiles of hOR51E1, hOR51E2, and hOR52D1 NDs. (**D** to **G**) Dynamic light scattering analysis profiles for (D) empty, (E) hOR51E1, (F) hOR51E2, and (G) hOR52D1 NDs. (**H** to **J**) Tryptophan fluorescence quenching assay of various binding affinities between fatty acids and (H) hOR51E1, (I) hOR51E2, and (J) hOR52D1 NDs. Error bars represent SEM (*n* = 5). (**K** to **N**) Responses of graphene extended-gates in 3 ppm of (A) PA, (B) BA, (C) VA, and (D) HA. Operation voltage is −0.1 V and gas exposing duration is 50 s.

The three hORs were overexpressed in an *E. coli* strain coexpressing RraA, termed SuptoxR, and were purified using the same procedure as detailed in a previous study (*43*). Coomassie blue staining and Western blotting analysis confirmed the purity and expression of the hORs and MSP. The purified hORs and MSP were used to assemble NDs. The MSPs firmly secure the lipid bilayer, preserving the native membrane environment; the properly folded hOR is integrated into this lipid membrane (Fig. 2A). The presence of hOR and MSP as components of the ND was confirmed by SDS–polyacrylamide gel electrophoresis analysis (Fig. 2B), in which the three hORs appeared at approximately 38 kDa and the MSP appeared at approximately 33 kDa. The ultraviolet (UV) absorbance peak for the three hOR NDs was observed at a retention volume of approximately 12 ml (Fig. 2C), suggesting an optimal composition of hOR, MSP, and lipid, yielding NDs with a desirable diameter of less than 20 nm (*53*–*55*). Further characterization of the NDs via dynamic light scattering analysis revealed a homogeneous size distribution, with an average diameter of 9.5 nm for the empty NDs (Fig. 2D); the hOR-containing NDs exhibited a larger size, approximately 16 nm, which was attributed to the presence of hORs (Fig. 2, E to G). The binding affinities between the hOR NDs and the target odorants (i.e., SCFAs) were evaluated using a tryptophan fluorescence quenching assay. Notably, each hOR ND displayed distinct binding affinities toward the SCFAs (Fig. 2, H to J).

To determine the functional stability of immobilized hOR NDs, which is essential for their reliability as a biomaterial in odorant detection, hOR51E2 ND was immobilized on a nickel-coated plate at 4°C for 16 hours. This was followed by rigorous washing with Hepes buffer II to eliminate unbound NDs. The immobilized NDs were then eluted with Hepes elution buffer after various incubation periods, and ND functionality was confirmed using the tryptophan fluorescence quenching assay with 1 mM PA as the target odorant (fig. S2A). Notably, hOR51E2 NDs maintained almost full functionality at 4°C and retained 50% functionality even after 16 weeks of incubation at 37°C, highlighting their exceptional stability (fig. S2B). Additional time-dependent stability tests at 37°C revealed a gradual decline in functionality between 1 and 5 hours, indicating that a rapid temperature transition from 4° to 37°C might adversely affect the stability and functionality of the hOR NDs (fig. S2C). Nevertheless, the immobilized hOR NDs exhibited remarkable stability, suggesting that they are suitable for integration into a bioelectronic nose for odorant sensing applications (*47*).

Upon assembly and purification, the hOR NDs were immobilized onto the graphene surface of the extended gate, providing functionality to the olfactory sensory neuron device (fig. S3). Field-emission scanning electron microscopy (SEM) images revealed that hOR NDs immobilized on the graphene surface exhibit a discoidal shape (fig. S5) (*43*, *46*, *48*). To demonstrate the sensing capabilities of olfactory sensory neuron device functionalized with hOR NDs, we conducted gas-sensing experiments by exposing 3 parts per million (ppm) of SCFAs during 50 s. After exposing gaseous SCFAs, pure N_2_ gas was exposed during 50 s to purge the surface of graphene. The concentration of SCFAs in the gas phase is regulated by controlling the mass flow of the nitrogen carrier gas (Fig. 2, K to N).

### Enhancing neural plasticity in organic synaptic devices

The transmission of activated signals from the olfactory sensory neuron device to postsynapse of the artificial synapse is a key step in the configuration of a neuromorphic system for high-precision odor recognition tasks (*49*). However, the nonlinearity of the conductance change in organic synaptic device (OSD) based on conventional organic electrochemical transistors constitutes a notable limitation because of challenges in maintaining the doping state of the semiconductor layer (*56*, *57*). This limitation arises from the fact that the doping state (induced by ion penetration in the channel layer) is easily erased upon removal of the presynaptic bias, as a result of the built-in potential (fig. S6A) (*58*). To address this limitation, we used a redox system that allows moderate control of the postsynaptic conductance (*G*_post_) by effectively trapping counter-ions (fig. S6B). Specifically, we functionalized the indium tin oxide (ITO) presynaptic electrode with α-MoO_3_, which can undergo reversible intercalation of lithium ions (Li^+^) through redox reactions (*59*–*61*). Subsequently, an ionic gel comprising poly(vinylidene fluoride-co-hexafluoropropylene) (PVDF-HFP) and lithium bis(trifluoromethanesulfonyl)imide (LiTFSI) salt, along with a poly(3-hexylthiophene-2,5-diyl) (P3HT) semiconductor, was sequentially spin-coated onto the α-MoO_3_ layer–coated presynaptic electrode (text S2 and figs. S7 to S16).

**Fig. 3.**
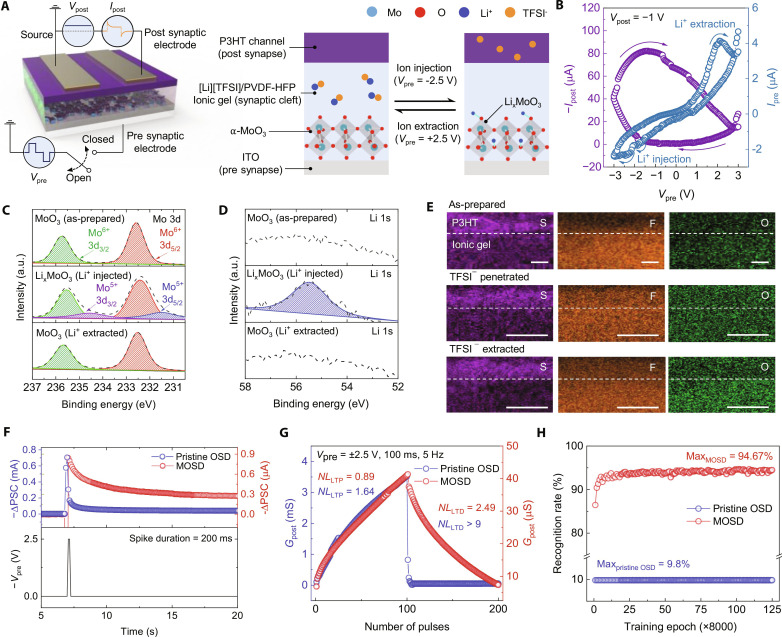
Characterization of the artificial synaptic device. (**A**) Geometrical structure and operational mechanism of the MOSD. (**B**) Transfer characteristics of the MOSD. (**C** and **D**) X-ray photoelectron spectroscopy analysis of MoO_3_ before and after lithiation/delithiation for the (C) Mo 3d and (D) Li 1s. a.u., arbitrary units. (**E**) Cross-sectional energy-dispersive x-ray spectroscopy mapping of S, F, and O elements in the organic semiconductor/ionic gel region. (**F**) EPSC responses of the pristine OSD and MOSD. (**G**) LTP/D characteristics of the pristine OSD and MOSD. (**H**) MNIST pattern-recognition accuracy of the pristine OSD and MOSD during 1 million training and inference cycles.

Figure 3A illustrates the geometric structure and operational mechanism of the artificial synapse. Specifically, when a sufficiently negative presynaptic bias (*V*_pre_) such as −2.5 V is applied, Li^+^ and TFSI^−^ ions migrate in opposite directions and infiltrate the α-MoO_3_ and P3HT layers, respectively. This migration leads to the formation of Li*_x_*MoO_3_, in which Li^+^ ions are tightly bound to the oxygen atoms of MoO_3_, hindering their diffusion out of the layer (*61*). To maintain charge neutrality in the ionic gel, TFSI^−^ ions also remain within the P3HT layer (*62*). Consequently, *G*_post_ increases; this increase persists even after the removal of *V*_pre_. The *G*_post_ can be moderately reduced by applying a sufficiently positive *V*_pre_, such as +2.5 V, which enables the extraction of Li^+^ and TFSI^−^ ions from the α-MoO_3_ and P3HT layers, respectively.

Figure 3B depicts the transfer characteristics of the MoO_3_-functionalized OSD (MOSD), where redox peaks related to the lithiation process (xLi^+^ + MoO_3_ + xe^−^ → Li*_x_*MoO_3_) and delithiation process (Li*_x_*MoO_3_ → xLi^+^ + MoO_3_ + xe^−^) are observed at −1.79, −2.3, and +2.1 V, respectively. To examine the elemental composition and chemical states of the α-MoO_3_ layer before and after the application of a *V*_pre_, we conducted x-ray photoelectron spectroscopy. The prepared α-MoO_3_ layer exhibited two distinct peaks at 232.62 and 235.72 eV, corresponding to Mo^6+^ 3d_5/2_ and 3d_3/2_, respectively; these results indicated a well-structured stoichiometric composition (Fig. 3C). After the lithiation process, reduced Mo^5+^ 3d_5/2_ and Mo^5+^ 3d_3/2_ peaks emerged at 231.55 and 234.55 eV, respectively, suggesting successful intercalation of Li^+^ ions into the α-MoO_3_ layer (*60*). Notably, these Mo^5+^ peaks vanished upon the extraction of Li^+^ ions, indicating that the intercalation process is reversible. In addition, the appearance and disappearance of the Li 1s peak upon consecutive injection and extraction of Li^+^ ions confirmed reversible intercalation in the α-MoO_3_ layer (Fig. 3D).

Using cross-sectional energy-dispersive x-ray spectroscopy mapping, we conducted further exploration of reversible molecular doping in the P3HT layer. By analyzing the relative distribution of fluorine and oxygen atoms, which are components of the TFSI^−^ ion, we visualized the penetration and extraction of TFSI^−^ ions in the P3HT layer and ionic gel (Fig. 3E). The results showed that the TFSI^−^ ions reversibly entered and exited the P3HT layer, maintaining the charge neutrality of the ionic gel.

The MOSD displayed enhanced relaxation characteristics of excitatory postsynaptic current (EPSC) compared with the pristine OSD (Fig. 3F). The EPSC memory of MOSD lasted for about 700 s (fig. S20). Notably, this result is different from the memory of pristine OSD, which lasted only around 5 s. The enhanced synaptic behaviors were supported by the relaxation of *G*_post_ and paired-pulse facilitation (PPF) behavior, an enhancement in the amplitude of the second of two rapidly evoked EPSC (figs. S20 and S21). These enhancements in synaptic performance were attributed to the relaxation capability of the doping state facilitated by lithiation.

We also explored the long-term potentiation/depression (LTP/D) characteristics of the MOSD and pristine OSD to assess their synaptic performance. A series of 100 consecutive potentiation pulses, followed by 100 consecutive depression pulses, was applied to the presynaptic electrode (Fig. 3G). The MOSD demonstrated superior neural plasticity, indicated by lower nonlinearity compared with the pristine OSD (fig. S22 and tables S1 to S3). We varied the operation conditions of LTP/D cycle of pristine OSD for optimized synaptic characteristic; however, the nonlinearities were not comparable with the one of MOSD (fig. S23 and S24). This result was attributed to the redox reaction between Li^+^ ions and α-MoO_3_. We provided detail synaptic performances of MOSD in figs. S25 to S28.

Last, we evaluated the performance of the pristine OSD and MOSD for training and inference tasks involving Modified National Institute of Standards and Technology (MNIST) digit patterns by using an ANN with the detailed architecture described in text S3 and fig. S29. After training with 1 million patterns, the MOSD exhibited a substantially enhanced recognition rate, with a maximum accuracy of 94.67%, whereas the pristine OSD achieved a maximum accuracy of 9.8% (Fig. 3H). This enhancement was attributed to the high conductance linearity of the MOSD, which resulted from controllable doping modulation.

### Convergence of human olfactory receptors and neuromorphic devices

**Fig. 4.**
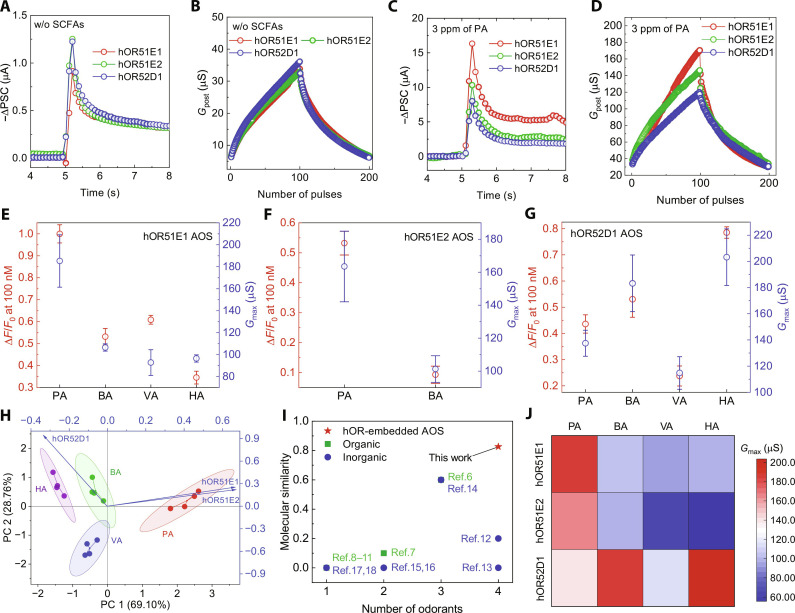
Characterization of the AOSs. (**A**) EPSC responses and (**B**) LTP/D responses of the AOSs without SCFAs. (**C**) EPSC responses and (**D**) LTP/D responses of the AOSs under 3 ppm PA conditions. For EPSC response, a *V*_pre_ pulse of −2.5 V was applied with a duration of 200 ms. For LTP/D responses, *V*_pre_ pulses of ±2.5 V were applied 100 times each with a duration of 100 ms and a frequency of 5 Hz. (**E** to **G**) Comparison between relative fluorescence intensity values at 100 nM of hOR NDs and conductance responses of (E) hOR51E1, (F) hOR51E2, and (G) hOR52D1 AOSs according to exposed SCFAs. Error bars represent standard error of the mean (*n* = 4). (**H**) Principal components (PC) analysis of SCFAs. Blue lines are extracted eigenvectors of PCA. (**I**) Comparison of reported neuromorphic gas sensors with our work in terms of odorant number and molecular similarity. (**J**) Patterned channel conductance signals of AOSs in response to 3 ppm of various SCFAs.

As described above, we successfully constructed an AOS by integrating the olfactory sensory neuron device with the MOSD. The detailed operating mechanisms of the AOS are described in text S4 and fig. S30. The stability of the AOS was enhanced by use thin gold wires and silver paste to create connections. In Fig. 4 (A and B), the EPSC and LTP/D characteristics of the AOSs were investigated without SCFAs exposure. Notably, there were no substantial differences in the EPSC values right after a *V*_pre_ pulse and retained efficacies after 3 s across the immobilized hOR NDs, and *G*_max_ values also showed no notable variance. However, when exposed to a nitrogen stream containing SCFAs by using gas generation system (fig. S31), the AOS showed notable differences in channel conductance response according to the type of immobilized hOR NDs.

For example, as shown in Fig. 4 (C and D), the AOSs demonstrated distinct EPSC and LTP/D characteristics in the presence of a nitrogen stream containing 3 ppm of PA. Specifically, the EPSC values right after a *V*_pre_ pulse were 16.33, 10.31, and 7.99 μA for hOR`51E1, hOR51E2, and hOR52D1 AOSs, respectively, and the relative order of retained efficacies maintained even after 3 s. The *G*_max_ values also showed distinct values with 170.3, 146.1, and 119.6 μS for hOR51E1, hOR51E2, and hOR52D1 AOSs, respectively, in accordance with the relative order of EPSC values. The AOSs exhibited differential synaptic behaviors including EPSC, inhibitory postsynaptic current, PPF, and paired-pulse depression to various SCFAs (figs. S32 to S38). On the basis of the *G*_max_ responses of AOSs, the detection limits were calculated from the calibration curves based on the *G*_max_ responses using the 3σ/*S* (fig. S39 and table S4). Each AOS showed various detection limits range from 0.07 to 1.30 ppm in response to SCFAs.

To determine whether these distinct conductance responses were caused by specific hOR-odorant binding interactions, we compared the relative fluorescence intensity at 100 nM of the hOR NDs with the conductance response of the AOS (Fig. 4, E to G). Intriguingly, the trends in relative fluorescence intensity and *G*_max_ values of the hOR NDs in relation to each SCFA were consistent, suggesting that the SCFA-dependent conductance response of the AOS was caused by specific hOR-odorant interactions. This alignment confirmed the successful transference of the functionalities of the hOR NDs to the AOS.

Furthermore, to analyze odorant patterns, we performed principal components (PC) analysis using the collected *G*_max_ data under 3 ppm of various SCFAs (Fig. 4H and tables S5 and S6). The first two PCs (PC1 and PC2) explained 97.9% of the total variance, encompassing most of the original variables. Notably, the SCFAs displayed distinct characteristics, enabling identification of each SCFA via PC analysis.

To compare the capacity of our AOS to identify odorants, relative to previously reported neuromorphic gas sensors, we evaluated the molecular similarity of the odorants used in each study by calculating the molecular similarity coefficient. The maximum molecular similarity of our hOR-embedded AOS surpassed all previously reported multivariable neuromorphic gas sensors and was comparable with the highest values of other sensor platforms (Fig. 4I, fig. S40, and tables S7 and S8).

Moreover, by assembling the *G*_max_ responses of the AOS based on the SCFAs, we generated unique combinatorial patterns (Fig. 4J), which simulate the data assembly process that occurs in the glomerulus during odor recognition. Using these combinatorial patterns, we performed training and inference simulations to emulate the odor recognition capability of the human olfactory system.

### Mimicking the odor recognition capability of the human olfactory system

**Fig. 5.**
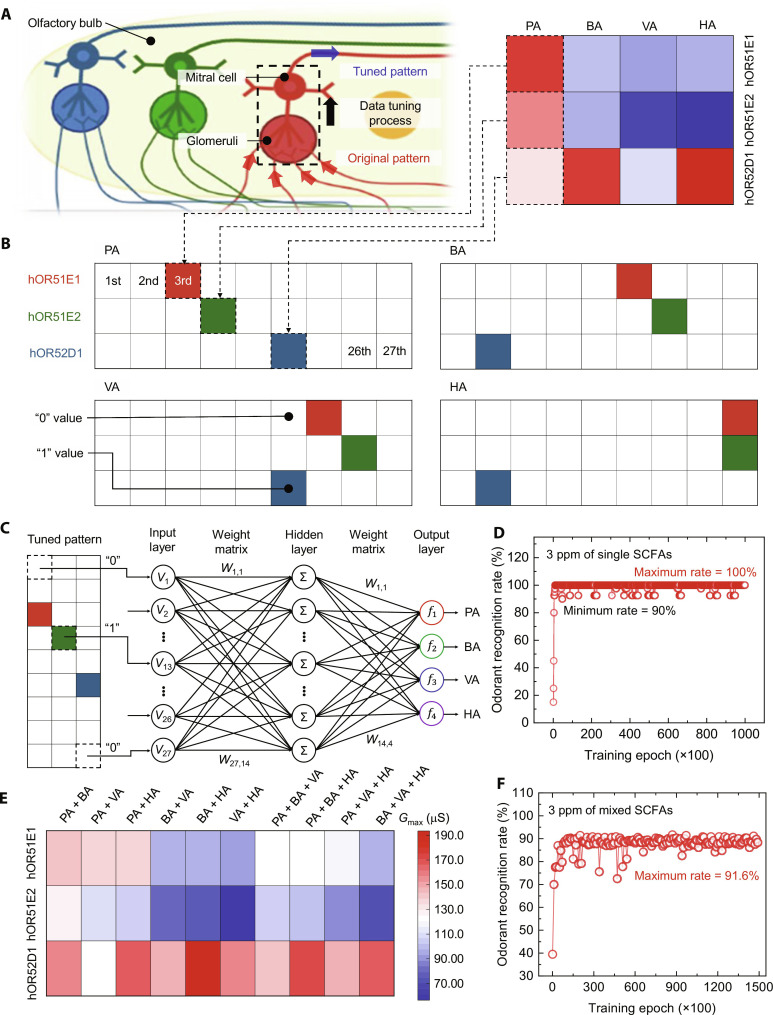
Mimicking odorant recognition capability using ANNs. (**A**) Schematic illustration of the data tuning process in the glomerulus and mitral cells. (**B**) Tuned patterns of SCFAs. (**C**) Theoretical ANN with 27 input neurons, 14 hidden neurons, 4 output neurons, and 1512 synapses. (**D**) Single-odorant recognition accuracy during 100,000 training and inference cycles. (**E**) Theoretical conductance patterns of mixed odorants. (**F**) Mixed-odorant recognition accuracy during 150,000 training and inference cycles.

We have demonstrated the odor recognition capability of the AOS through the use of a training and inference simulation approach, using channel conductance patterns derived from sensing experiments with 3 ppm of SCFAs. Inspired by the signal organization process within the glomerulus and mitral cells of the human olfactory system, we engineered a refinement process for the channel conductance patterns to achieve highly precise odor recognition (Fig. 5A). The glomerulus takes in electrical signals formed as patterns that arise from interactions between hORs and odorants in the olfactory sensory neurons. Mitral cells receive these patterns from the olfactory sensory neurons, then refine the patterns to bolster odor recognition. The signals are then relayed to the olfactory cortex for additional data processing.

In our simulation, we segmented the conductance into nine regions and assigned each conductance value to the corresponding region (text S6 and table S9). This resulted in a unique odorant representation in the form of a 9 × 3 array, as shown in Fig. 5B. This refinement process enhanced the original combinatorial patterns into more feature-rich patterns that conveyed distinct information about the odorants (figs. S41 to S43).

For the ANN simulation, we compiled datasets consisting of 216 training patterns and 40 inference patterns for SCFAs (tables S10 to S13). The custom-designed ANN included 27 input neurons, which corresponded to the 9 × 3 array pixels of the odorant representations, 14 hidden neurons, 4 output neurons, and 27 × 14 × 4 artificial synapses that connected the neurons (Fig. 5C).

We conducted training and inference using the refined patterns and the designed ANN, then graphed the recognition rate at intervals of 100 training steps (one epoch), as illustrated in Fig. 5D. Notably, within 100 training steps, the accuracy of odor recognition reached 100%. This high accuracy was attributed to the specific hOR-odorant binding interactions and the synaptic device used in our AOS, which exhibits high linearity in terms of modulating synaptic weight. After 1000 training steps, the maximum and minimum odorant recognition rates were 100 and 90%, respectively. In addition, we tested whether the AOSs have the capability to discriminate odorants in different concentrations. Our AOSs successfully discriminated the odorants in not only based on molecular chain length but also on concentration levels (fig. S44 and tables S14 to S17).

To more fully explore the capabilities of our AOS, we generated theoretical mixed-odorant patterns using the conductance patterns of single odorants obtained from sensing experiments. For the mixtures, the total odorant concentration of the theoretical mixture is 3 ppm, and the mixture contains each odorant with same concentration. Each odorant signal was supposed to be 1/*n* of the total signal, where “*n*” is the number of odorants in the mixture. The final signal of the theoretical mixture was assumed to be the sum of each odorant signal (text S6). Consequently, we generated combinatorial patterns for six different mixtures of two odorants and four different mixtures of three odorants (Fig. 5E).

For the ANN simulation, datasets comprising 2194 training patterns and 366 inference patterns for nine SCFAs were prepared, using the refinement process that had been applied to single-odorant patterns. The ANN tailored for mixed-odorant patterns comprised 27 input neurons, which corresponded to the 9 × 3 array pixels of the mixed-odorant images, 27 hidden neurons, 9 output neurons, and 27 × 27 × 9 artificial synapses connecting the neurons. We conducted training and inference using these patterns and the customized ANN, then graphed the recognition rate at intervals of 100 training steps (one epoch) (Fig. 5F). Notably, over 150,000 training steps, the peak accuracy reached 91.6%.

## DISCUSSION

We demonstrated the convergence of hORs with an artificial synapse, highlighting its exceptional odorant recognition capability at the molecular chain length level. Three distinct hORs were reconstituted into NDs and immobilized on a graphene extended-gate device, effectively assembling an olfactory sensory neuron platform. Notably, the hOR NDs displayed various levels of sensitivity to four different SCFAs, which are regarded as essential diagnostic biomarkers for gastric cancer and halitosis. To improve the neural plasticity of the artificial synapse, we integrated an α-MoO_3_–based redox system, which achieved enhanced conductance linearity in MOSD compared with the pristine OSD. Using the features of both the olfactory sensory neuron device and MOSD, we successfully incorporated them into the AOS. This AOS demonstrated distinct channel conductance patterns based on the exposed SCFAs, leading to the generation of combinatorial patterns with unique features. Furthermore, we simulated the odorant recognition capability of the human olfactory system through the developed AOS and ANN simulation, achieving remarkable precision in differentiating four different SCFAs and their combinations at the chain length level (90 to 100%). The developed AOS represents an efficient platform for future sensor technologies; it also lays a foundation for artificial sensory systems that can be integrated into wearable and implantable devices, heralding new possibilities in the field of neuromorphic sensors.

## MATERIALS AND METHODS

### Cloning of hOR genes into a bacterial expression vector

Primers were designed to amplify hOR genes from human genomic DNA. For hOR51E1, the forward primer was 5′-CAC CAG GAG ATA TAC ATA TGA TGG TGG ATC CC-3′ and the reverse primer was 5′-GGG CTC TGA AGC GT-3′. For hOR51E2, the forward primer was 5′-CAC CAG GAG ATA TAC ATA TGA GTT CCT GCA ACT TCA CA-3′ and the reverse primer was 5′-CTT GCC TCC CAC AGC C-3′. For hOR52D1, the forward primer was 5′-CAC CAG GAG ATA TAC ATA TGT CAG ATT CCA ACC TC-3′ and the reverse primer was 5′-TAT TGA AGT CTT CCC CA-3′. Polymerase chain reaction–amplified products were cloned into the pET-DEST42 bacterial expression vector (Invitrogen, USA) using the Gateway cloning system (Invitrogen, USA).

### Expression and purification of hORs in *E. coli*

The pET-DEST42/hOR vector was transformed into the SuptoxR strain, which was the Rosetta 2 (DE3) *E. coli* strain (Merck, USA) that coexpresses RraA to inhibit mRNA degradation by *E. coli* RNase E. This facilitates overexpression of the hOR by suppressing membrane protein–induced toxicity. The transformed *E. coli* was cultured on agar plates containing ampicillin (100 μg/ml) and chloramphenicol (40 μg/ml) for 16 hours at 37°C. A single colony was then inoculated into 5 ml of LB medium with the same antibiotics and incubated for 16 hours at 37°C. Next, 250 μl of this culture were inoculated into 250 ml of LB medium with the same antibiotics and incubated for 16 hours at 37°C. The bacterial cells were transferred to 1 liter of fresh LB medium, and expression of RraA was induced with 0.2% arabinose. The culture was incubated until it reached an optical density (OD_600_) of 0.3 to 0.5 at 30°C. The temperature was then decreased to 25°C over 1 hour before the addition of 0.1 mM isopropyl-β-d-thiogalactopyranoside to induce hOR expression. After 16 hours of incubation at 25°C, the cells were harvested by centrifugation and resuspended in phosphate-buffered saline with 2 mM EDTA (pH 7.4). Subsequently, they were sonicated to release insoluble proteins. The insoluble proteins were solubilized by incubation with a solubilization buffer (0.1 M Tris-HCl, 20 mM SDS, 1 mM EDTA, and 0.1 M dithiothreitol, pH 8.0) overnight at 30°C. The solubilized proteins were separated by centrifugation and dialyzed using dialysis membrane tubing (MEMBRA-CEL, 14 kDa cutoff) with binding buffer (0.1 M sodium phosphate and 10 mM SDS, pH 8.0). The hORs were purified using a HisTrap HP column (GE Healthcare) with washing and elution buffers. The buffers for purified hORs were exchanged with Hepes buffer I (20 mM Hepes, 100 mM NaCl, and 25 mM cholate, pH 8.0) using a HiTrap Desalting column (GE Healthcare) in preparation for ND assembly.

### Assembly and purification of hOR-embedded NDs

1,2-Dimyristoyl-sn-glycero-3-phosphocholine (DMPC) (20 mM) in Hepes buffer (20 mM Hepes, 100 mM NaCl, and 50 mM cholate, pH 8.0) was used for assembly of the OR-embedded NDs. MSP1E3D1 was added to the DMPC solution at a 1:150 molar ratio, then incubated for 10 min at 24°C. Subsequently, the OR in Hepes buffer I was added, and incubation continued for 2 hours at 24°C. The final molar ratio of OR, MSP1E3D1, and DMPC was 1:5:750. Biobeads were added to remove cholate, and the mixture was incubated overnight at 24°C. Empty NDs were removed using a HisTrap HP column; the hOR-embedded NDs were purified with Hepes elution buffer (20 mM Hepes, 100 mM NaCl, and 350 mM imidazole, pH 8.0) via size exclusion chromatography (Superdex 200 Increase 10/300 GL, GE Healthcare) in Hepes buffer II (20 mM Hepes and 100 mM NaCl, pH 8.0).

### Tryptophan fluorescence quenching assay

The functionality of the OR-embedded NDs was analyzed using a tryptophan fluorescence quenching assay with a luminescence spectrometer (LS 55 Luminescence Spectrometer, PerkinElmer). Excitation and emission wavelengths were set at 280 and 333 nm, respectively. Normalized fluorescence intensity was calculated using the following equation

$$\frac{∆F}{F_{0}}\left(\%\right)=\frac{\left(F_{0}-F\right)}{F_{0}}\times 100\left(\%\right)$$

where *F*_0_ is the fluorescence intensity of the odorant-untreated NDs (500 nM), and *F* is the fluorescence intensity of the odorant-treated NDs.

### Fabrication of the graphene extended-gate device

A graphene film was synthesized on copper foil (25 μm thickness, Alfa Aesar) using a chemical vapor deposition method with H_2_ and CH_4_ gases. After graphene growth, the surface was coated with poly(methyl methacrylate) (PMMA) (950K A4, Microchem). The copper foil was subsequently etched away using an aqueous solution of ammonium persulfate (0.1 M, Sigma-Aldrich). The PMMA/graphene layer was then transferred onto a SiO_2_ (300 nm)/*n*^++^Si wafer through a wet transfer process; it was allowed to dry at room temperature for 6 hours. The PMMA was removed with chloroform. Chromium (4 nm) and gold (40 nm) were thermally evaporated onto the graphene/substrate in sequence to form the source and drain electrodes through shadow masks.

### Immobilization of hOR NDs on graphene

For the immobilization of hOR NDs on the graphene surface, 10 μl of 1 mM 1-pyrenebutyric acid *N*-hydroxysuccinimide ester (PSE) in methanol were applied to the graphene extended-gate device for 1 hour. The graphene was then washed two to three times with methanol to remove excess PSE. Methanol was subsequently evaporated by purging with nitrogen gas. Next, 10 μl of 1 μM olfactory receptor–embedded NDs was applied to the graphene and incubated for 4 hours at room temperature. The substrate was then washed two to three times with ultrapure water to remove unbound NDs. Last, any remaining solvent was evaporated by purging with nitrogen gas until the graphene surface was dry.

### Fabrication of the artificial synaptic device

ITO-coated glass substrates were sequentially cleaned with deionized water, acetone, and isopropyl alcohol. On the cleaned ITO/glass substrate, 100 nm of molybdenum trioxide (MoO_3_) (Alfa Aesar) was thermally evaporated. The MoO_3_/ITO/glass substrate was then annealed at 350°C for 6 hours under ambient conditions. An ionic gel solution consisting PVDF-HFP (Sigma-Aldrich), LiTFSI salt (Sigma-Aldrich), and acetone (Sigma-Aldrich) in an 8:1:56 ratio was prepared. Subsequently, this ionic gel solution was spin-coated at 1500 rpm for 60 s. After a bake at 80°C for 2 hours, a solution of P3HT (10 mg/ml; EMNI) was spin-coated at 1500 rpm for 60 s. After another bake at 120°C for 30 min, gold (40 nm) was thermally evaporated to form the source and drain electrodes through shadow masks. The fabricated synaptic device was electrically connected to the olfactory sensory neuron device using silver paste and gold wire.

### Characterization of the artificial synaptic device

Noncontact mode atomic force microscopy (AFM) images were captured using an NX-10 system (Park Systems) housed at the Research Institute of Advanced Materials at Seoul National University. The AFM images were analyzed with XEI software. X-ray diffraction analyses were conducted using a Smart Lab X-ray diffractometer (Rigaku) with Cu Kα radiation (λ = 1.5406 Å) at 40 kV/40 mA. SEM micrographs were captured using a JSM-7800F Prime (JEOL) field-emission scanning electron microscope. Raman spectroscopy was performed with a He-Ne laser (532 nm) as the excitation source, using confocal Raman microscopy (Alpha 300S, WITec, Germany). The elemental composition of MoO_3_ was determined via x-ray photoelectron spectroscopy at the National Center for Inter-University Research Facilities at Seoul National University. Electrical characteristics of the synaptic device and AOS were measured under ambient conditions and nitrogen gas flow using a Keithley 4200-SCS semiconductor parametric analyzer.

### Sample preparation for XPS and UV/Vis spectroscopy of MoO_3_

First, all the samples were fabricated by introducing MoO_3_, ionic gel, organic semiconductor, and Au electrodes sequentially on ITO-coated glass substrates as described in experimental section. After the fabrication processes, the “as-prepared” sample was prepared by delaminating the electrodes/organic semiconductor/ionic gel layers. Owing to the solid-like property of the ionic gel, entire layers above the MoO_3_ can be peeled off smoothly (fig. S17). For the “Li^+^ ion–injected” (Li*_x_*MoO_3_) sample, the same process as the as-prepared MoO_3_ sample was conducted and subsequently subjected to lithiation. We applied 1000 pulses of *V*_pre_ (−2.5 V, 100 ms, and 5 Hz) for lithiation of MoO_3_ layer, until the MoO_3_ layer became navy blue enough. The enhanced absorbance at a higher wavelength means that MoO_3_ layer is electrochemically doped (fig. S18). Then, we delaminated the layers above the MoO_3_ using a tweezer. For the “Li^+^ ion–extracted” (MoO_3_) sample, we first applied 1000 pulses of *V*_pre_ (−2.5 V, 100 ms, and 5 Hz) for lithiation of MoO_3_ layer, until the layer became lithiated enough. Then, 1000 pulses of *V*_pre_ (+2.5 V, 100 ms, and 5 Hz) for delithiation of Li*_x_*MoO_3_ were applied until the layer became transparent enough. Last, we delaminated the layers above the MoO_3_ using a tweezer.

### Calculation of artificial synaptic device non-linearity

The nonlinearity of LTP/D (100 consecutive potentiation pulses, followed by 100 consecutive depression pulses) was calculated using the following equations

$$G_{\text{LTP}}=B\times [1-\text{exp}\left(-\frac{P}{A_{P}}\right)]+G_{\text{min}}$$

$$G_{\text{LTD}}=-B\times \{1-\text{exp}[\frac{\left(P-P_{\text{max}}\right)}{A_{D}}]\}+G_{\text{min}}$$

$$B=\frac{\left(G_{\text{max}}-G_{\text{min}}\right)}{\left[1-\text{exp}\left(\frac{-P_{\text{max}}}{A_{P,D}}\right)\right]}$$

where *G*_LTP_ and *G*_LTD_ represent the conductance values of LTP/D, respectively, *P* is the number of applied maximum pulses, *A* is a nonlinearity value extracted from MATLAB code, and *B* is a fitting constant.

### Preparation of gas sensing samples

SCFAs gas samples were prepared at a concentration of 3 ppm in a nitrogen gas stream. Nitrogen gas was bubbled through a vessel containing SCFAs in liquid form, allowing evaporation of the SCFAs. The concentration of gaseous SCFAs was controlled by adjusting the temperature of the liquid SCFAs. A three-way valve was used to switch the flow of gas from a pure nitrogen source to the evaporated SCFAs while maintaining the same pressure and flow rate.

### Odorant recognition test via two-layer neural network simulation

The simulation was performed using the “MLP + NeuroSim ver. 3.0,” which is a software platform used to test the performance of neuromorphic hardware. A theoretical two-layer perceptron-based ANN with a size of 27 × 14 × 4 (27 × 27 × 10 for mixed odorants) was constructed. Then, 9 × 3 combinatory odorant patterns, binarized to black-and-white (1 and 0) patterns, were transmitted to the input layer. The optimized learning rates for the first and second synaptic weight matrices were 0.03 and 0.0001, respectively.

## Supplementary Materials

### This PDF file includes:

Text S1 to S8 Figs. S1 to S46 Tables S1 to S17 Legends for data S1 to S4 References
