# Supplementary material for: A pattern recognition artificial olfactory system based on human olfactory receptors and organic synaptic devices
Source: Sci Adv. 2024 May 23;10(21):eadl2882. doi: 10.1126/sciadv.adl2882 (PMC11114221; doi:10.1126/sciadv.adl2882)
Supplement: Supplementary file 1 — Text S1 to S8 Figs. S1 to S46 Tables S1 to S17 Legends for data S1 to S4 References [file sciadv.adl2882_sm.pdf]

Science Advances
AAAS

Supplementary Materials for

A pattern recognition artificial olfactory system based on human olfactory receptors and organic synaptic devices

Hyun Woo Song *et al.*

Corresponding author: Joon Hak Oh, joonhoh@snu.ac.kr; Tai Hyun Park, thpark@ewha.ac.kr

Sci. Adv. **10**, eadl2882 (2024)
DOI: 10.1126/sciadv.adl2882

The PDF file includes:

Text S1 to S8
Figs. S1 to S46
Tables S1 to S17
Legends for data S1 to S4
References

Other Supplementary Material for this manuscript includes the following:

Data S1 to S4

Supplementary Text 1. Necessity and importance of short-chain fatty acids as target molecules

Short-chain fatty acids (SCFAs) are valuable diagnostic biomarkers for gastric cancer and halitosis (*63-67*). Numerous studies suggest that SCFAs serve as potent indicators in the exhaled breath of patients with stomach cancer. Cross-platform validation has confirmed that butyric acid, valeric acid, and hexanoic acid are biomarkers of gastrointestinal cancer in the breath. Additionally, SCFAs are the primary factors contributing to halitosis, which arises from microbial activities in biofilms within the oral cavity. Notably, the majority of individuals with halitosis (approximately 80-90%) experience oral malodor (*68-70*).

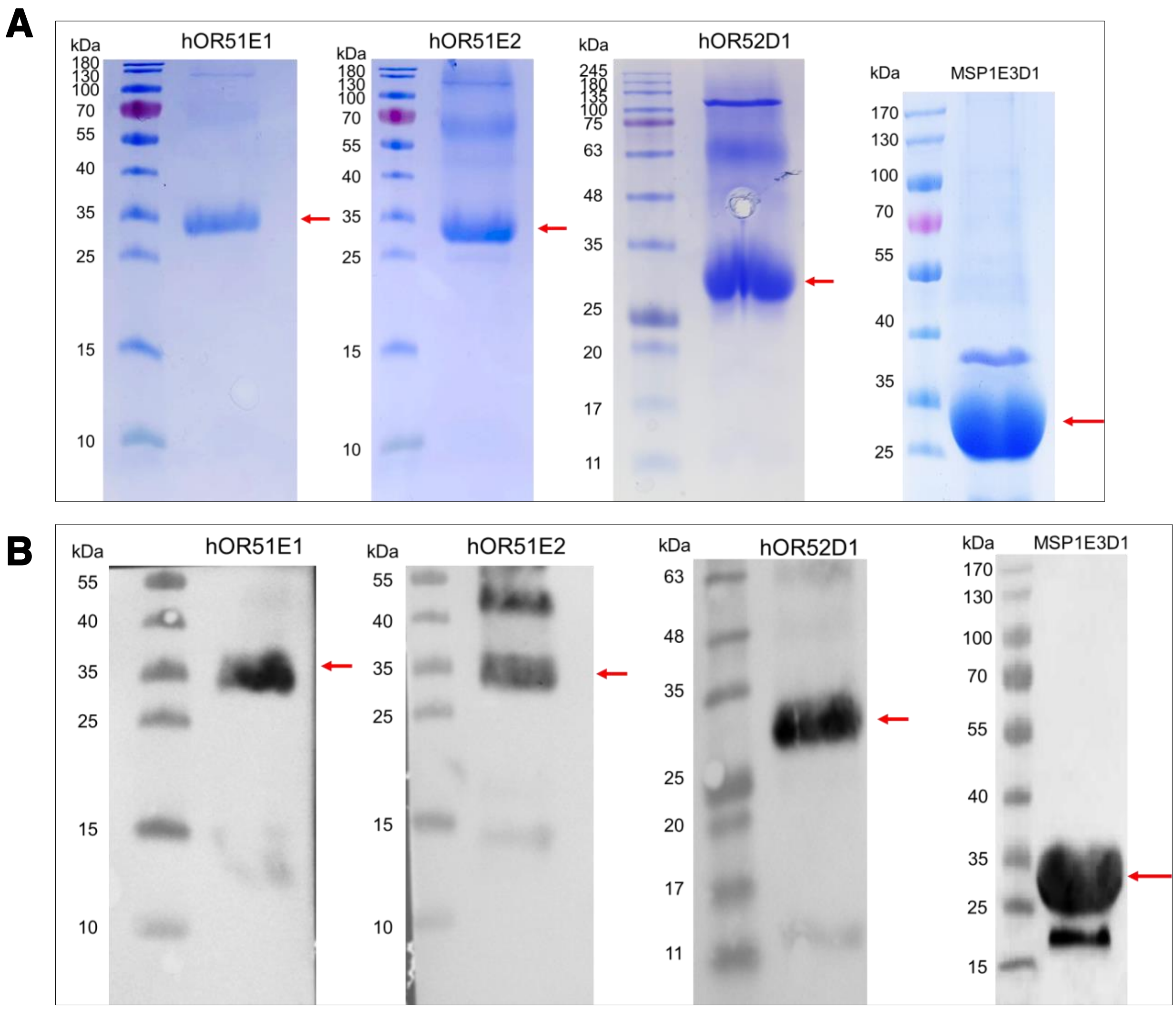

Fig. S1. Overexpression of hORs and MSP in *E. coli* system. **(A)** Coomassie blue staining analysis of purified hOR51E1, hOR51E2, hOR52D1, and MSP1E3D1. **(B)** Western blotting analysis of purified hOR51E1, hOR51E2, hOR52D1, and MSP1E3D1, using an anti-His tag antibody as the primary antibody for detection of hORs and MSP.

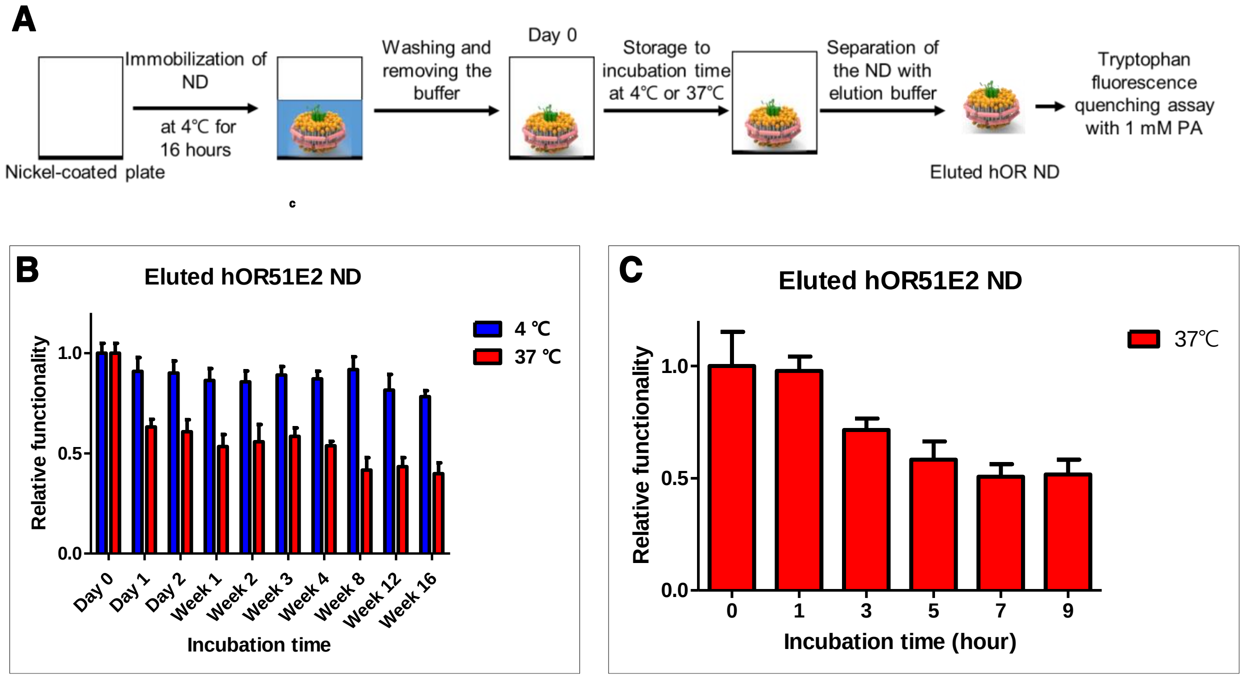

Fig. S2. Functional stability of immobilized hOR ND. (A) Schematic diagram illustrating the stability test of immobilized hOR ND. **(B)** Stability test of immobilized hOR51E2 ND conducted over 16 weeks at 4°C and 37°C. **(C)** Stability test of immobilized hOR51E2 ND conducted over 9 h at 37°C. Error bars represent standard error of the mean (n = 5).

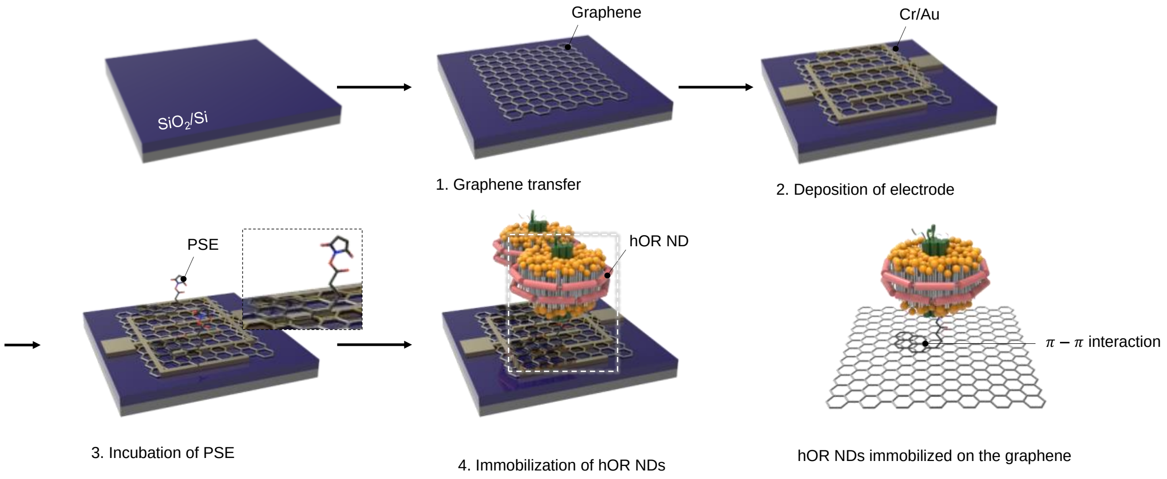

Fig. S3. Fabrication process for hOR ND-immobilized graphene device. Fabrication begins by transferring graphene onto a SiO_2/Si wafer. Chromium and gold are thermally evaporated in sequence onto the graphene/substrate to form the source and drain electrodes through shadow masks. Next, 1-pyrenebutyric acid N-hydroxysuccinimide ester (PSE) is loaded onto graphene via $\pi - \pi$ interactions. Finally, the hOR NDs are incubated and immobilized on the graphene channel.

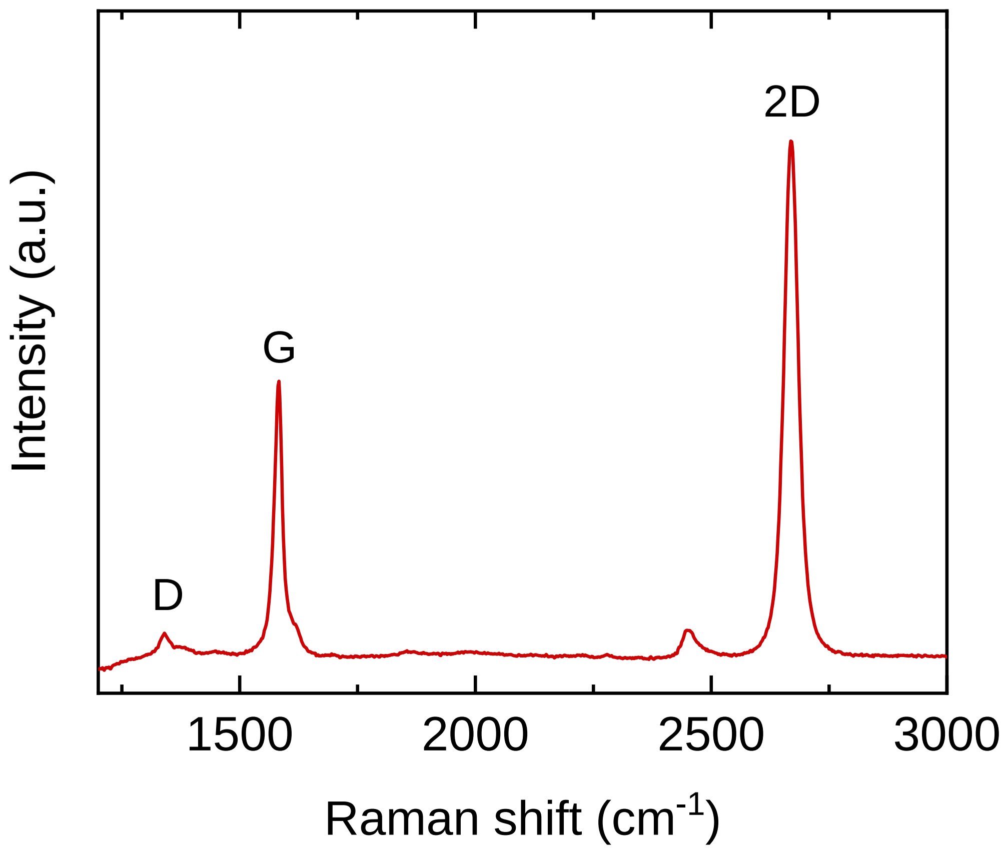

Fig. S4. Raman spectroscopy images of chemical vapor deposition-grown graphene. Distinct features are present, including the G peak at 1580 cm^{-1} arising from a primary in-plane vibrational mode and the 2D peak at 2690 cm^{-1} arising from a second-order overtone of a different in-plane vibration.

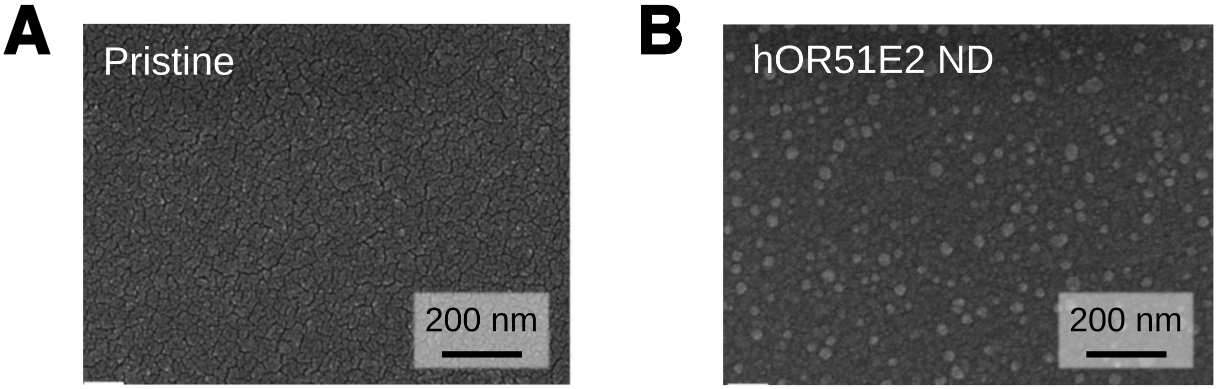

Fig. S5. FE-SEM images of graphene surface. (A, B) SEM images of (A) pristine graphene not treated with hOR51E2 ND and (B) graphene treated with hOR51E2 ND.

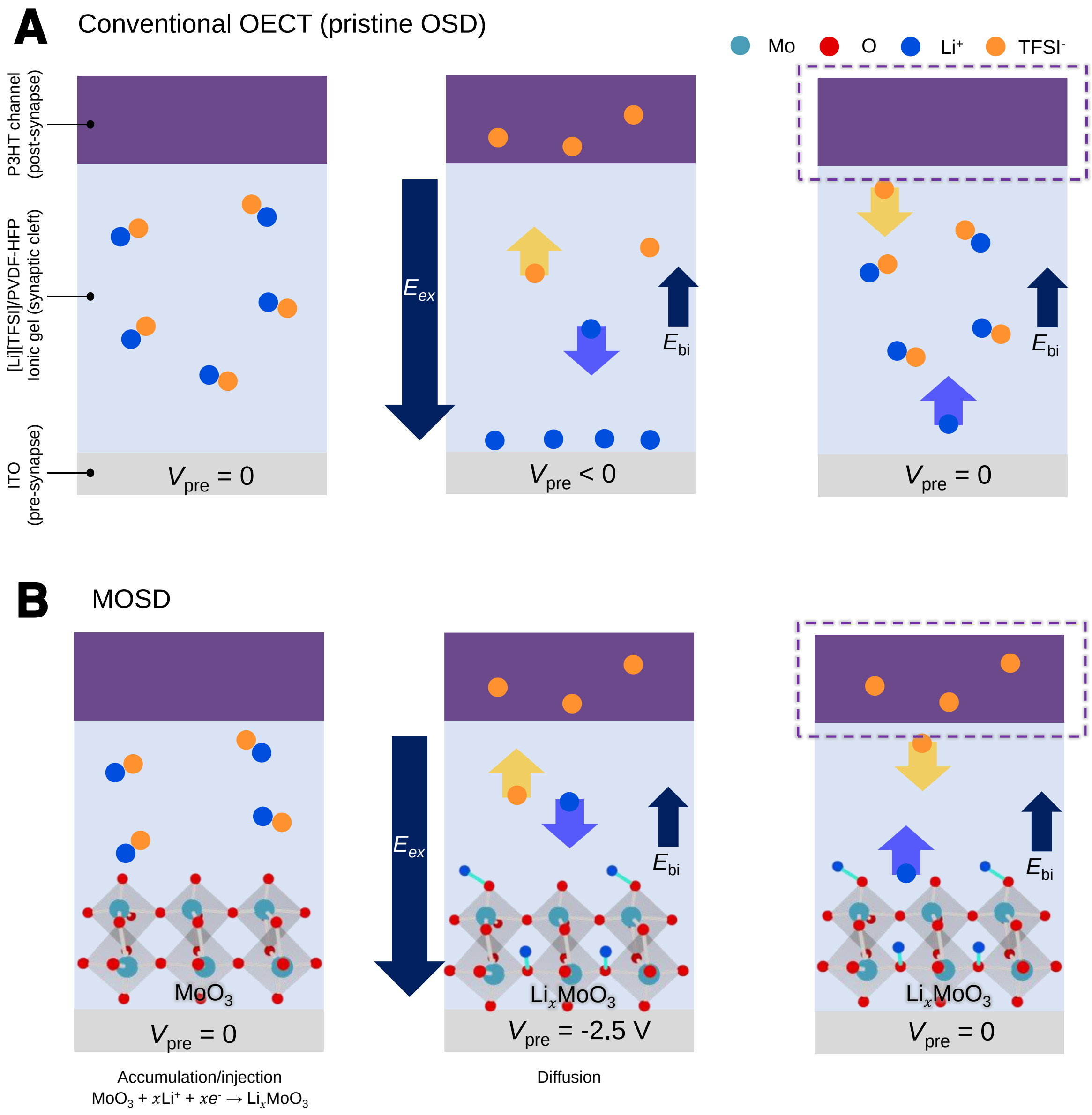

Fig. S6. Schematic illustrations of device structures of pristine organic synaptic device (OSD) and MoO_3-functionalized organic synaptic device (MOSD) in relation to pre-synaptic voltage (V_{pre}). (A) After removal of the negative V_{pre}, the doping state of the post-synapse is erased because of built-in potential (E_{bi})-induced ion drift. **(B)** Even after removal of the negative V_{pre}, the doping state of the post-synapse persists because only accumulated ions can be driven into ionic gels by E_{bi}.

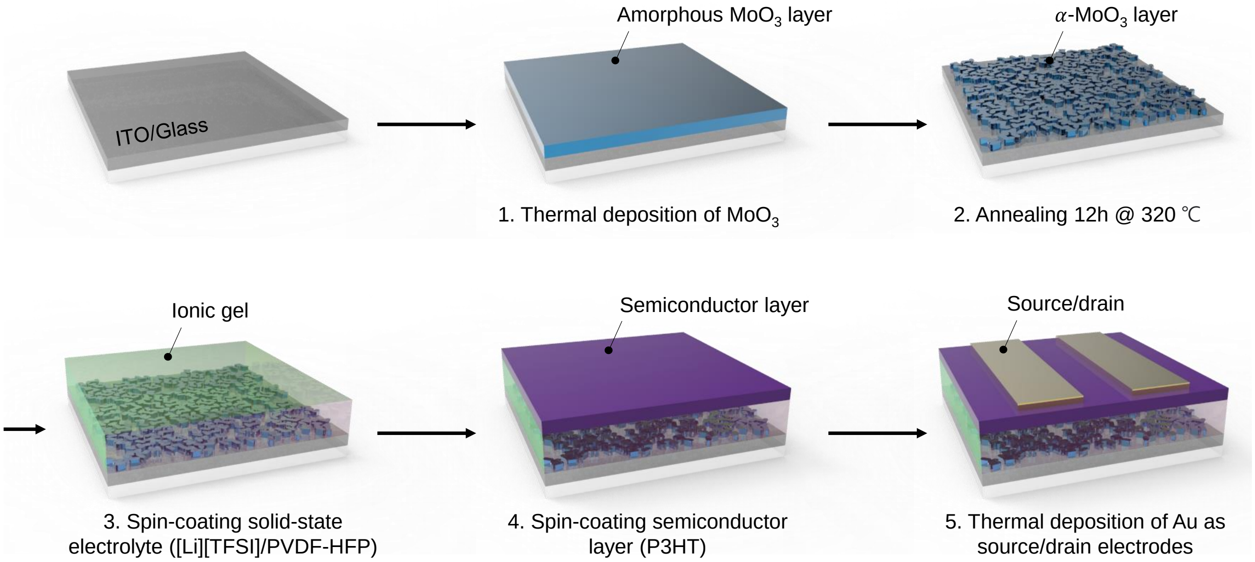

Fig. S7. Fabrication process for MOSD. The process begins with ITO/glass that is thermally deposited with MoO_3. Next, the MoO_3/ITO/glass is annealed in ambient conditions to crystallize the amorphous MoO_3 into an orthorhombic structure (α-MoO_3). Next, ionic gel and semiconducting layer are sequentially deposited by spin coating. Finally, gold is thermally evaporated to form the source and drain electrodes through shadow masks.

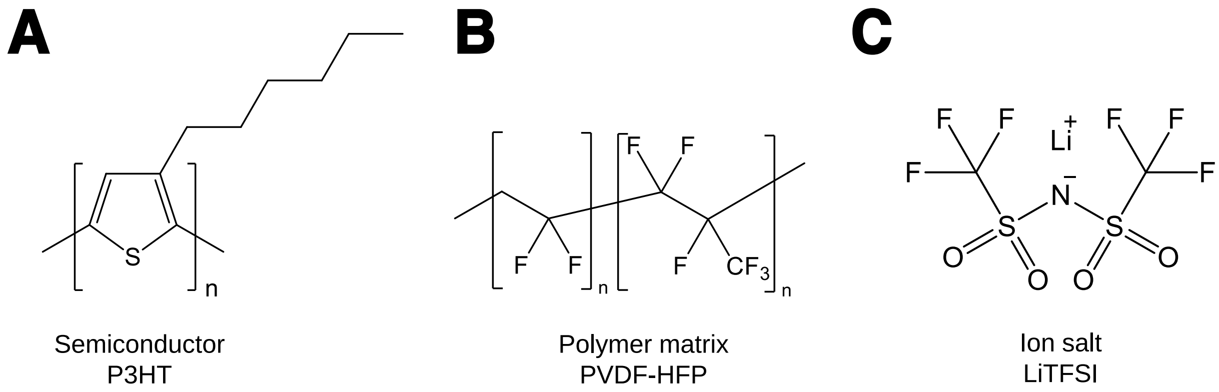

Fig. S8. Molecular structures of materials used in MOSD fabrication. (A to **C),** Molecular structures of (A) Poly(3-hexylthiophene-2,5-diyl) (P3HT)**,** (B) Poly(vinylidene fluoride-co-hexafluoropropylene) (PVDF-HFP)**,** and (C) Lithium bis(trifluoromethanesulfon-yl)imide (LiTFSI).

Supplementary Text 2. Characterization of orthorhombic α-MoO_3

We coated orthorhombic MoO_3 (α-MoO_3) on the pre-synaptic electrode (ITO) as the redox-active material using the thermal evaporation and annealing method. α-MoO_3 is a layered two-dimensional (2D) material capable of reversible intercalation by lithium ions (Li^+) through redox reactions. Previous researches have explored the use of α-MoO_3 nanosheets as the redox-active channel material; however, it has been challenging to integrate them with large-scale devices (*71, 72*). Our approach, which involves applying an α-MoO_3 layer through thermal evaporation and annealing, can be optimized for compatibility with contemporary chip fabrication processes. **(Fig. S9)** Moreover, the use of MoO_3 as a gate-functionalizing material, rather than a channel material, provides greater versatility in the selection of channel materials (*61*).

We characterized the MoO_3 layer before and after the annealing process. Before annealing, no grains were observable under an optical microscope or atomic force microscope. After annealing, the layer displayed anisotropic grain-like structures (**Fig. S10, 11**). We used a scanning electron microscope (SEM) to examine the structure of the grains; the images revealed that the grains were composed of layered 2D structures (**Fig. S12, 13**), consistent with the intrinsic properties of α-MoO_3 (*73*).

X-ray diffraction analysis was conducted to investigate the physical structure of the prepared MoO_3 (**Fig. S14**) (*74*). Peaks observed at 12.8, 25.7, and 38.8 were identified as the (020), (040), and (060) planes, respectively. These findings, along with information from JCPSD card no: 05-508, confirmed that the MoO_3 had an orthorhombic structure. The X-ray diffraction results were consistent with the SEM images, indicating that the grains exhibited anisotropic structures with strong preferential orientations.

We then explored the chemical composition of the prepared α-MoO_3 using Raman spectroscopy and X-ray photoelectron spectroscopy. The Raman spectroscopy results showed five major peaks **(Fig. S15)**. The peak at 995 cm^{-1} was attributed to the Mo=O asymmetric stretching modes of terminal oxygen. The most intense peak at 813 cm^{-1} was attributed to the bridge-oxygen Mo_2–O stretching modes of doubly connected oxygen, resulting from corner-shared oxygen atoms common to two MoO_6 octahedra. The peak at 669 cm^{-1} was attributed to the Mo_3–O stretching modes of triply connected bridge-oxygen, arising from edge-shared oxygen atoms common to three octahedra. Additionally, there were O=Mo=O wagging modes at 287 cm^{-1} and Tb modes at 158 cm^{-1}

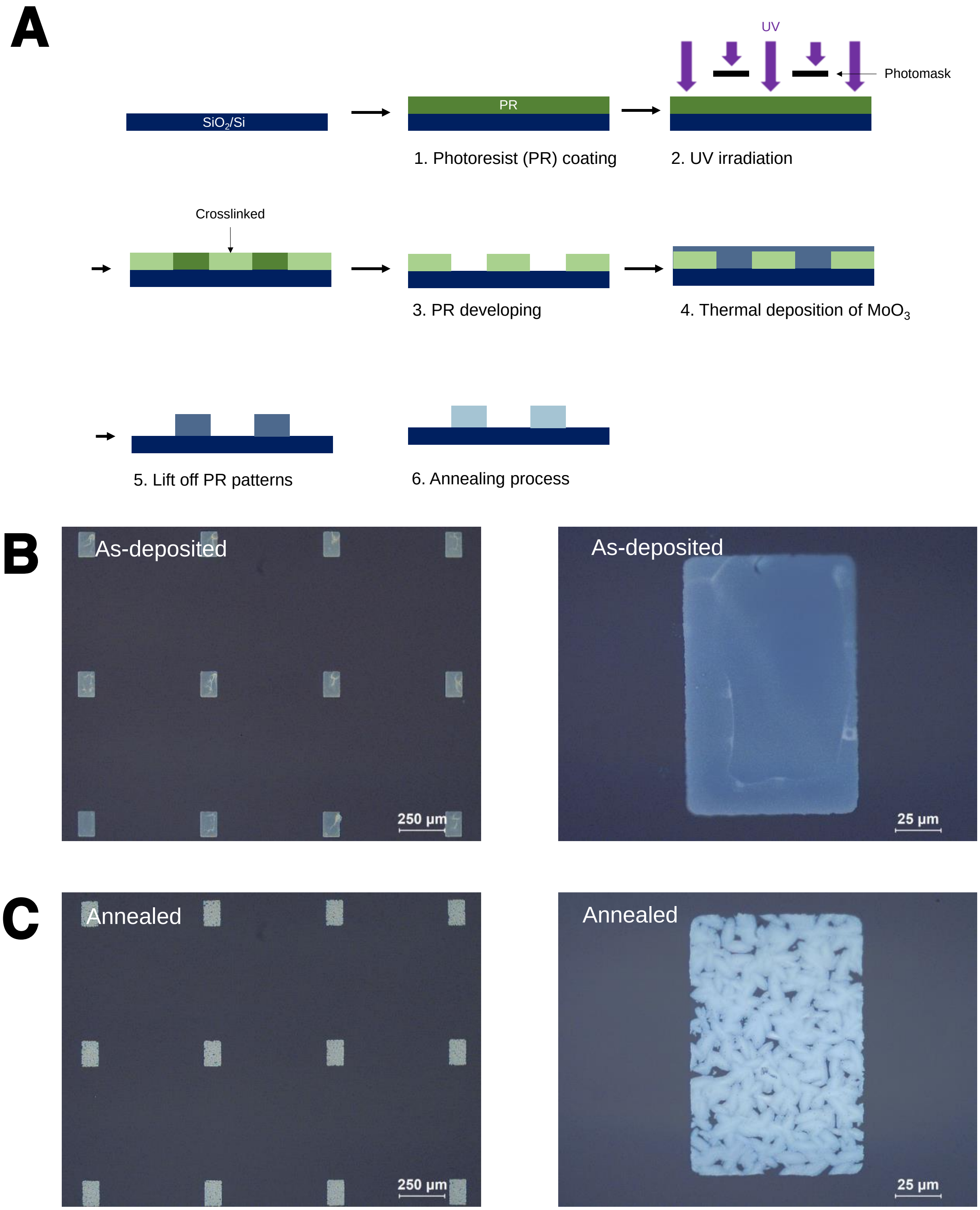

Fig. S9. Micropatterning of α-MoO_3 via conventional photolithography. (A) Micropatterning of MoO_3 using lift-off process. **(B)** Patterns of amorphous MoO_3 after lift-off process. **(C)** Microscale α-MoO_3 patterns after annealing of amorphous MoO_3 patterns. Elliptical grain structures, characteristic of orthorhombic $MoO_{3,}$ are observed.

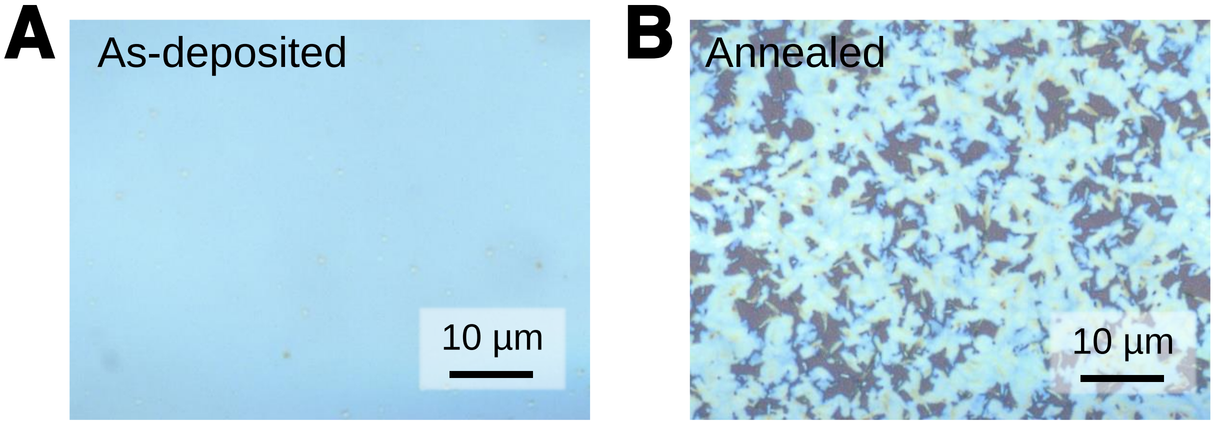

Fig. S10. Optical microscopy (OM) images of MoO_3 layer on ITO/glass. (A, B) OM images of MoO_3 layer (A) before thermal treatment and (B) after thermal treatment. The evolution of grain-like structures is observed after thermal treatment.

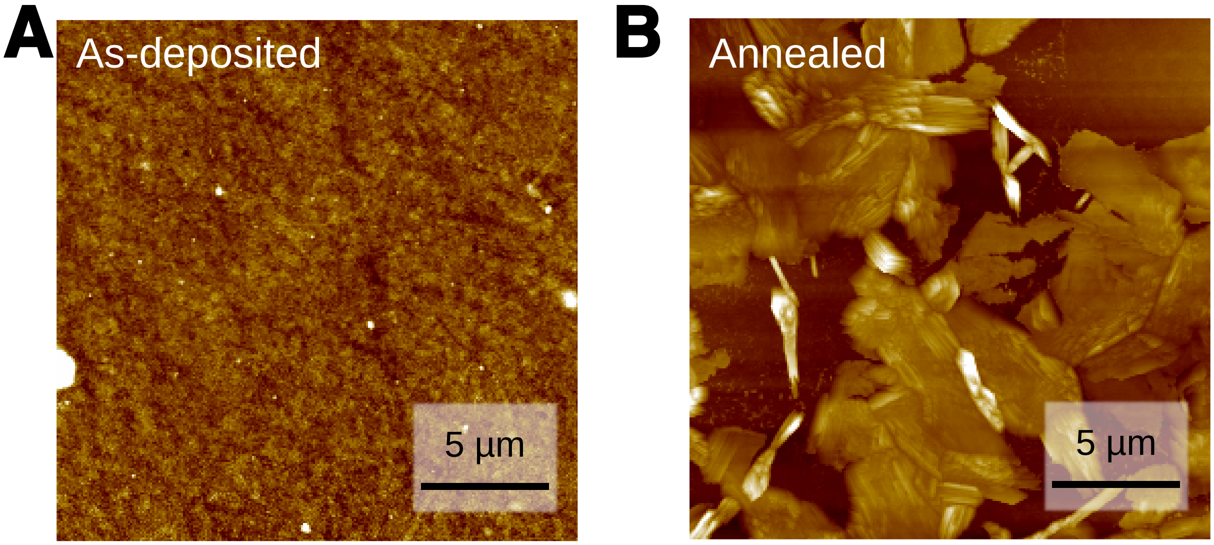

Fig. S11. Atomic force microscopy (AFM) images of MoO_3 layer on ITO/glass before and after the thermal treatment. (A, B) AFM images of MoO_3 layer (A) before thermal treatment and (B) after thermal treatment. The evolution of grain-like structures is observed after thermal treatment.

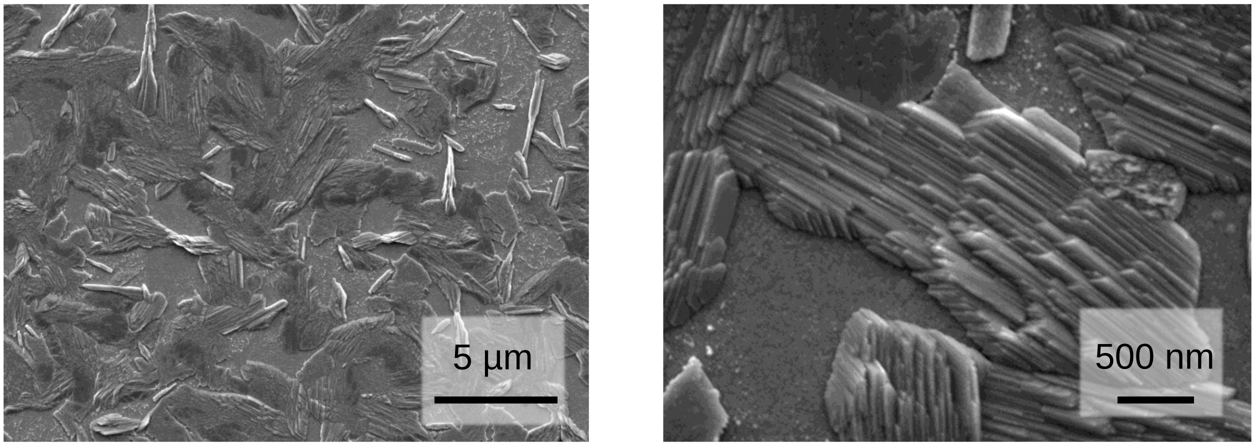

Fig. S12. FE-SEM images of MoO_3 layer after the thermal treatment. The images reveal 2D-layered structures that are intrinsic properties of α-MoO_3.

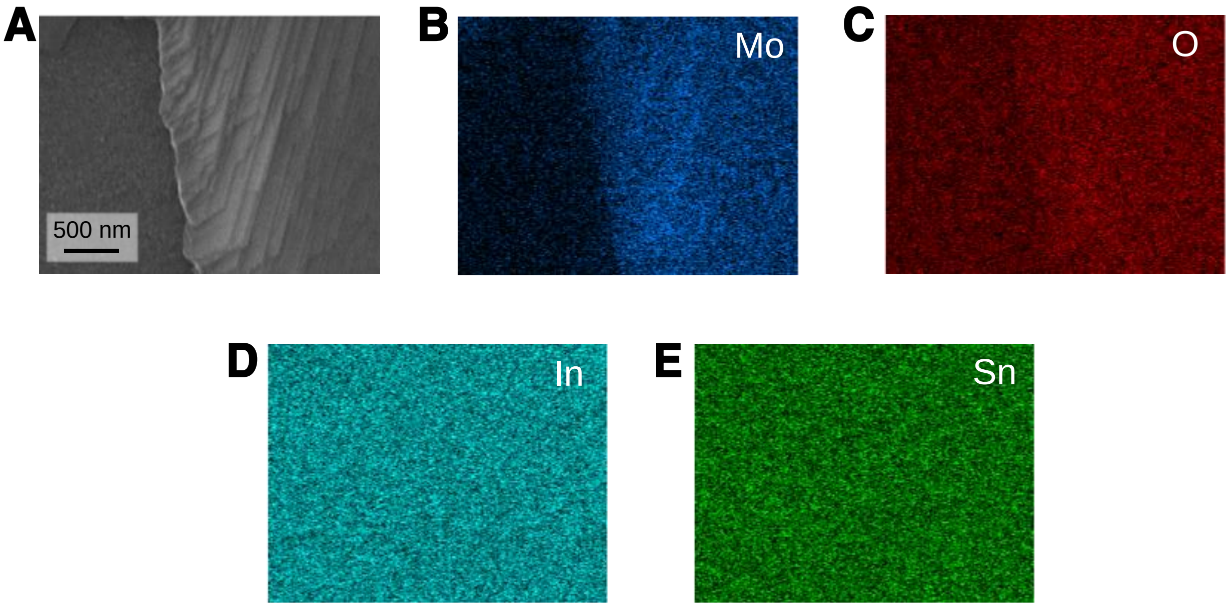

Fig. S13. Energy dispersive X-ray spectroscopy (EDS) mapping of the MoO_3 grain. (A) SEM image of a MoO_3 grain on ITO. **(B** to **E)** EDS mapping of (B) Mo, (C) O, (D) In, and (E) Sn elements of the MoO_3 grain on ITO.

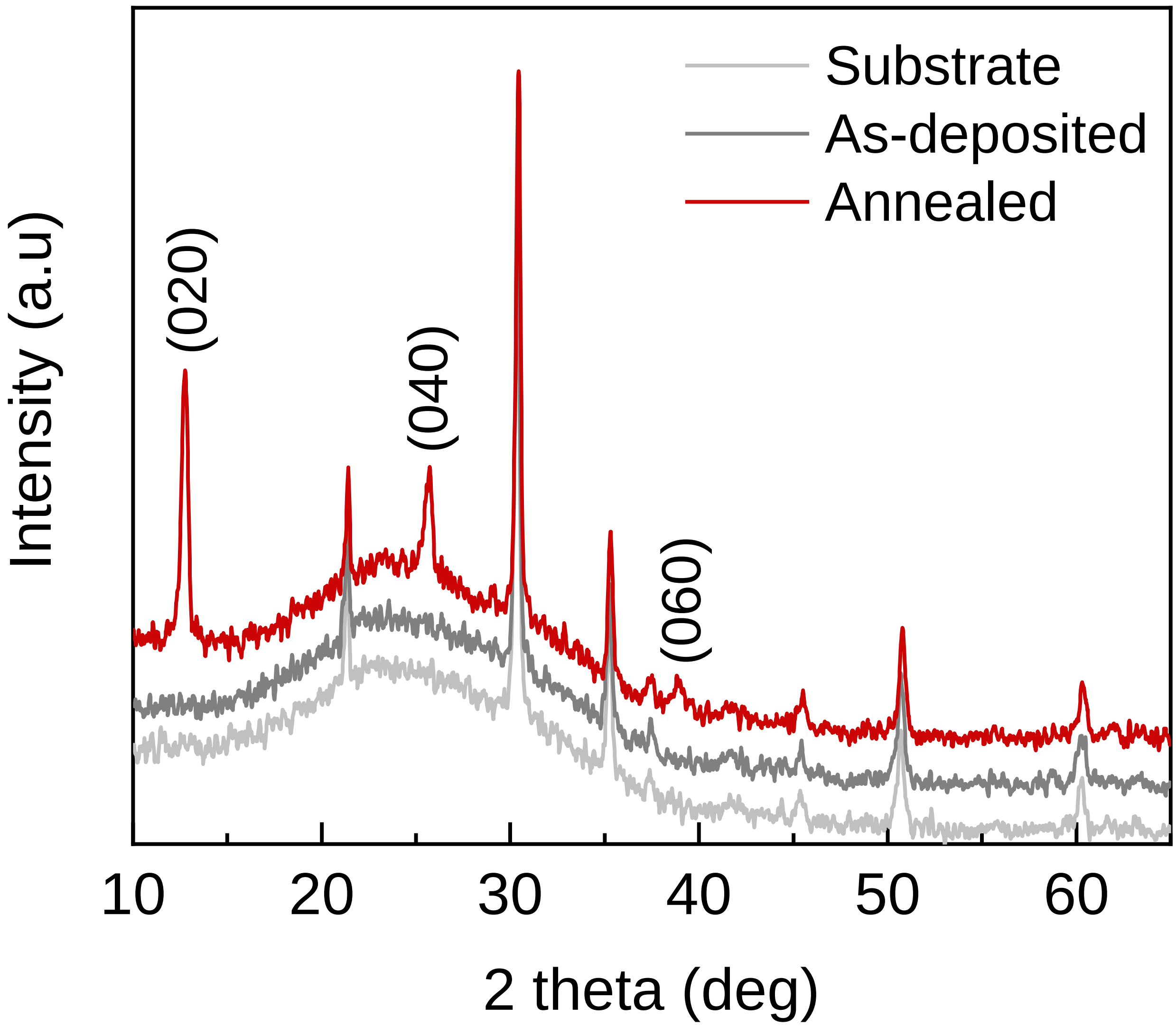

Fig. S14. X-ray diffraction patterns of MoO_3 before and after thermal treatment. The (020), (040), and (060) peaks, which are characteristic of the α-MoO_3 structure, emerge after the annealing process.

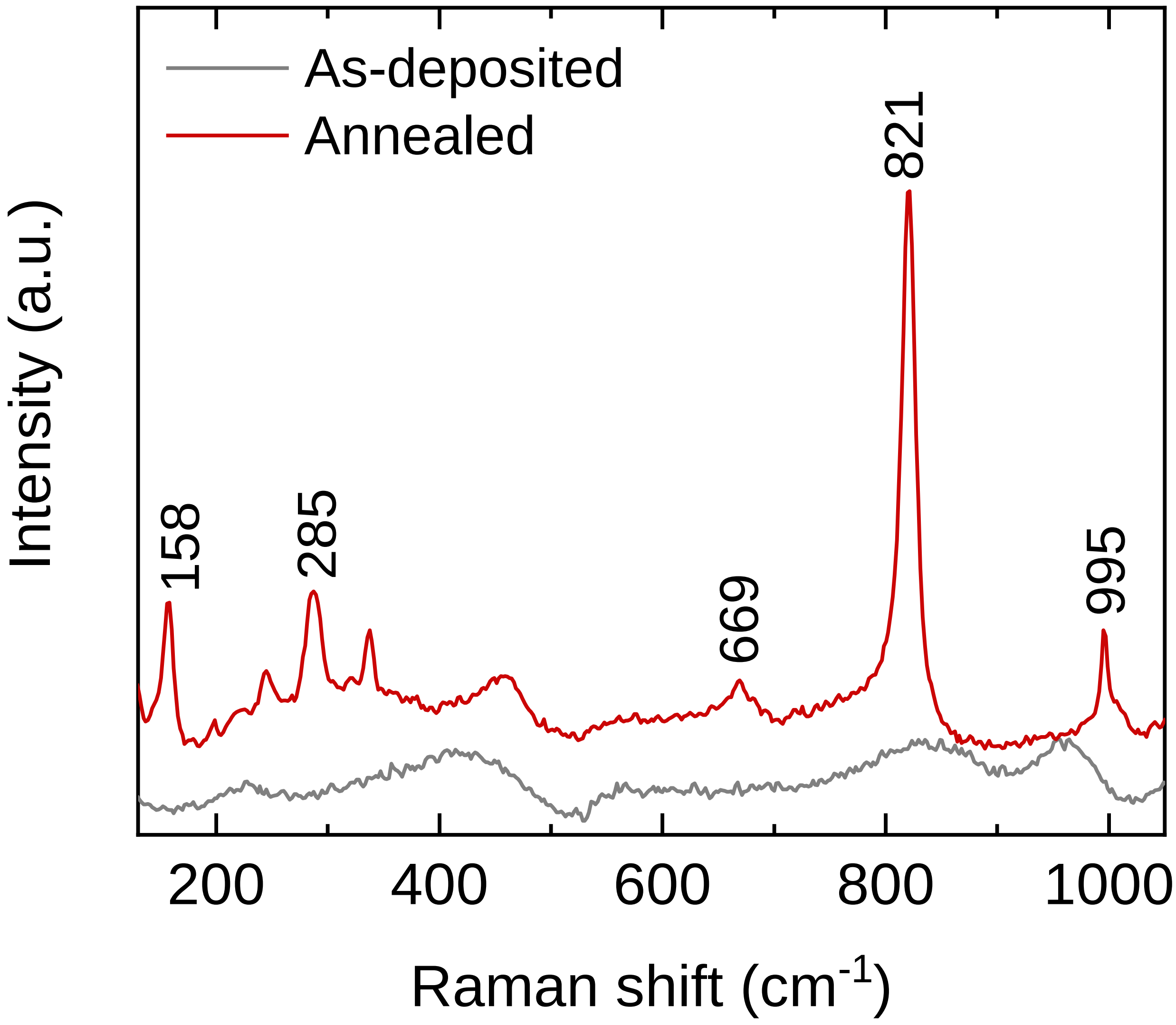

Fig. S15. Raman spectroscopy images of MoO_3 before and after thermal treatment. Characteristic peaks of α-MoO_3 structure emerge after thermal treatment.

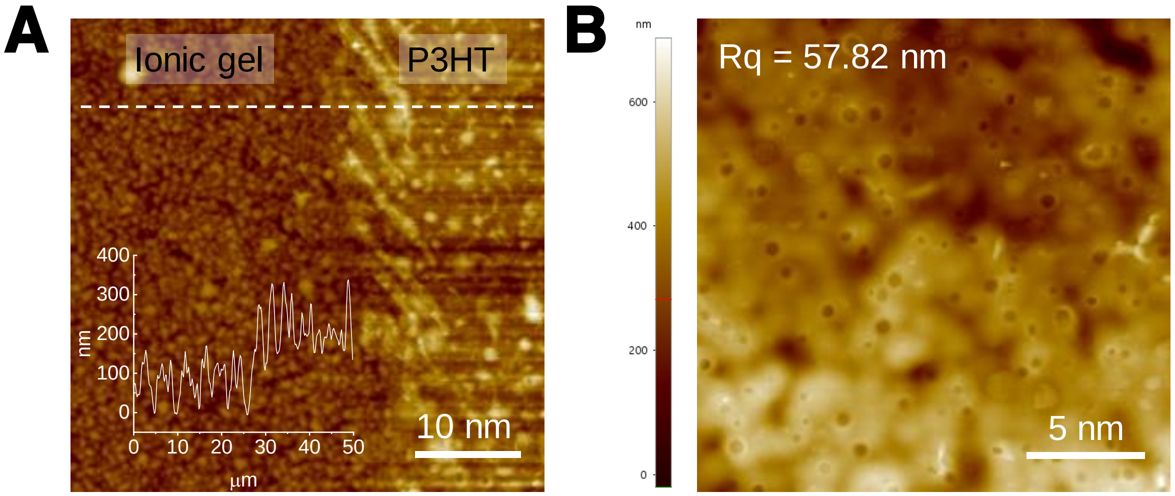

Fig. S16. AFM images of P3HT/ionic gel. (A-B) (A) Thickness profile and (B) Height image morphology of P3HT film on LiTFSI/PVDF-HFP ionic gel. The P3HT film showed an average thickness of 120 nm and an Rq of 57.82 nm.

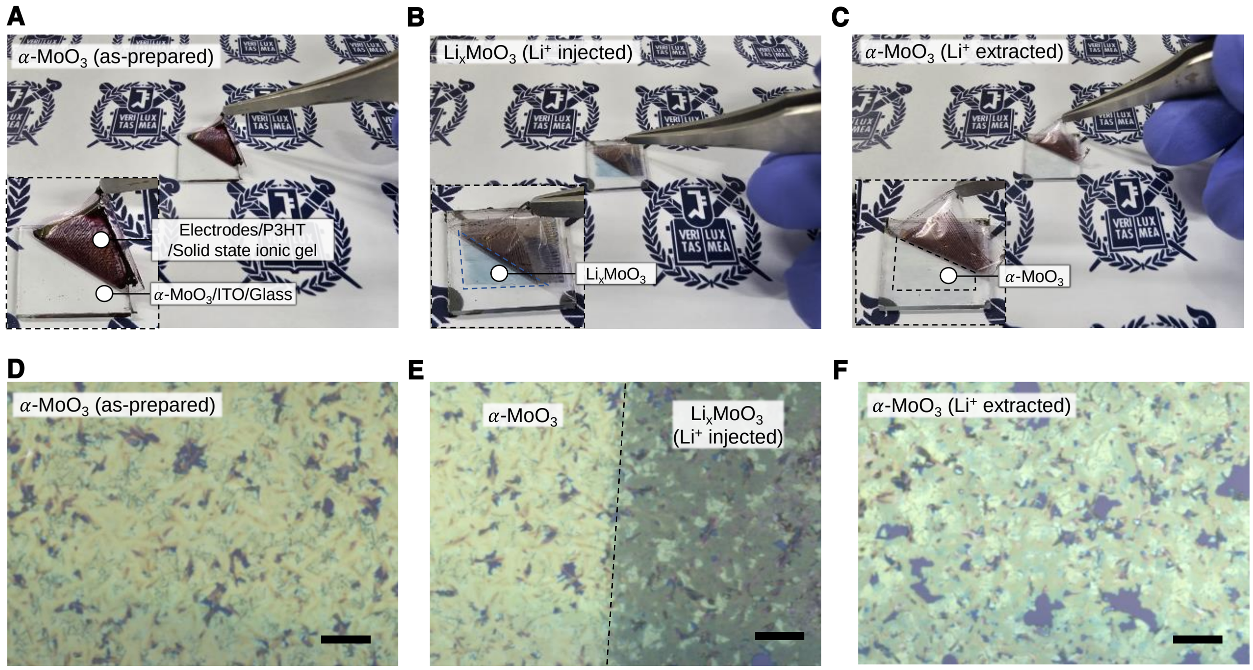

Fig. S17. Delamination processes for XPS and UV-Vis spectroscopy, and optical microscope images of three different MoO_3 layers. (A to **C),** Delamination processes of (A) as-prepared, (B) Li^+ injected, and (C) Li^+ extracted MOSD. **(D** to **F)**, Optical microscope images of (D) as-prepared, (E) Li^+ injected, and (F) Li^+ extracted MoO_3 layer. Scale bar is 10 μm.

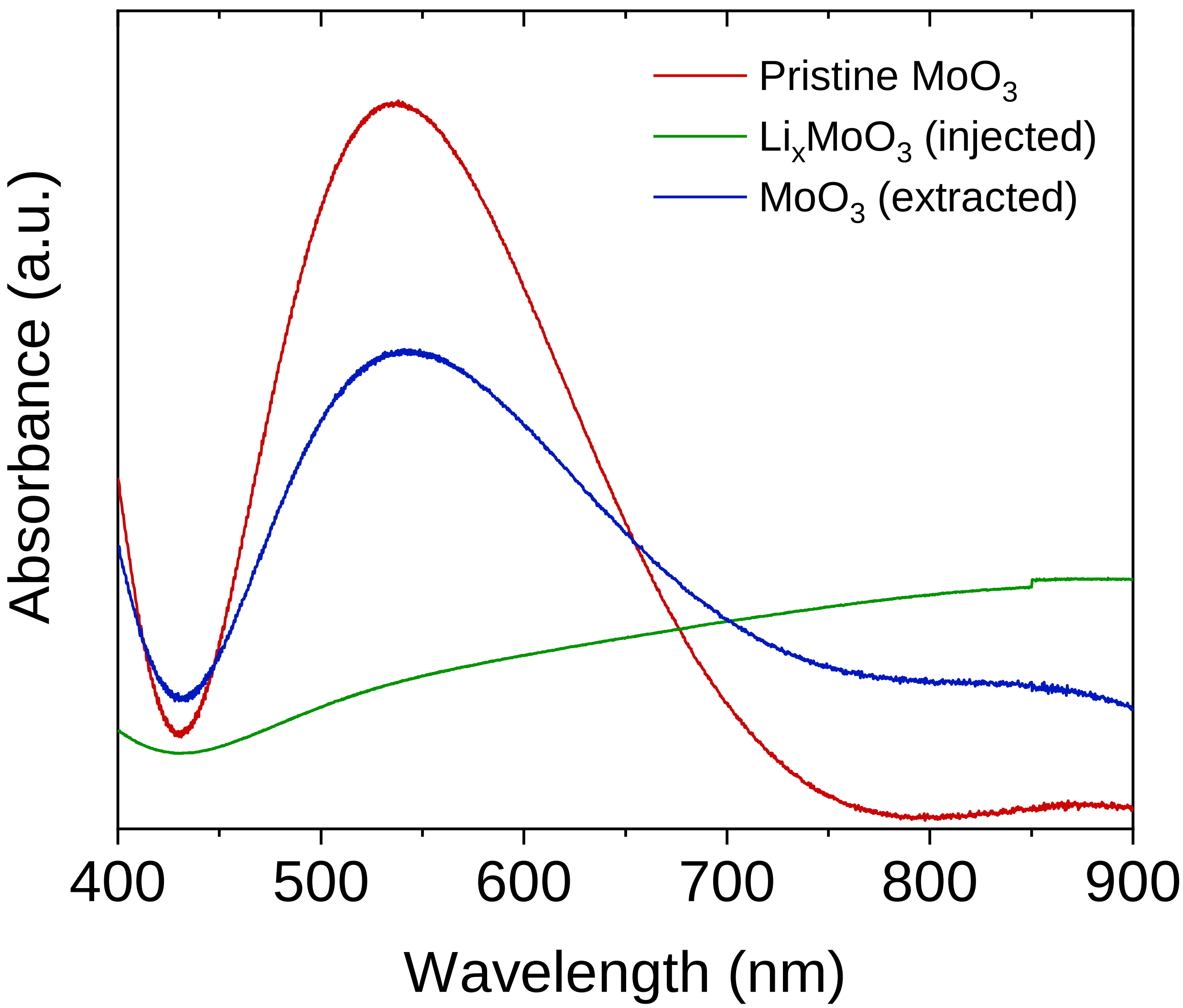

Fig. S18. UV-Visible light spectroscopy of α-MoO_3. Doped Li_xMoO_3 (green) absorbs higher wavelengths compared with as-prepared pristine MoO_3 (red), whereas it absorbs similar wavelengths compared with pristine MoO_3 after de-doping (blue).

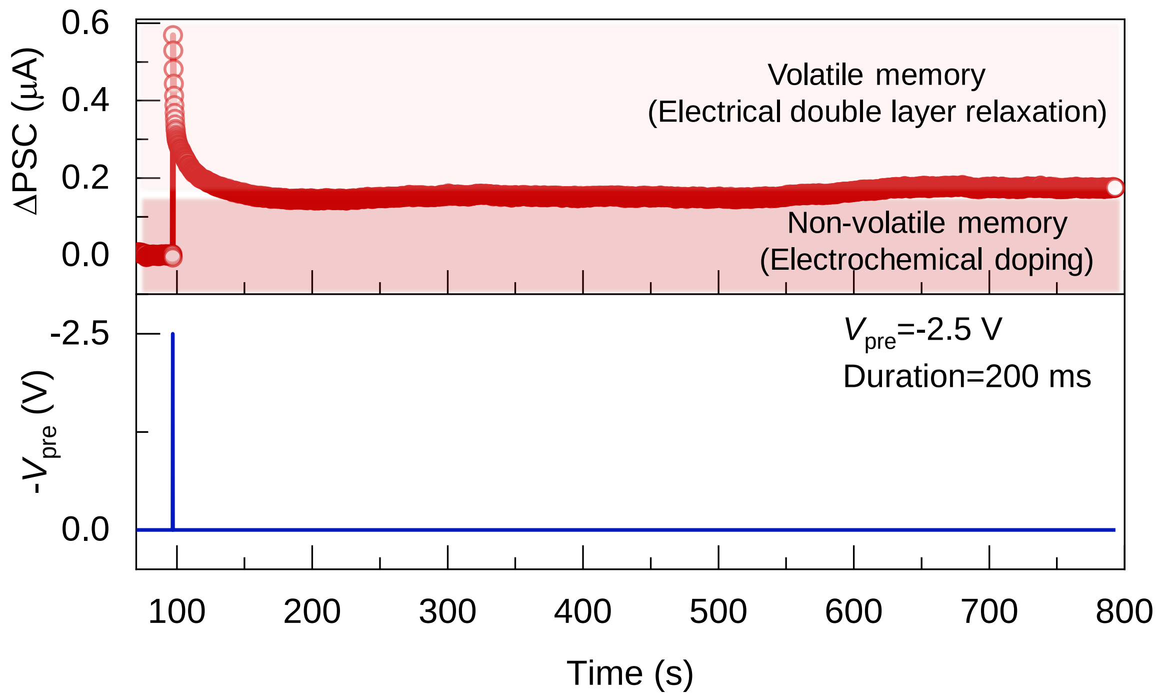

Fig. S19. EPSC response of MOSD. The memory lasts for about 700 s.

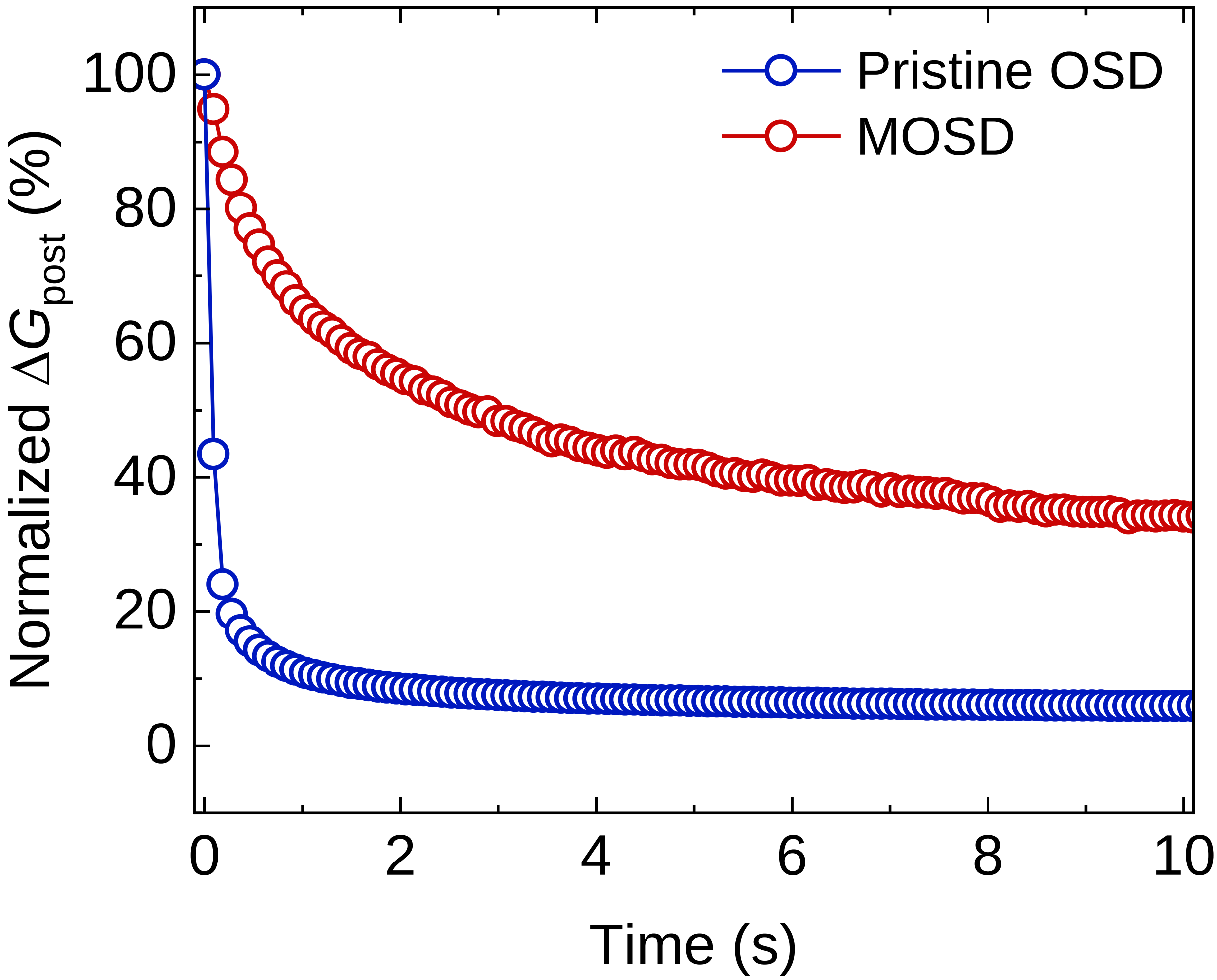

Fig. S20. Normalized post-synaptic conductance (G_{post}) changes as a function of time. The altered G_{post} of pristine OSD dramatically decreases and is erased within 2 s, whereas the change in MOSD is relatively well preserved until 10 s. For EPSC response, a V_{pre} pulse of -2.5 V was applied with a duration of 200 ms.

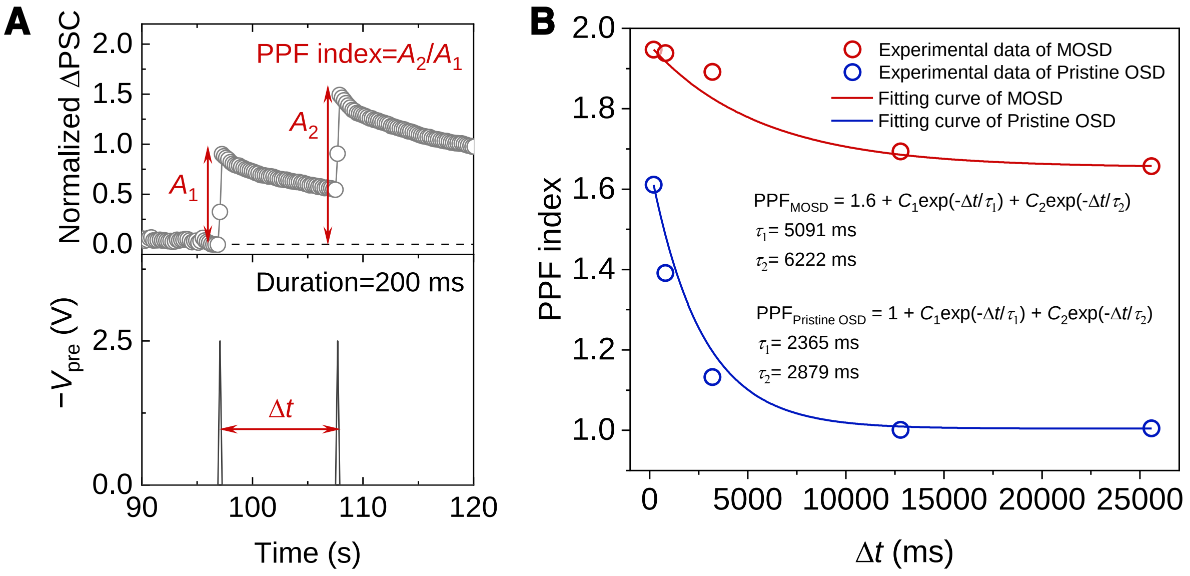

Fig. S21. Plot of paired-pulse facilitation (PPF) index of pristine OSD and MOSD. (A) Definition of PPF index. A_1 and A_2 are normalized magnitudes of first and second PSC peaks. Δt is the time interval between two presynaptic pulses (B) PPF index as a function of pulse interval time for pristine OSD and MOSD. For PPF responses, V_{pre} pulses of −2.5 V was applied with a duration of 200 ms and a given interval.

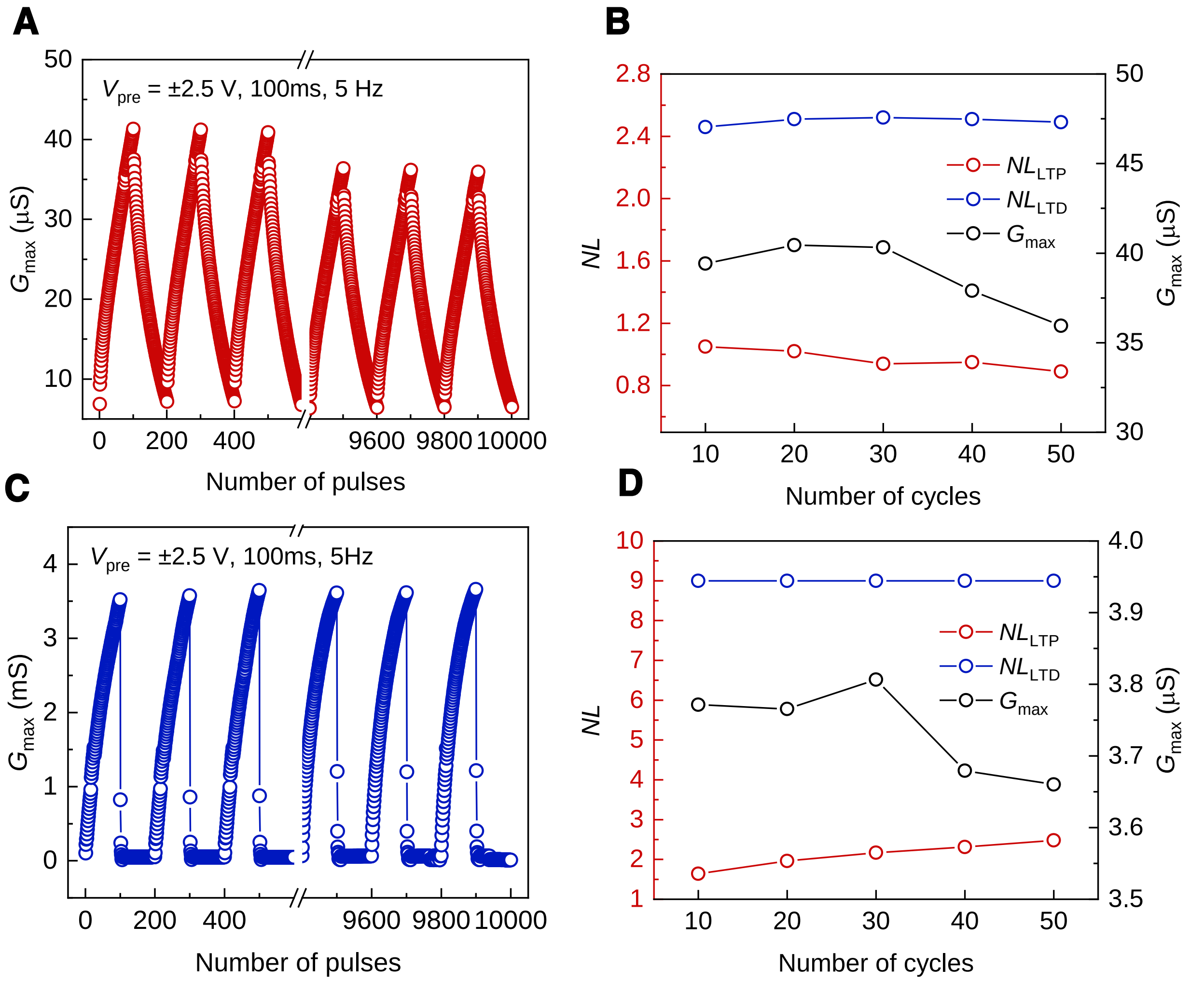

Fig. S22. Cycle-to-cycle variations (CCV) of long-term potentiation and depression (LTP/D) Properties in pristine OSD and MOSD. (A) 50 cycles of LTP/D in MOSD. **(B)** CCV of maximum conductance (G_{max}) and non-linearity (*NL*) during 50 cycles of LTP/D in MOSD. **(C)** 50 cycles of LTP/D in pristine OSD. **(D)** CCV of maximum conductance (G_{max}) and non-linearity (*NL*) during 50 cycles of LTP/D in pristine OSD. For each LTP/D cycle, 100 consecutive potentiation pulses (V_{pre} = -2.5 V, 5 Hz) followed by 100 consecutive depression pulses (V_{pre} = +2.5 V, 5 Hz) were applied to the pre-synaptic electrode.

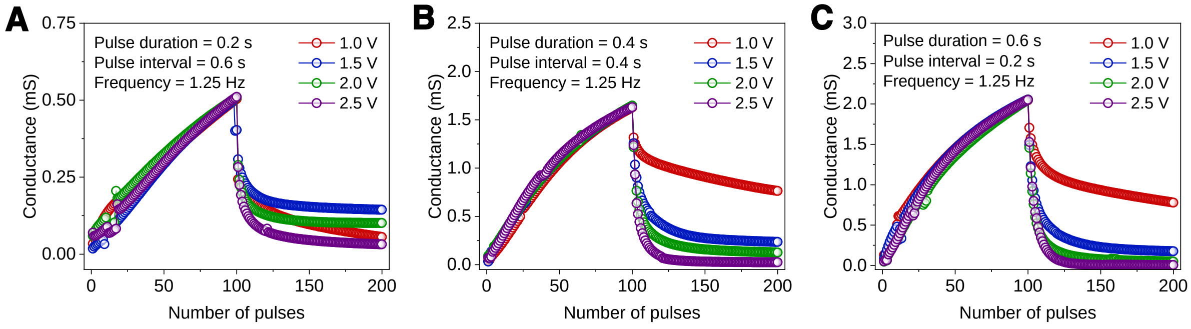

Fig. S23. LTP/D cycle of pristine OSD with various depression voltages. (A to C) Pulse durations were varied with (A) 0.2 s, (B) 0.4 s, and (C) 0.6 s. For all cases, potentiation voltages and frequencies were fixed with −2.5 V and 1.25 Hz.

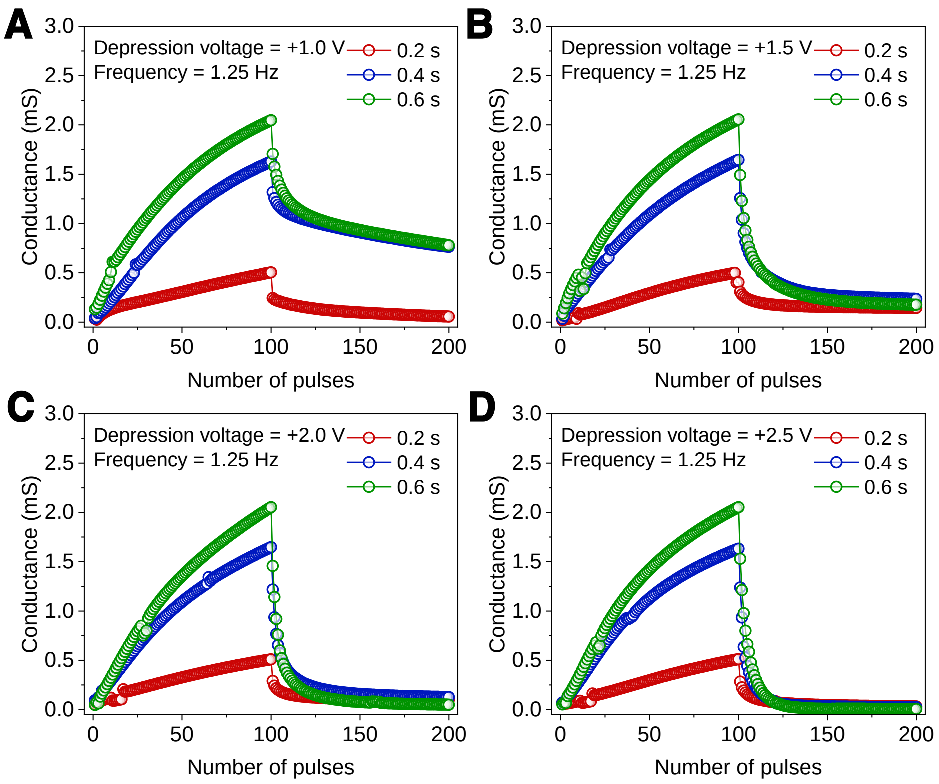

Fig. S24. LTP/D cycle of pristine OSD with various pulse duration. (A to D) Depression voltages were varied with (A) +1.0 V, (B) +1.5 V, (C) +2.0 V, and (D) +2.5 V. For all cases, potentiation voltages and frequencies were fixed with −2.5 V and 1.25 Hz

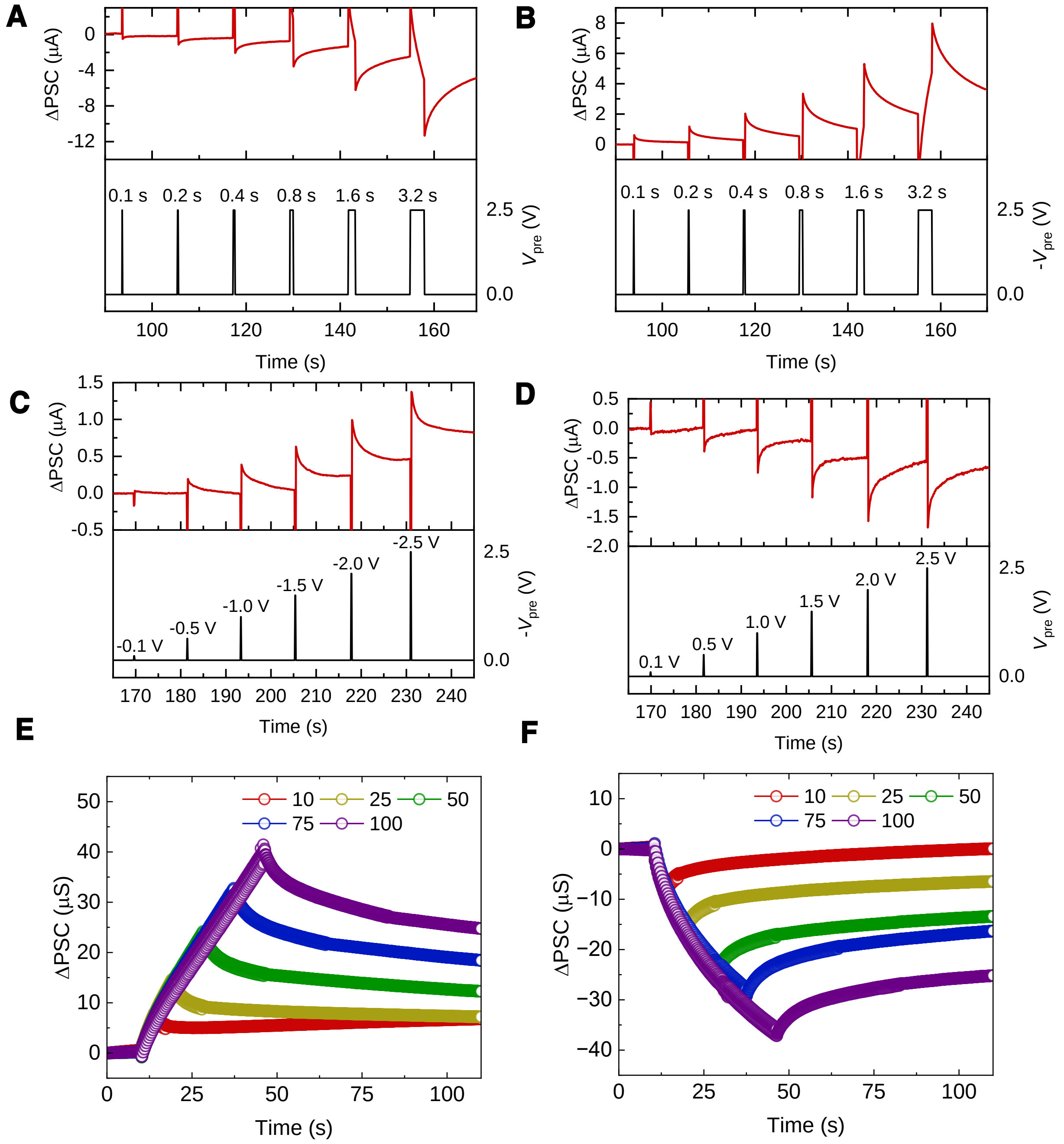

Fig. S25. Synaptic plasticity of MOSD. (A, B) Spike-duration-dependent (A) EPSC and (B) IPSC. For EPSC and IPSC response, V_{pre} pulses of ±2.5 V was applied with various durations. **(C, D)** Spike-voltage-dependent (C) EPSC and (D) IPSC. For EPSC and IPSC response, V_{pre} pulses of various voltages was applied with a duration of 200 ms. **(E, F)** Spike-number-dependent (E) EPSC and (F) IPSC. For EPSC and IPSC response, V_{pre} pulses of ±2.5 V was applied with duration of 100 ms and frequency of 5 Hz.

Table S1. G_{max} and NL values extracted from LTP/D cycles in pristine OSD.

Cycle no.	G_{max} (mS)	NL_{LTP}	NL_{LTD}
10	3.77	1.64	9 <
20	3.77	1.96	9 <
30	3.81	2.17	9 <
40	3.68	2.31	9 <
50	3.66	2.48	9 <

Table S2. G_{max} and *NL* values extracted from LTP/D cycles in MOSD.

Cycle no.	G_{max} (μS)	NL_{LTP}	NL_{LTD}
10	39.41	1.05	2.46
20	40.45	1.02	2.51
30	40.32	0.94	2.52
40	37.89	0.95	2.51
50	35.95	0.89	2.49

Table S3. G_{max}**,** NL**, and** NS_{eff} **values extracted from LTP/D cycles in MOSD.**

Pulse no.	G_{max} (μS)	NL_{LTP}	NL_{LTD}	$NS_{eff,LTP}$	$NS_{eff,LTD}$
25	18.31	0.41	2.36	25	25
50	25.74	1.14	2.66	50	50
75	32.27	1.13	2.74	75	75
100	36.90	1.00	2.78	99	100

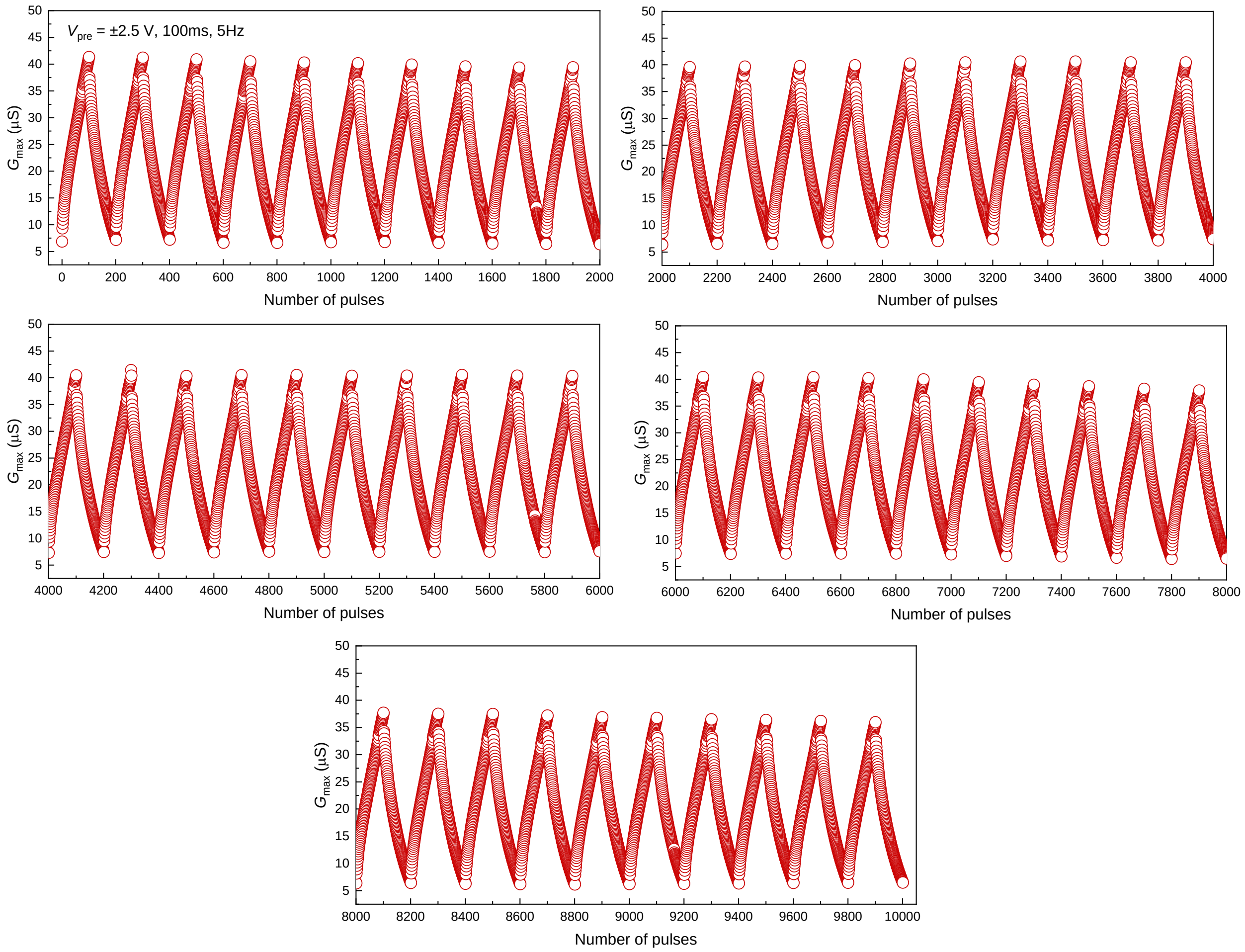

Fig. S26. First to 50th LTP/D cycles in MOSD.

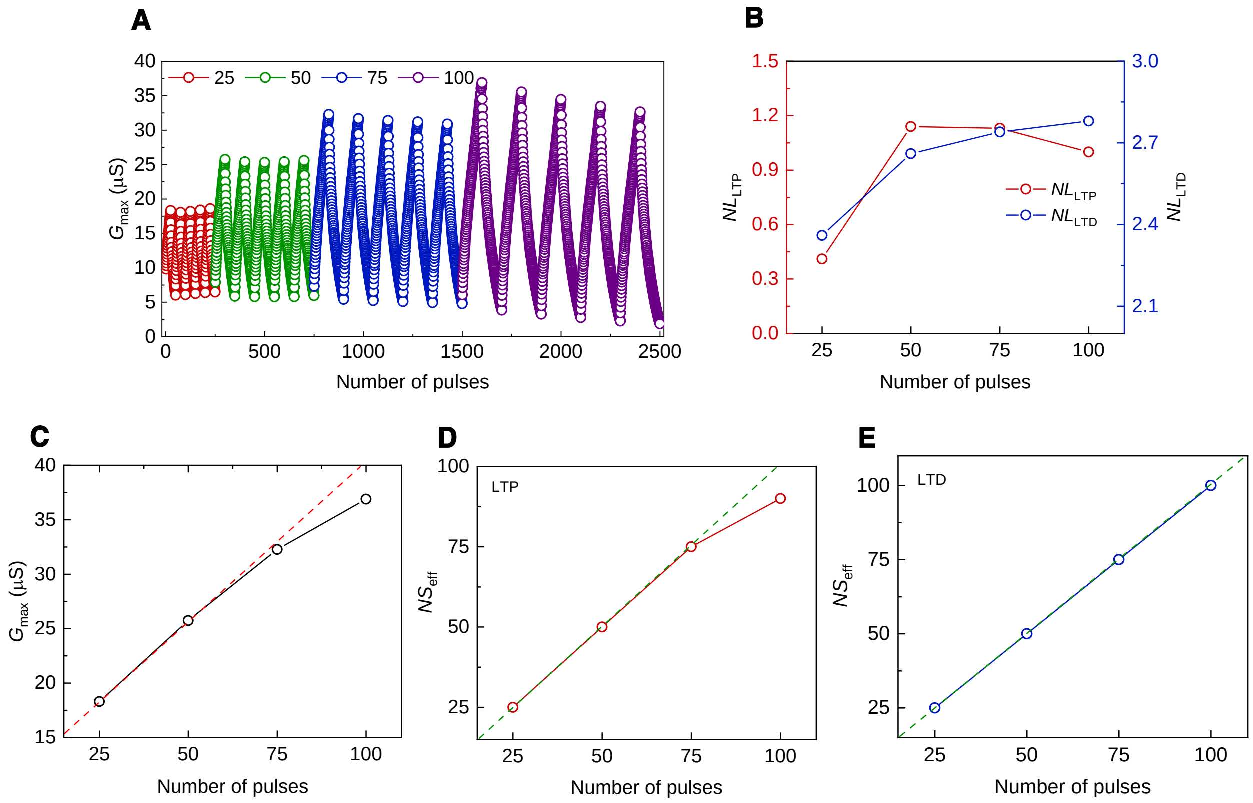

Fig. S27. *NL*, G_{max}, and effective number of states (NS_{eff}) characteristics of MOSD under various potentiation/depression pulse sets. (A) LTP/D characteristics of MOSD under various potentiation/depression pulse sets. For LTP/D responses, V_{pre} pulses of ±2.5 V were applied given times each with a duration of 100 ms and a frequency of 5 Hz. **(B)** Plots of *NL* as functions of pulse number for a single cycle. **(C)** Plots of G_{max} as functions of pulse number for a single cycle. **(D, E)** Plots of NS_{eff} as functions of pulse number for a single cycle of (D) LTP and (E) LTD. States having ΔG above the noise range (0.5% of $G_{max} - G_{min}$) were defined as the effective states.

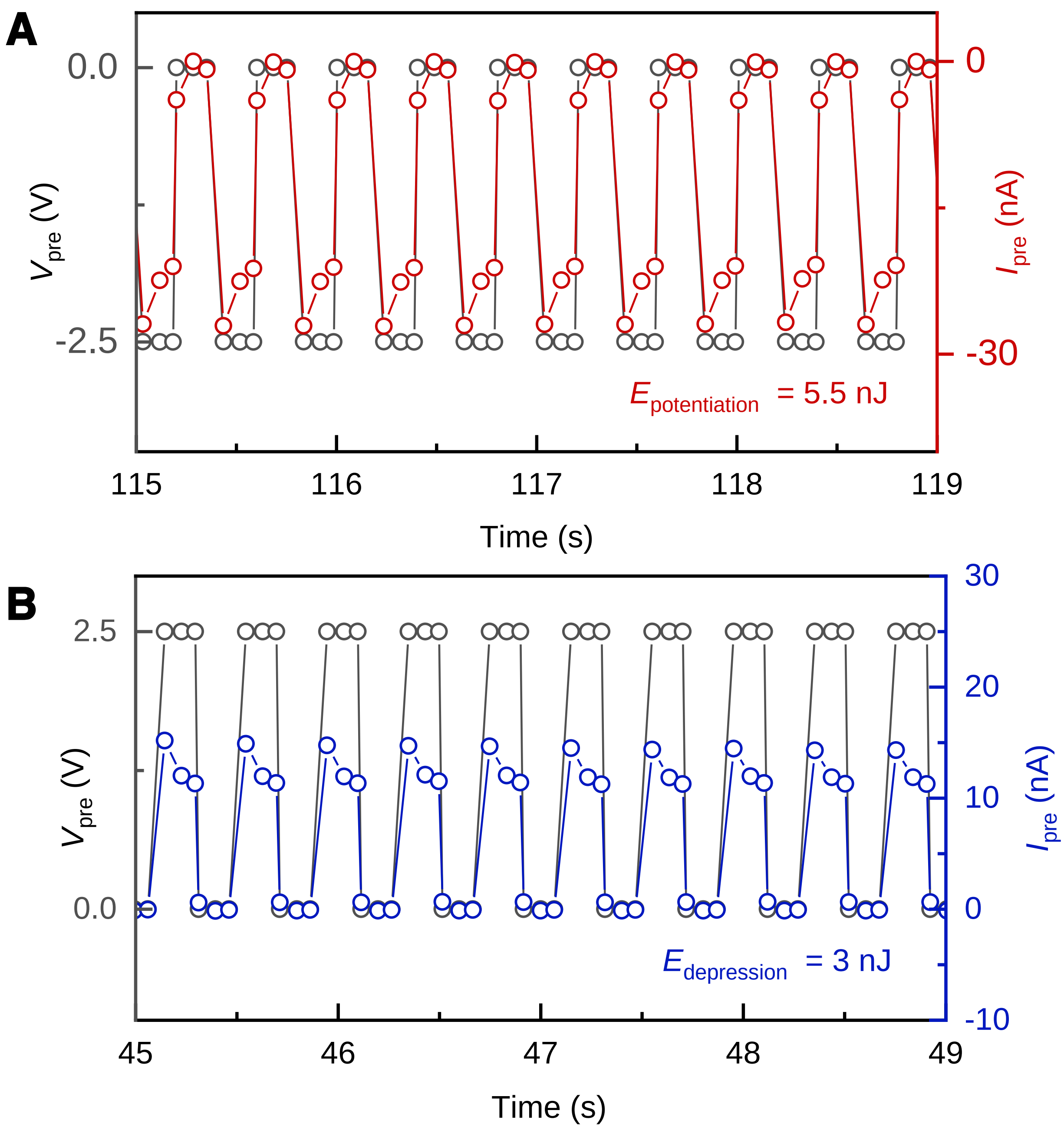

Fig. S28. Energy consumption in MOSD. (A, B) Pre-synaptic currents (I_{pre}) measured when (A) potentiation and (B) depression pulses were applied to the pre-synaptic electrode. The energy for potentiation and depression pulses were calculated using the equation: $E = V_{pre} \times I_{pre} \times t$, where V_{pre} and I_{pre} represent the voltage bias and current of pre-synaptic electrode, and the t is the pulse width of the V_{pre}.

Supplementary Text 3. Training and inference simulation using artificial neural networks

We conducted training and inference simulations using theoretically constructed artificial neural networks (ANNs). The ANNs consisted of input, hidden, and output neurons, as well as synapses connecting these neurons. The numbers of input and output neurons depend on the dimensions of the input and output data. For example, in the case of the Modified National Institute of Standards and Technology (MNIST) digit patterns, which are 20 × 20 images, the input layer consists of 400 neurons, whereas the output layer comprises 10 neurons, corresponding to digits 0 through 9 (**Fig. S29**). The network includes 400 × 200 × 10 synapses connecting the neurons.

Adjacent neurons interact with each other during training and inference to process input information and learn from feedback. The strength of these interactions, known as synaptic weights, is modulated by the change in synaptic device conductance. We calculated the synaptic weight as $W = G^{+} - G^{-}$, where G^{+} and G^{-} are conductance of potentiation and depression synapse, respectively. Values corresponding to each region of the MNIST digit patterns were fed into the input neuron layer. After values from the 400 regions were applied to the input layer, post-synaptic currents ($I = \sum V \times W$) were transmitted to the pre-synapses of neurons in the hidden layer. Similarly, post-synaptic currents were generated and converted into an output value (f) through a sigmoid activation function. The output value was then compared with the corresponding label value, which represented the correct answer (ranging from 0 to 9). Finally, the synaptic weights were updated via backpropagation to minimize the difference between the output and label values.

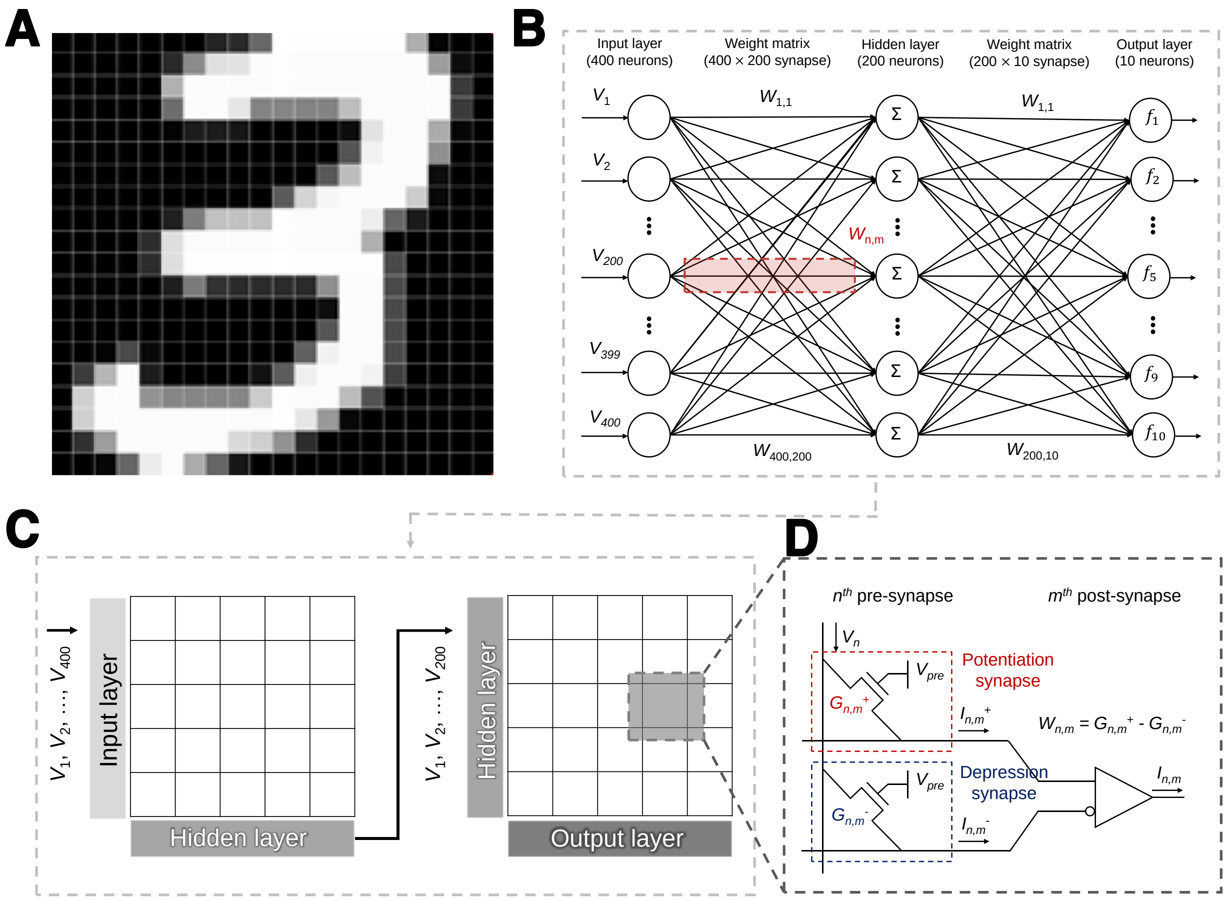

Fig. S29. MNIST dataset recognition task. (A) MNIST dataset which are 20 × 20 images. **(B)** Artificial Neural Network (ANN) consisting of an input layer, a hidden layer, and an output layer. **(C)** Hardware Neural Network (HW-NN) incorporating MOSD for fully connected ANN layers. **(D)** Circuit diagram of HW-NN.

Supplementary Text 4. Operating mechanism of the artificial olfactory system

The odorant selectivity is controlled by tight packing interactions between an odorant and a hOR. The hOR has a wide opening between TM5 and TM6, through which the hydrophobic tail of the odorant accesses the transmembrane pocket of hOR. The odorant binds to hOR with specific ionic and hydrogen-bonding interactions, and nonspecific hydrophobic interactions that rely on shape complementary with the aliphatic potion of the odorant (*75*). Upon odorant binding, substantial inward and outward movements of the extracellular and intracellular parts of TM6 occur, respectively. These conformational changes are stabilized by the interactions between the odorant and binding pocket (*76*). Although the molecular mechanism for signal transduction between hORs and sensing devices is still unclear, it has been reported that ligand binding induces a charge reorganization within the hOR structure (*77*), causes a decrease in the impedance and facilitates charge transfer through the hOR (*78*). Therefore, it could be hypothesized that the conformational change of hORs induces a charge reorganization within the hOR and its electrical property changes affect the sensing signal.

The charge transfer generated at the hOR NDs can induce a p-type doping effect on the graphene, altering the resistance of the OSN device in response to specific odorants. This signal is then conveyed to the pre-synaptic electrode through a thin gold wire. Subsequently, the lithiation (or de-lithiation) of $\boldsymbol{\alpha}$-MoO_3 occurs, along with the ionic doping (or de-doping) of the P3HT layer. These events lead to changes in post-synapse conductance.

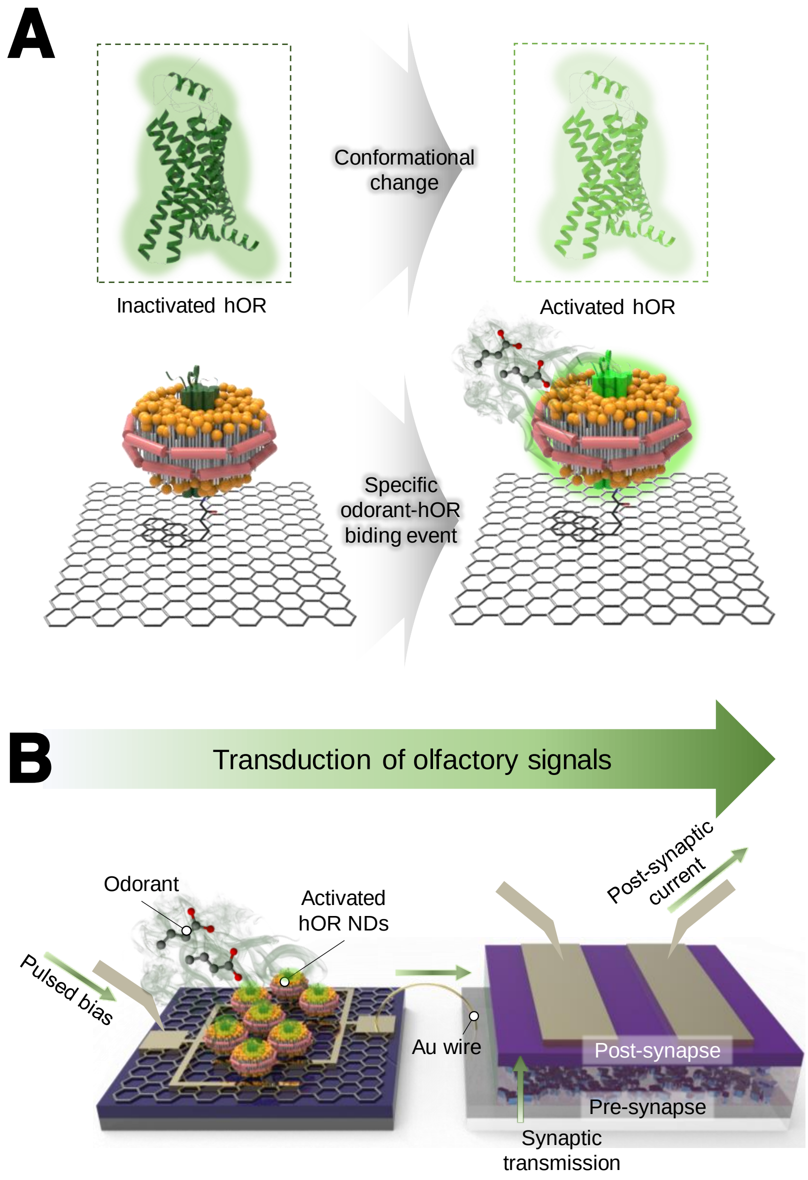

Fig. S30. Schematic illustration of the sensing mechanism of the artificial olfactory system. **(A)** Conformational change of hOR triggered by odorant-hOR binding events. **(B)** Signal transduction in AOS.

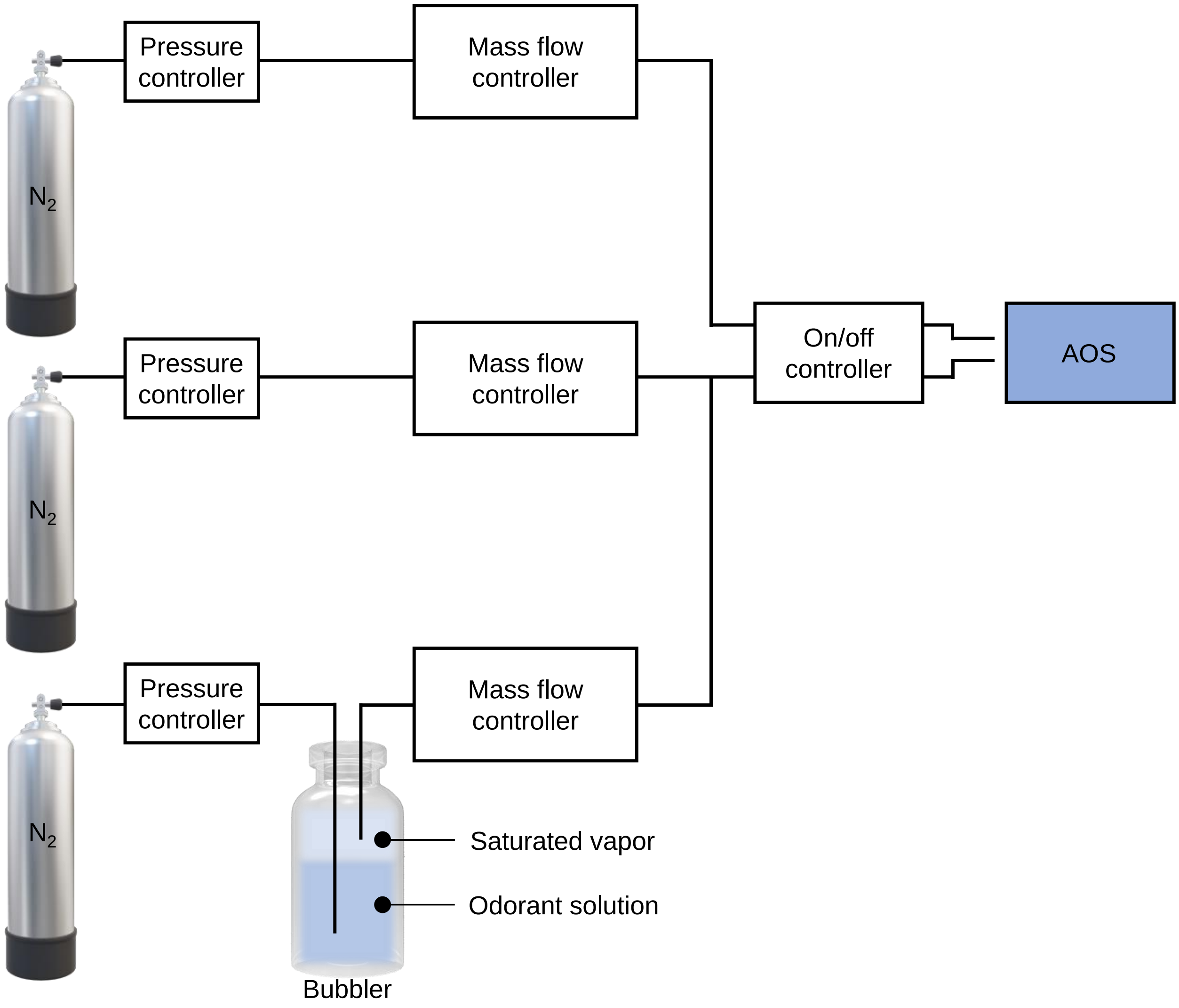

Fig. S31. Schematic diagram of gas generation system for releasing odorant molecules into the gas phase. The concentration of short-chain fatty acids (SCFAs) in the gas phase is regulated by controlling the mass flow of the nitrogen carrier gas. Vaporized SCFAs are generated by passing nitrogen gas through liquid SCFAs at various temperatures. The on/off controller allows gas flow to transition from the pure nitrogen source to the evaporated scent compound, while maintaining the same pressure and flow rate.

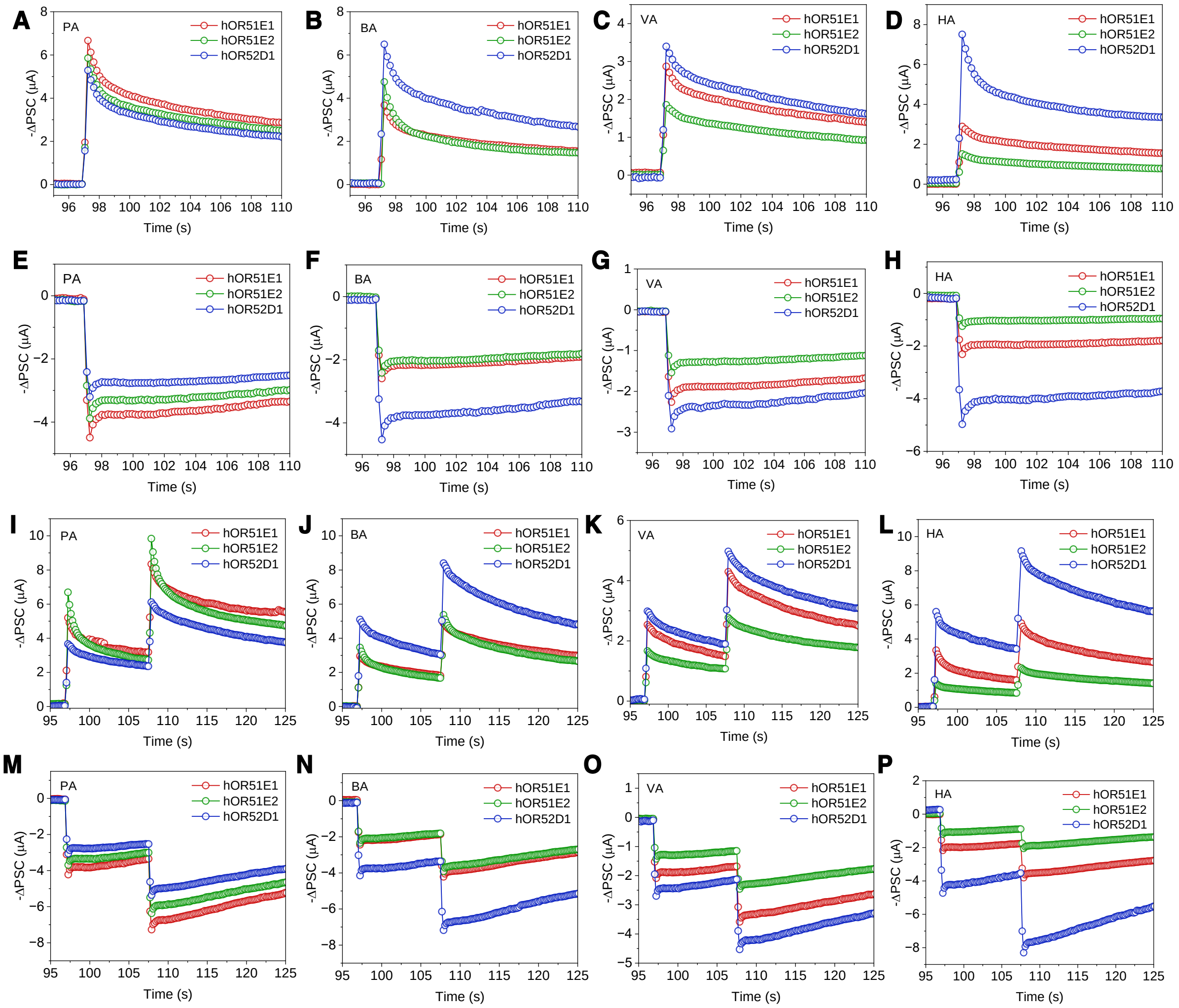

Fig. S32. Synaptic behaviors of AOSs in response to 3 ppm of SCFAs. (A to D) EPSC responses of AOSs (V_{pre} = −.5 V, duration = 200 ms). **(E to H)** IPSC responses of AOSs (V_{pre} = +2.5 V, duration = 200 ms). **(I to L)** PPF responses of AOSs (V_{pre} = −2.5 V, duration = 200 ms, Δt = 10 s). **(M to P)** PPD responses of AOSs (V_{pre} = +2.5 V, duration = 200 ms, Δt = 10 s).

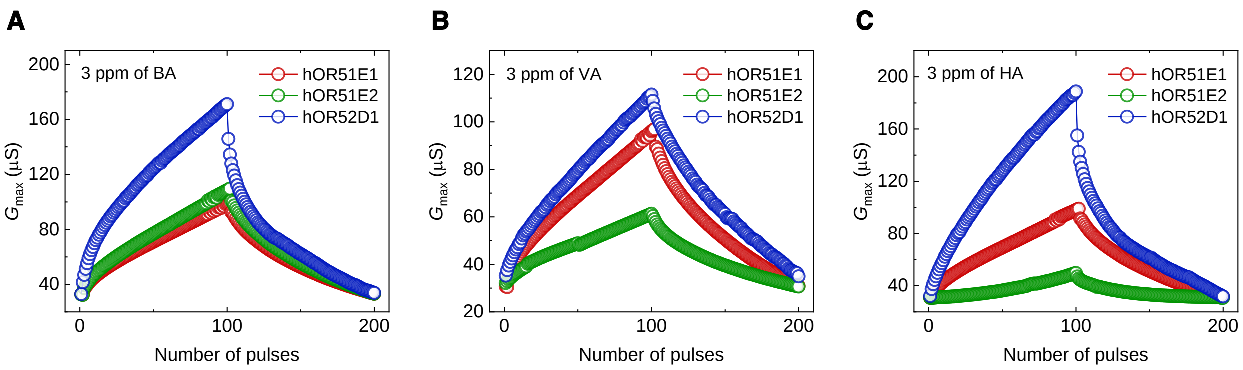

Fig. S33. LTP/D characteristics of AOS with various hOR NDs according to exposed short-chain fatty acids (SCFAs). (A) Butyric acid (BA). **(B)** Valeric acid (VA). **(C)** Hexanoic acid (HA). For LTP/D responses, V_{pre} pulses of ±2.5 V were applied 100 times each with a duration of 100 ms and a frequency of 5 Hz.

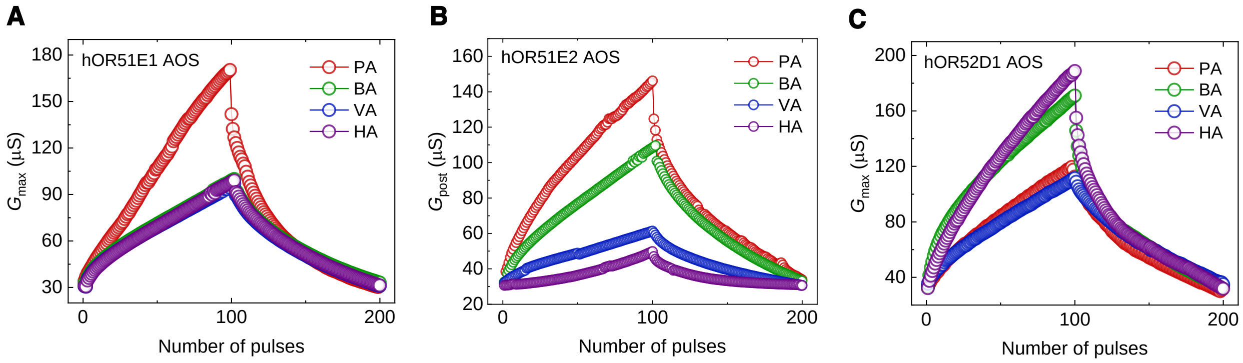

Fig. S34. LTP/D characteristics of AOS with various hOR NDs according to exposed SCFAs. (A to **C)** LTP/D characteristics of (A) hOR51E1, (B) hOR51E2, (C) hOR52D1 AOSs. Concentrations of all SCFAs are controlled at 3 ppm. For LTP/D responses, V_{pre} pulses of ±2.5 V were applied 100 times each with a duration of 100 ms and a frequency of 5 Hz.

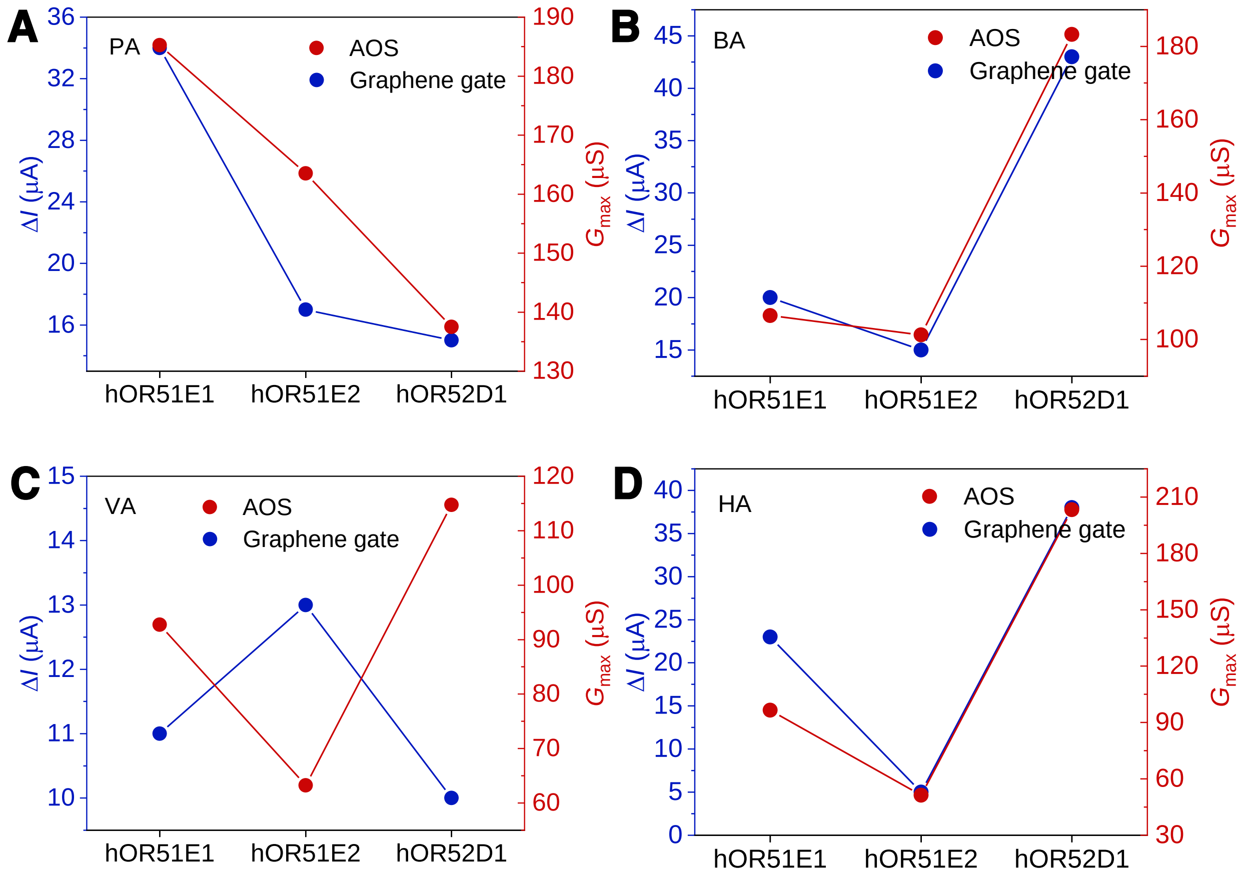

Fig. S35. Comparison of the responses of AOSs and graphene extended-gate for 3 ppm of SCFAs. (A to D) Comparison of responsivities of AOSs and graphene gates in response to 3 ppm of (A) PA, (B) BA, (C) VA, (D) HA.

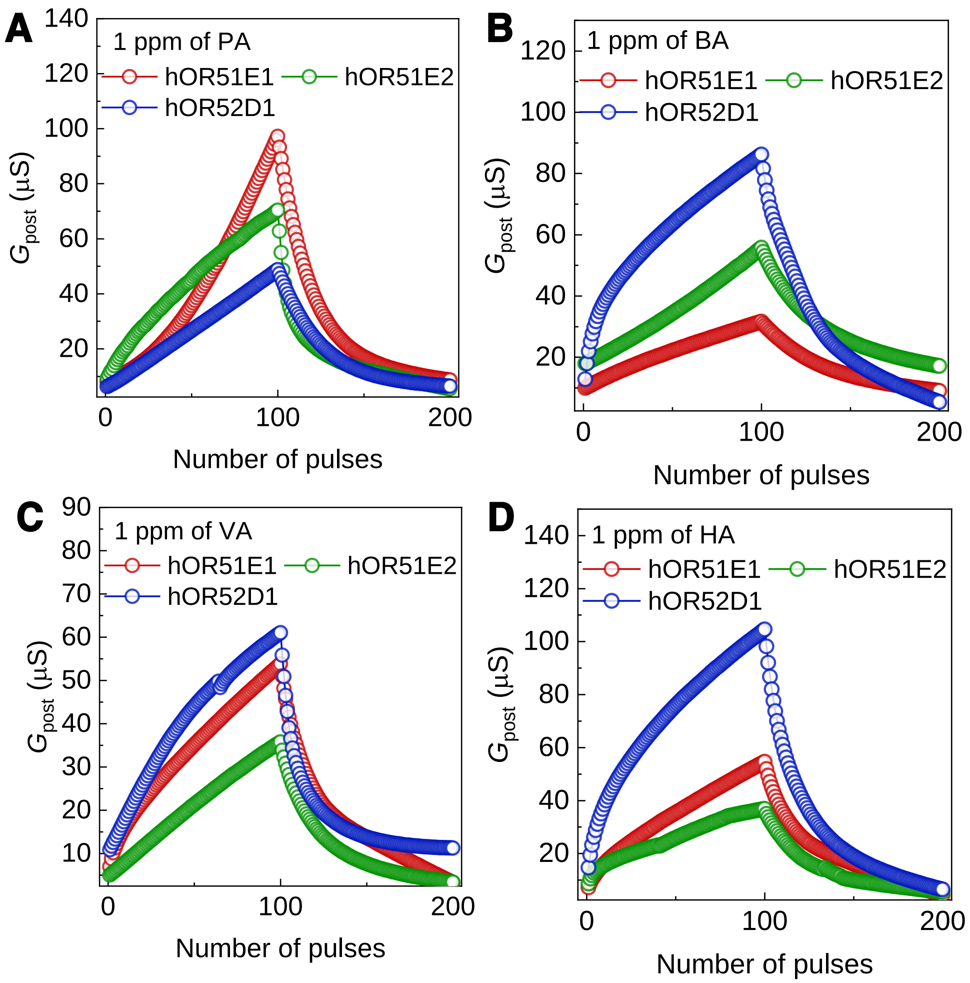

Fig. S36. LTP/D characteristics of AOS with various hOR NDs according to exposed 1 ppm of short-chain fatty acids (SCFAs). (A to D) 1 ppm of (A) propionic acid (PA), (B) butyric acid (BA), (C) valeric acid (VA), and (D) hexanoic acid (HA). For LTP/D responses, V_{pre} pulses of ±2.5 V were applied 100 times each with a duration of 100 ms and a frequency of 5 Hz.

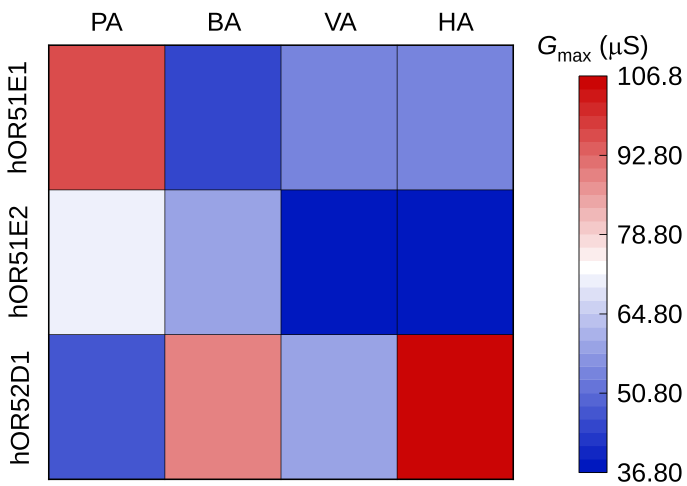

Fig. S37. Patterned conductance signals of AOSs in response to 1 ppm of various SCFAs.

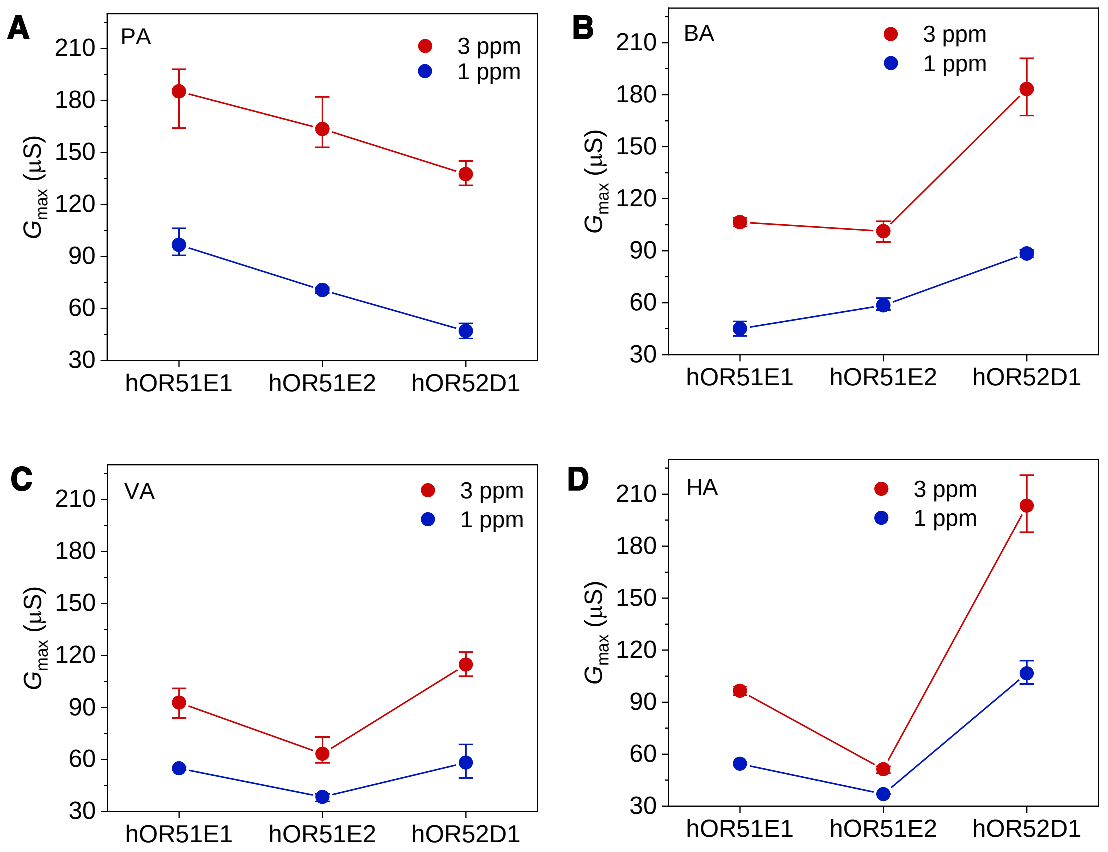

Fig. S38. Comparison of G_{max} responses of AOSs in response to 1 ppm and 3 ppm of SCFAs.

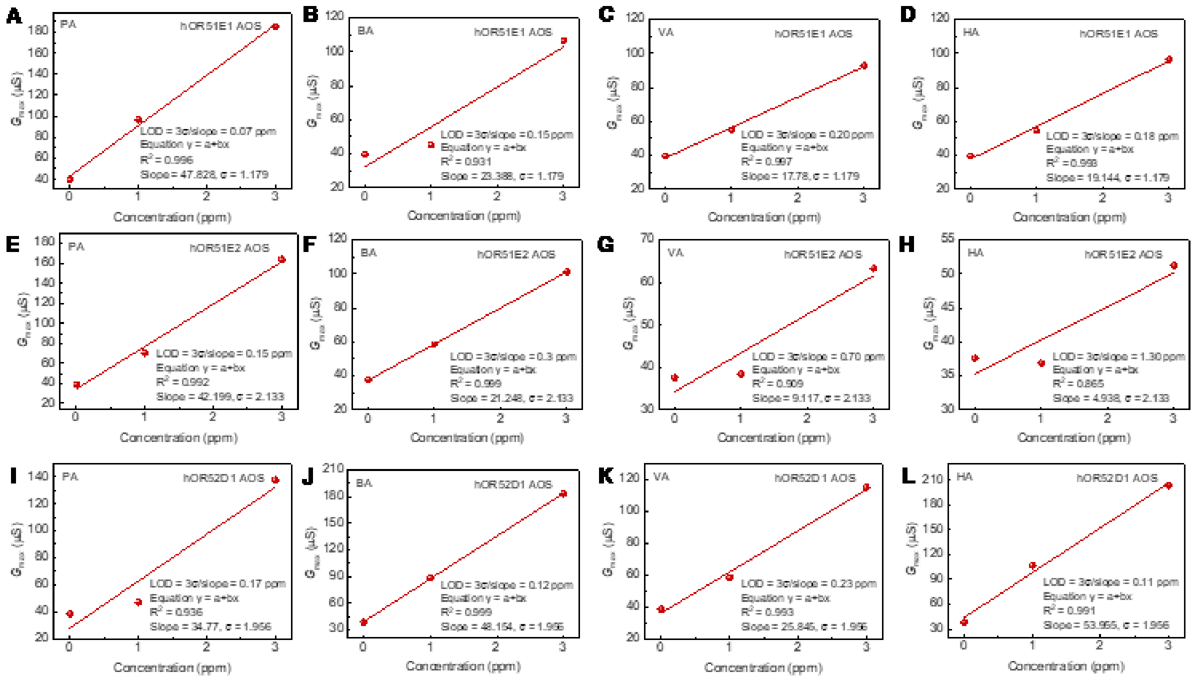

Fig. S39. Plot for the determination of detection limit for SCFAs. (A to **D)** Calibration curves based on the G_{max} values of AOS in response to (A) PA, (B) BA, (C) VA, (D) HA. **(E** to **H)** Calibration curves based on the G_{max} values of AOS in response to (E) PA, (F) BA, (G) VA, (H) HA. **(I** to **L)** Calibration curves based on the G_{max} values of AOS in response to (I) PA, (J) BA, (K) VA, (L) HA.

Table S4. Detection limit (ppm) of AOSs in response to SCFAs.

Odorant	hOR51E1 AOS	hOR51E2 AOS	hOR52D1 AOS
PA	0.07	0.15	0.17
BA	0.15	0.30	0.12
VA	0.20	0.70	0.23
HA	0.18	1.30	0.11

Table S5. Original dataset for principal component analysis. Each value means the G_{max} extracted from the LTP/D cycle.

	PA (C_3)			BA (C_4)			VA (C_5)			HA (C_6)		
	hOR51E1 AOS (μS)	hOR51E2 AOS (μS)	hOR52D1 AOS (μS)	hOR51E1 AOS (μS)	hOR51E2 AOS (μS)	hOR52D1 AOS (μS)	hOR51E1 AOS (μS)	hOR51E2 AOS (μS)	hOR52D1 AOS (μS)	hOR51E1 AOS (μS)	hOR51E2 AOS (μS)	hOR52D1 AOS (μS)
Device 1	164	153	134	107	107	168	84	60	108	97	53	188
Device 2	186	154	131	106	95	183	90	62	108	96	50	203
Device 3	193	182	145	104	100	181	96	58	121	94	50	201
Device 4	198	165	140	109	103	201	101	73	122	99	49	221

Table S6. Extracted eigenvectors of the two principal component (PC) analysis

	Coefficient of PC1	Coefficient of PC2
hOR51E1 AOS	0.66378	0.25183
hOR51E2 AOS	0.6676	0.22501
hOR52D1 AOS	-0.33719	0.94125

Supplementary Text 5. Molecular similarity calculation

To compare the odorant discrimination capability of the hOR-embedded AOS with the capability described in previous studies, we adopted a molecular similarity coefficient, which quantifies the similarity between two molecules. Among the available similarity coefficients, we selected the Tanimoto coefficient (Tc), which is widely used in cheminformatics and computational medicinal chemistry because of its ease of implementation and computational efficiency (*79*).

The Tanimoto coefficient between two molecules is defined as

$$Tc(A, B) = \frac{c}{a + b - c}$$

where a and b represent the number of structural features of molecules A and B, respectively, and c is the number of structural features shared by the two molecules. Tc ranges between 0 and 1; values near 1 indicate high similarity between the molecules and values near 0 indicate dissimilarity. The features used in the Tc calculation are based on molecular descriptors, known as molecular fingerprints.

We used the RDKit tool in Python to represent molecular fingerprints and calculate the Tc. First, we converted the molecular structures of odorants into simplified molecular-input line-entry system (SMILES) format, which is a notation for representing molecular structures with ASCII characters. Then, using the RDKit tool, we converted the SMILES into molecular fingerprints consisting of binary vector values. Finally, we calculated the Tc values between pairs of molecules using their fingerprints. Because a Tc cannot describe more than two molecules simultaneously, we calculated Tc values for all possible pairs and used the maximum Tc value from each study for comparison.

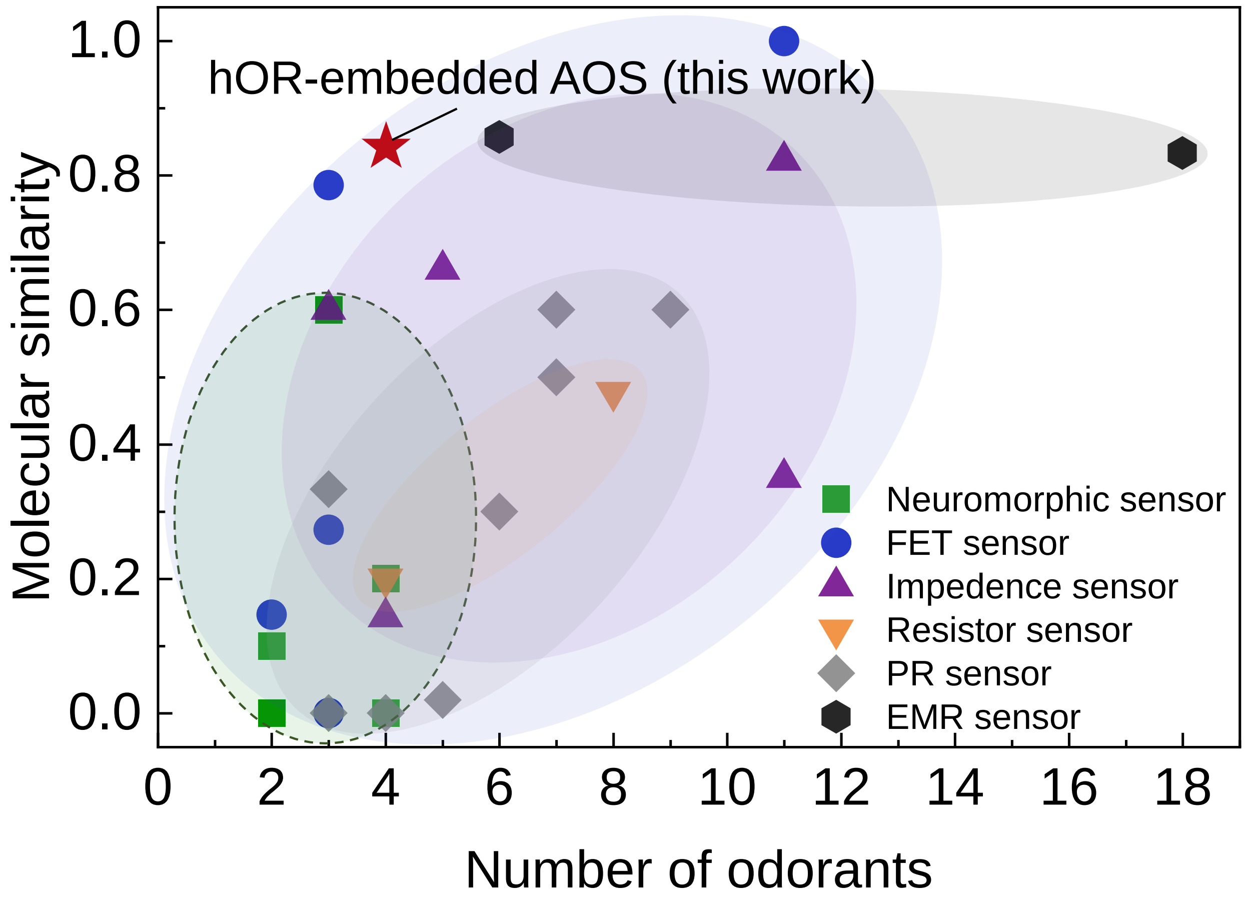

Fig. S40. Comparison of reported electronic gas sensors with our work regarding odorant number and molecular similarity.

Table S7. Comparison of reported neuromorphic gas sensors with our work regarding odorant number and molecular similarity.

Ref.	Number of odorants	Odorants	Pair	Tanimoto coefficient
This work	4	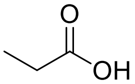 Propionic acid (PA)	PA, BA	0.733
			PA, VA	0.579
		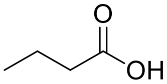 Butyric acid (BA)	PA, HA	0.478
		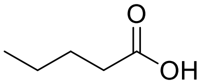 Valeric acid (VA)	BA, VA	0.789
		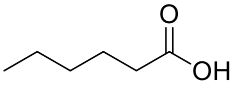 Hexanoic acid (HA)	BA, HA	0.652
			VA, HA	0.826
6	3	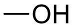 Methanol (MeOH)	MeOH, EtOH	0.333
		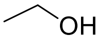 Ethanol (EtOH)	MeOH, PrOH	0.2
		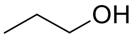 Propanol (PrOH)	EtOH, PrOH	0.6
7	2	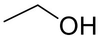 EtOH 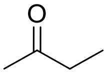 Methyl ethyl ketone (MEK)	EtOH, MEK	0.1
8	1	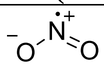 Nitrogen dioxide (NO_2)	N.A.	N.A.
9	1	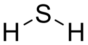 Hydrogen sulfide	N.A.	N.A.

Table S7. Continued

Ref.	Number of odorants	Odorants	Pair	Tanimoto coefficient
10	1	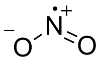 Nitrogen dioxide	N.A.	N.A.
11	1	O=O<br>Oxygen	N.A.	N.A.
12	4	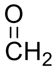 Formaldehyde (CH_2O)	CH_2O, EtOH	0
		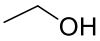 EtOH	CH_2O, acetone	0.2
		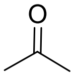 Acetone	CH_2O, toluene	0.026
		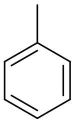 Toluene	EtOH, acetone	0.142
			EtOH, toluene	0
			Acetone, toluene	0.021
13	4	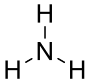 Ammonia (NH_3)	NH_3, CO	0
		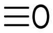 Carbon monoxide (CO)	NH_3, acetone	0
		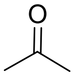 Acetone	NH_3, NO_2	0
		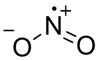 Nitrogen dioxide	CO, acetone	0
			CO, NO_2	0
			Acetone, NO_2	0

Table S7. Continued

Ref.	Number of odorants	Odorants	Pair	Tanimoto coefficient
14	3	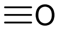<br>Carbon monoxide	CO, EtOH	0
		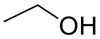<br>EtOH	CO, propane	0
		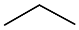<br>Propane	EtOH, propane	0.6
15	2	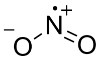<br>Nitrogen dioxide<br>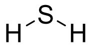<br>Hydrogen sulfide	NO_2, H_2S	0
16	2	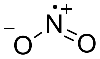<br>Nitrogen dioxide<br>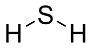<br>Hydrogen sulfide	NO_2, H_2S	0
17	1	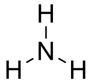<br>Ammonia (NH_3)	N.A.	N.A.
18	1	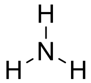<br>Ammonia (NH_3)	N.A.	N.A.

Table S8. Comparison of reported electronic gas sensors with our work regarding odorant number and molecular similarity.

Platform	Number of odorants	Maximum Tanimoto coefficient	Ref.
Neuromorphic sensor (*6*, *7*, *12*-*16*)	4	0.826	This work
	4	0.2	12
	4	0	13
	3	0.6	6
	3	0.6	14
	2	0.1	7
	2	0	15
	2	0	16
Transistor sensor (*19*-*24*)	11	1	20
	3	0.786	21
	3	0.273	22
	3	0	23
	3	0	24
	2	0.146	19
Resistor sensor (*25*, *26*)	8	0.478	25
	4	0.2	26
Impedance sensor (*77*-*81*)	11	0.822	77
	11	0.358	78
	5	0.661	79
	4	0.143	80
	3	0.6	81
Electromechanical sensor (EMR sensor) (*27*, *28*)	18	0.833	27
	6	0.857	28

Table S8. Continued

Platform	Number of odorants	Maximum Tanimoto coefficient	Ref.
Photonic sensor (PR sensor) (*29*-*36*)	9	0.6	29
	7	0.6	30
	7	0.5	31
	6	0.3	32
	5	0.077	33
	4	0	34
	3	0.333	35
	3	0	36

Supplementary Text 6. Tuning process of combinatorial odorant patterns

We refined combinatorial odorant patterns extracted from the maximum conductance (G_{max}) of the AOS, based on SCFAs, into more structured and specific patterns. As shown in **Fig. S41**, 1 × 3 original odorant patterns were transformed into 9 × 3 tuned odorant patterns, comprising binary values (0 or 1). Each hOR pixel from the original pattern activated one of the nine pixels in the tuned pattern, depending on the G_{max} value of that hOR pixel (**Fig. S42**). For example, in the case of a PA pattern, the G_{max} value of hOR51E1 was 170.3 μS, activating the 3rd region. The activation conditions are provided in **Table S9**. The detailed data tuning process is illustrated in **Fig. S43**. Through pattern splitting, activation, and flattening processes, the original odorant patterns were entered into the input layer in the form of tuned patterns. The tuned odorant patterns and their quantities are listed in **Tables S10-13**.

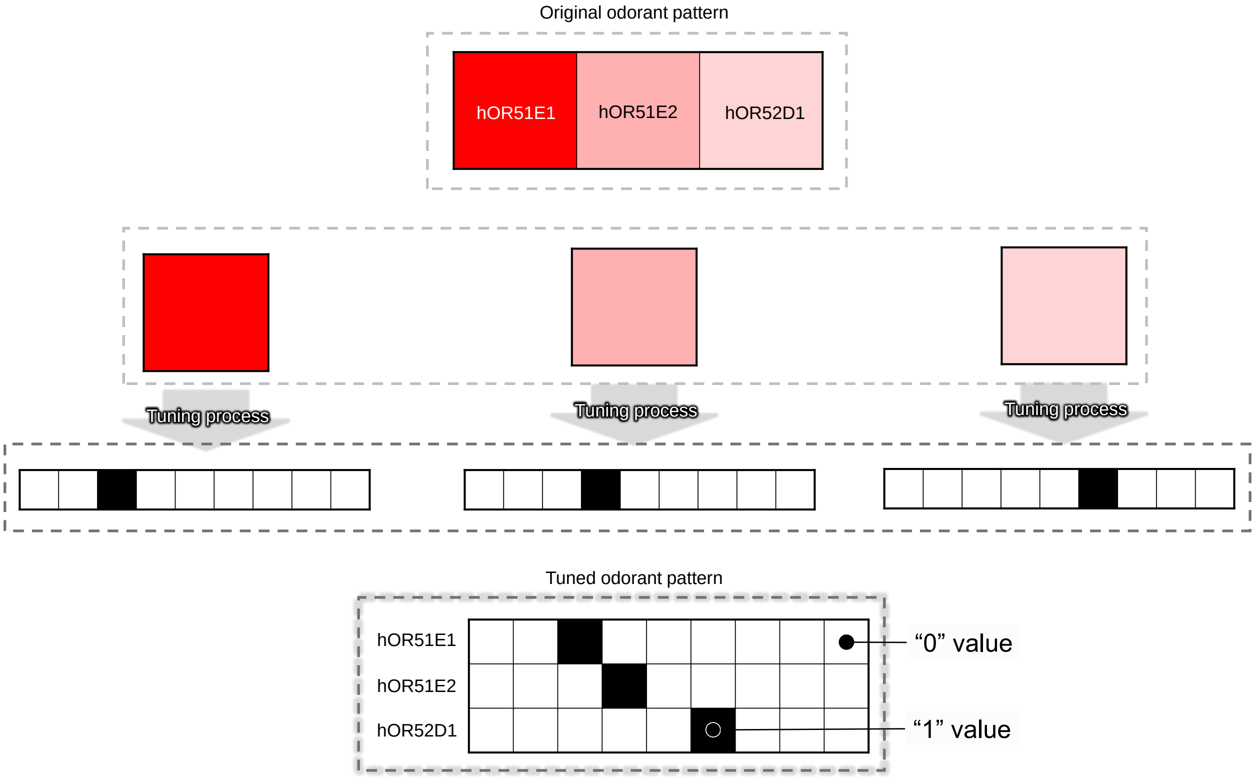

Fig. S41. Overall tuning process for combinatorial odorant patterns.

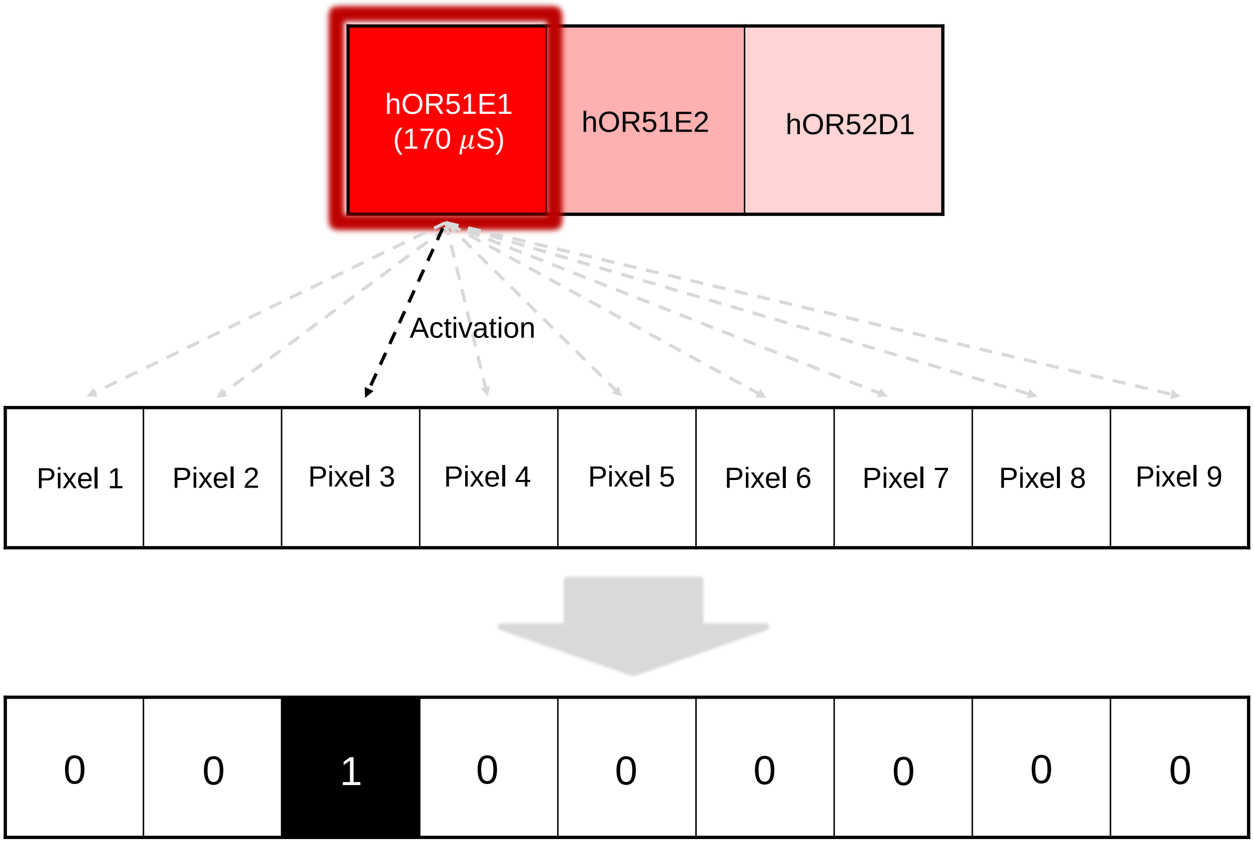

Fig. S42. Tuning process for a single hOR pixel through activation of the corresponding pixel.

Table S9. Activation conditions for each pixel of tuned pattern.

Pixel no.	Activation condition (μS)
1	$200 < G_{max}$
2	$180 < G_{max} \leq 200$
3	$160 < G_{max} \leq 180$
4	$140 < G_{max} \leq 160$
5	$120 < G_{max} \leq 140$
6	$100 < G_{max} \leq 120$
7	$80 < G_{max} \leq 100$
8	$60 < G_{max} \leq 80$
9	$G_{max} \leq 60$

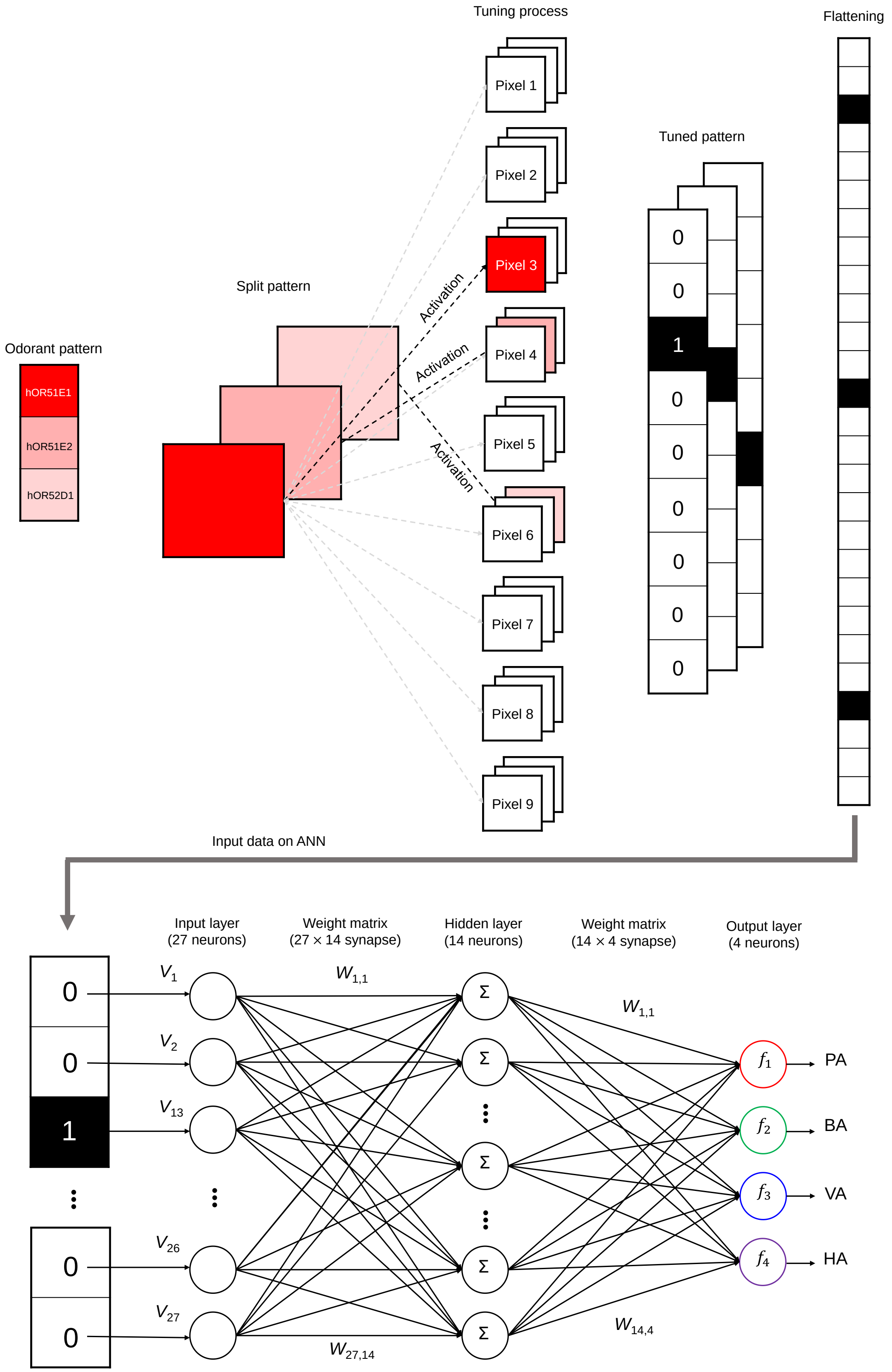

Fig. S43. Overview of data tuning process and inference simulation.

Table S10. Numbers of cases of the tuned odorant patterns 3 ppm of for PA.

Tuned pattern	Piece	Tuned pattern	Piece
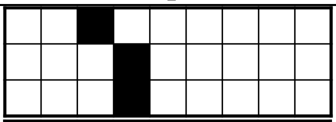	2	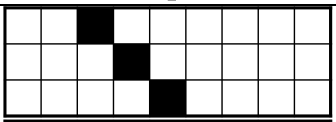	4
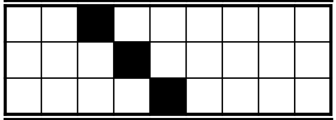	2	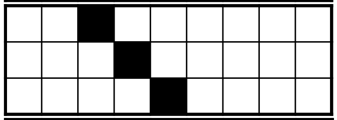	2
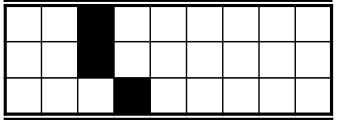	2	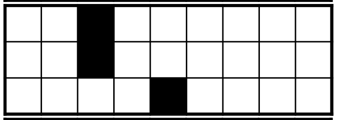	2
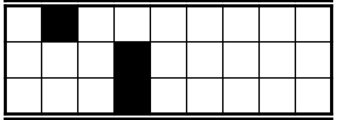	4	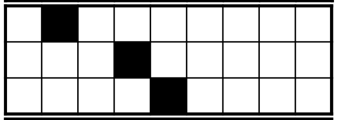	4
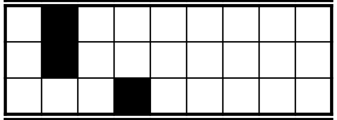	6	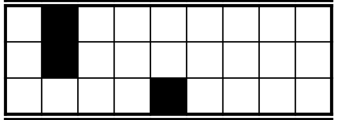	6
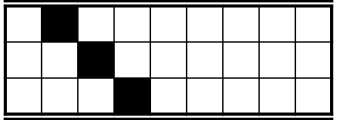	6	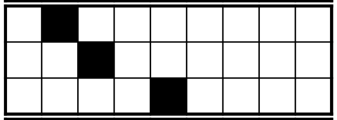	6
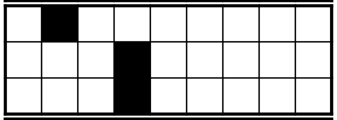	8	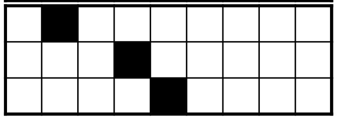	8
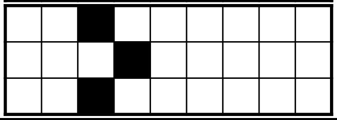	2		

Table S11. Numbers of cases of the tuned odorant patterns for 3 ppm of BA.

Tuned pattern	Piece	Tuned pattern	Piece
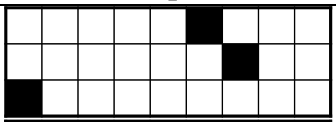	12	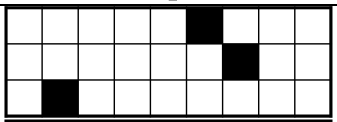	24
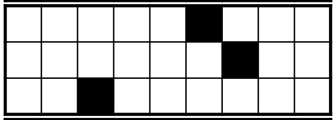	12	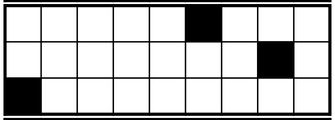	4
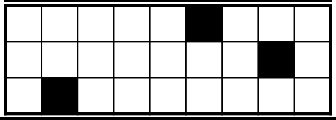	8	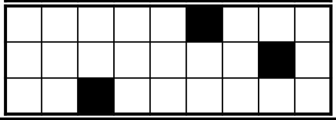	4

Table S12. Numbers of cases of the tuned odorant patterns for 3 ppm of VA.

Tuned pattern	Piece	Tuned pattern	Piece
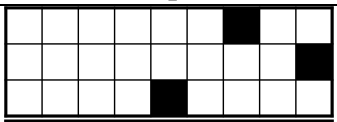	18	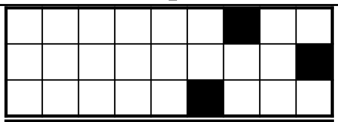	18
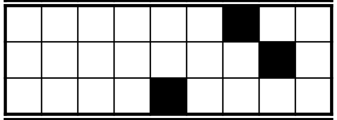	6	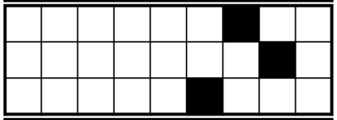	6
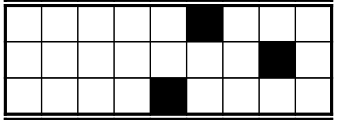	6	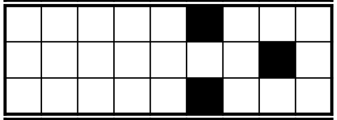	6
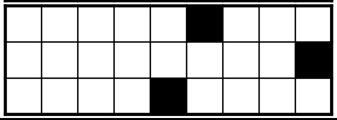	2	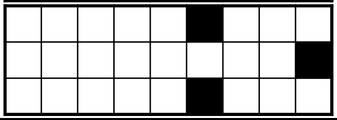	2

Table S13. Numbers of cases of the tuned odorant patterns 3 ppm of for HA.

Tuned pattern	Piece	Tuned pattern	Piece
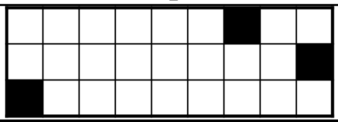	48	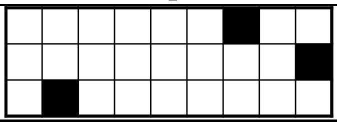	16

Table S14. Numbers of cases of the tuned odorant patterns for 1 ppm of PA.

Tuned pattern	Piece	Tuned pattern	Piece
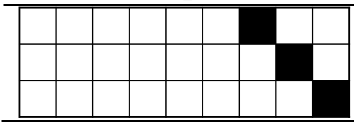	48	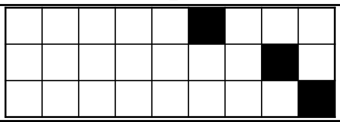	16

Table S15. Numbers of cases of the tuned odorant patterns for 1 ppm of BA.

Tuned pattern	Piece	Tuned pattern	Piece
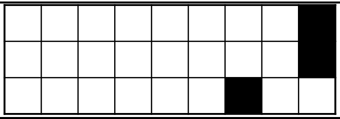	52	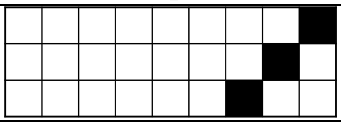	12

Table S16. Numbers of cases of the tuned odorant patterns for 1 ppm of VA.

Tuned pattern	Piece	Tuned pattern	Piece
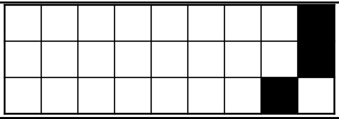	32	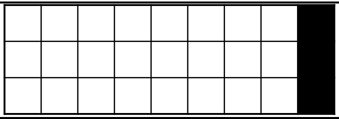	32

Table S17. Numbers of cases of the tuned odorant patterns for 1 ppm of HA.

Tuned pattern	Piece
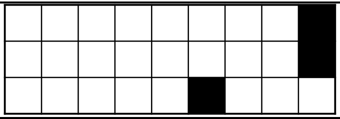	65

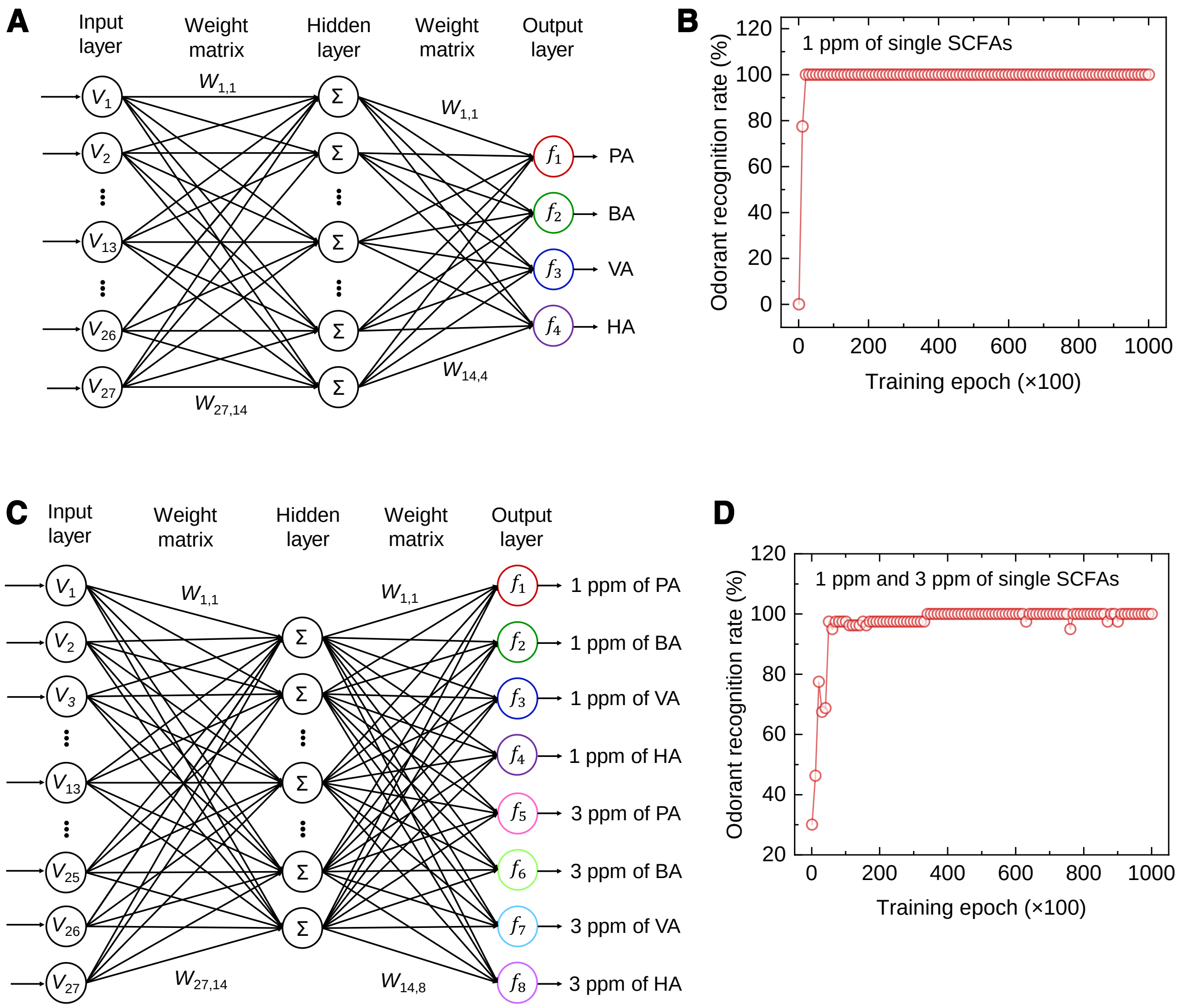

Fig. S44. Theoretical structures of artificial neural network (ANN) and odorant recognition accuracies for 1 ppm SCFAs. (A) ANN structure for learning and recognition test of 1 ppm of SCFAs dataset. **(B)** 1 ppm of single SCFAs recognition accuracy during 100,000 training and inference cycles. **(C)** ANN structure for learning and recognition test of 1 ppm and 3 ppm of SCFAs dataset. **(D)** 1 ppm and 3 ppm of single SCFAs recognition accuracy during 100,000 training and inference cycles.

Supplementary Text 7. Requirement for multi-functional AOS with three hOR NDs

We compared the odorant recognition accuracy of the AOS according to the number of hOR NDs applied. As shown in **Fig. S45 A-C**, we created original and tuned patterns for various numbers of hOR NDs. For a single hOR ND system, we designed an ANN consisting of 9 input neurons that corresponded to the 9 × 1 array pixels of the odorant images, 4 hidden neurons, 4 output neurons, and 9 × 4 × 4 artificial synapses connecting the neurons. For a double hOR ND system, the ANN comprised 18 input neurons that corresponded to the 9 × 2 array pixels of the odorant images, 9 hidden neurons, 4 output neurons, and 18 × 9 × 4 artificial synapses connecting the neurons.

Among single hOR ND systems, the AOS with hOR51E1 exhibited the highest odorant recognition accuracy (75%) (**Fig. S45D**). Among double hOR ND systems, the AOS with hOR51E2 and hOR52D1 achieved the highest odorant recognition accuracy (82.5%) (**Fig. S45E**). However, both single and double hOR ND systems had lower recognition rates compared with the AOS with triple hOR ND systems (100%) (**Fig. 45F**).

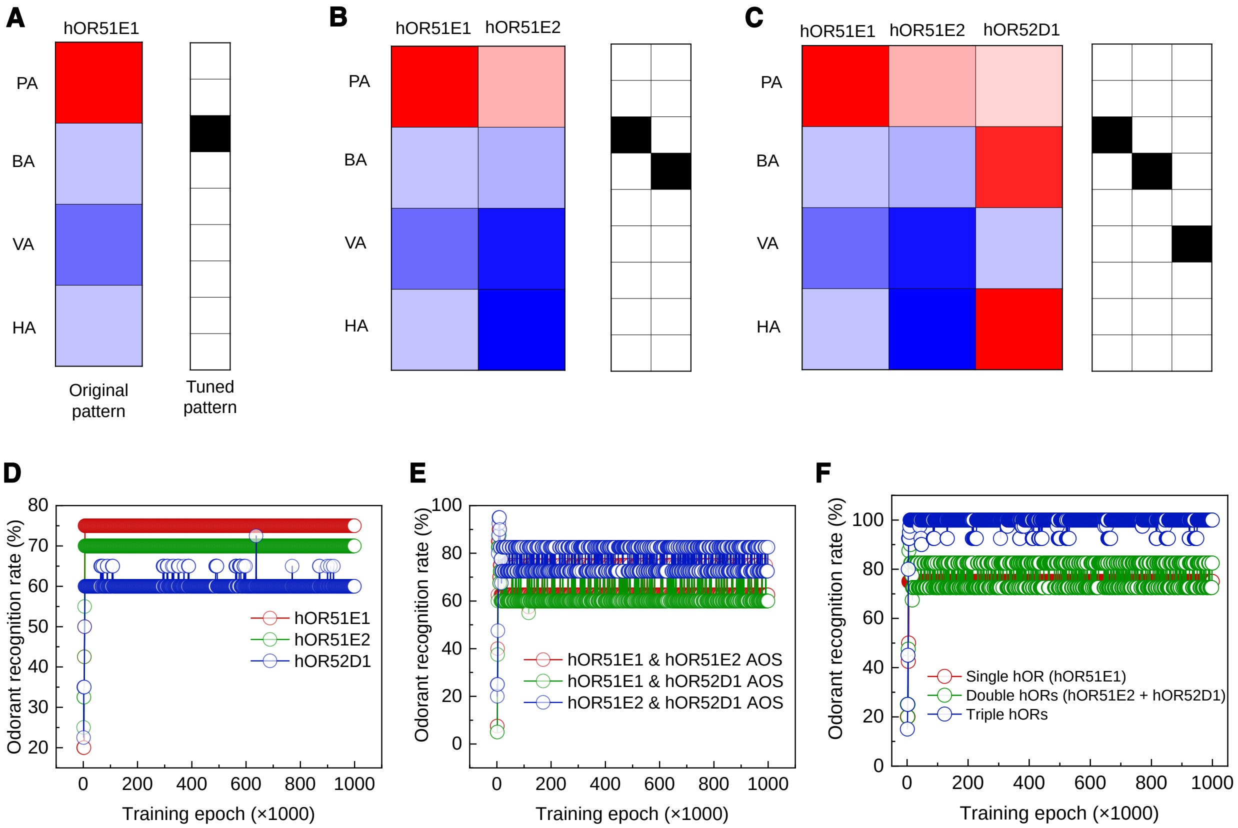

Fig. S45. Odorant recognition test for AOS according to the number of hOR NDs. (A to **C)** Original and tuned patterns of AOS with single, double, and triple hOR NDs for four different SCFAs. (A) Single hOR ND. (B) Double hOR NDs. (C) Triple hOR NDs. **(D** to **F)** Odorant recognition accuracy of AOS according to the number of hOR NDs. (D) Single hOR ND. (E) Double hOR NDs. (F) Co-plot of single, double, and triple hOR NDs.

Supplementary Text 8. Theoretical prediction of mixed-odorant patterns from experimental single odorant responses

To evaluate the odor recognition capability of our AOS for both single odorants and their mixtures, we predicted the responses of the AOS to mixtures of SCFAs using the G_{max} responses to single SCFAs obtained from actual sensing experiments. For the prediction, we assumed that each odorant signal was supposed to be 1/n of the total signal, where "n" is the number of odorants in the mixture. The final signal of the theoretical mixture was assumed to be the sum of each odorant signal. We regarded the average G_{max} responses to single SCFAs as the predicted responses to mixtures. For example, in the case of a mixture containing 1.5 ppm of PA and 1.5 ppm of BA, the G_{max} value of the hOR51E1-functionalized AOS for the mixture is 138.7 μS, which is the average value of 170.3 μS (for 3 ppm of PA) and 107.1 μS (for 3 ppm of BA). After prediction, we randomly selected 256 patterns for each odorant mixture, then categorized 2,194 patterns as training sets and 366 patterns as inference sets following the tuning process.

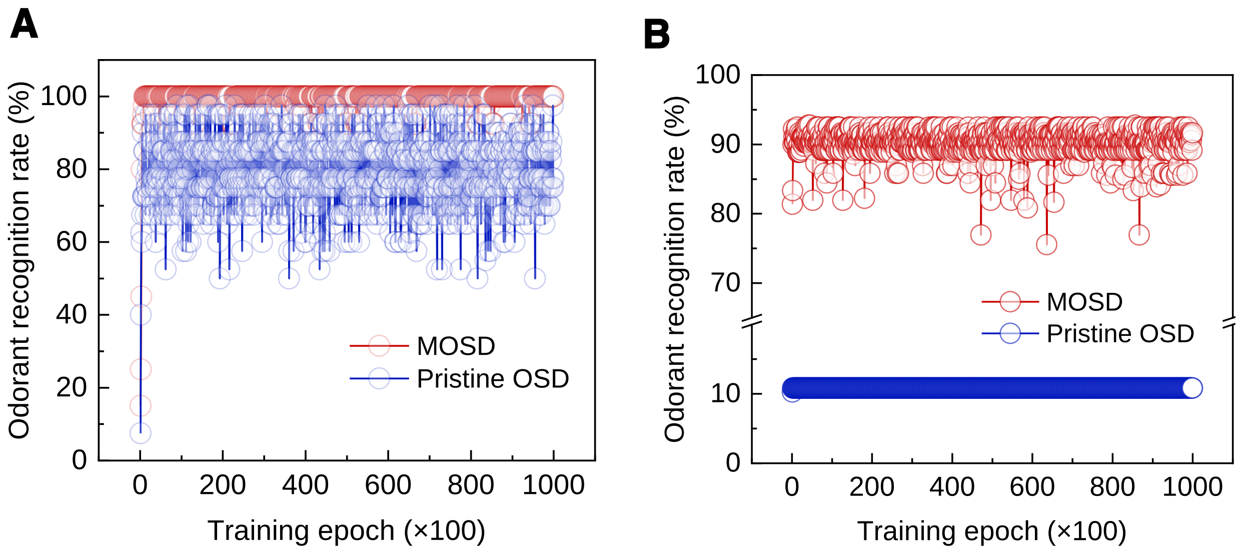

Fig. S46. Odorant recognition test for AOS comprising pristine OSD and MOSD. (A) Odorant recognition accuracies of AOSs for four different SCFAs. **(B)** Odorant recognition accuracies of AOSs for nine different mixed SCFAs patterns.

Captions for Data S1 to S4

Data S1. (separate Excel file)
Odorant recognition test dataset for 1 ppm of SCFAs

Data S2. (separate Excel file)
Odorant recognition test dataset for 3 ppm of SCFAs

Data S3. (separate Excel file)
Odorant recognition test dataset for 1 ppm and 3 ppm of SCFAs

Data S4. (separate Excel file)
Odorant recognition test dataset for 3 ppm of mixed-SCFAs

REFERENCES

1. A. Mehonic, A. J. Kenyon, Brain-inspired computing needs a master plan. *Nature* **604**, 255–260 (2022).

2. M. Wang, Y. Luo, T. Wang, C. Wan, L. Pan, S. Pan, K. He, A. Neo, X. Chen, Artificial skin perception. *Adv. Mater.* **33**, e2003014 (2021).

3. J. Yu, Y. Wang, S. Qin, G. Gao, C. Xu, Z. Lin Wang, Q. Sun, Bioinspired interactive neuromorphic devices. *Mater. Today* **60**, 158–182 (2022).

4. M. Zeng, Y. He, C. Zhang, Q. Wan, Neuromorphic devices for bionic sensing and perception. *Front. Neurosci.* **15**, 690950 (2021).

5. P. A. Merolla, A million spiking-neuron integrated circuit with a scalable communication network and interface. *Science* **345**, 668–673 (2014).

6. T. Li, H. Yu, Z. Xiong, Z. Gao, Y. Zhou, S. T. Han, 2D oriented covalent organic frameworks for alcohol-sensory synapses. *Mater. Horiz.* **8**, 2041–2049 (2021).

7. M. Bernabei, K. C. Persaud, S. Pantalei, E. Zampetti, R. Beccherelli, Large-scale chemical sensor array testing biological olfaction concepts. *IEEE Sens. J.* **12**, 3174–3183 (2012).

8. C. Qian, Y. Choi, S. Kim, S. Kim, Y. J. Choi, D. G. Roe, J. H. Lee, M. S. Kang, W. H. Lee, J. H. Cho, Risk-perceptional and feedback-controlled response system based on NO_2-detecting artificial sensory synapse. *Adv. Funct. Mater.* **32**, 2112490 (2022).

9. Y. Deng, M. Zhao, Y. Ma, S. Liu, M. Liu, B. Shen, R. Li, H. Ding, H. Cheng, X. Sheng, W. Fu, Z. Li, M. Zhang, L. Yin, A flexible and biomimetic olfactory synapse with gasotransmitter-mediated plasticity. *Adv. Funct. Mater.* **33**, 2214139 (2023).

10. H. H. Chouhdry, D. H. Lee, A. Bag, N.-E. Lee, A flexible artificial chemosensory neuronal synapse based on chemoreceptive ionogel-gated electrochemical transistor. *Nat. Commun.* **14**, 821 (2023).

11. C. Qian, Y. Choi, Y. J. Choi, S. Kim, Y. Y. Choi, D. G. Roe, M. S. Kang, J. Sun, J. H. Cho, Oxygen-detecting synaptic device for realization of artificial autonomic nervous system for maintaining oxygen homeostasis. *Adv. Mater.* **32**, e2002653 (2020).

12. T. Wang, X.-X. Wang, J. Wen, Z.-Y. Shao, H.-M. Huang, X. Guo, A bio-inspired neuromorphic sensory system. *Adv. Intell. Syst.* **4**, 2200047 (2022).

13. J. K. Han, M. Kang, J. Jeong, I. Cho, J. M. Yu, K. J. Yoon, I. Park, Y. K. Choi, Artificial olfactory neuron for an in-sensor neuromorphic nose. *Adv. Sci.* **9**, e2106017 (2022).

14. K. T. Ng, F. Boussaid, A. Bermak, A CMOS single-chip gas recognition circuit for metal oxide gas sensor arrays. *IEEE Trans. Circuits Syst.* **58**, 1569–1580 (2011).

15. D. Kwon, G. Jung, W. Shin, Y. Jeong, S. Hong, S. Oh, J.-H. Bae, B.-G. Park, J.-H. Lee, Low-power and reliable gas sensing system based on recurrent neural networks. *Sens. Actuators B Chem.* **340**, 129258 (2021).

16. D. Kwon, G. Jung, W. Shin, Y. Jeong, S. Hong, S. Oh, J. Kim, J.-H. Bae, B.-G. Park, J.-H. Lee, Efficient fusion of spiking neural networks and FET-type gas sensors for a fast and reliable artificial olfactory system. *Sens. Actuators B Chem.* **345**, 130419 (2021).

17. Y. Chu, H. Tan, C. Zhao, X. Wu, S. J. Ding, Power-efficient gas-sensing and synaptic diodes based on lateral pentacene/a-IGZO pn junctions. *ACS Appl. Mater. Interfaces* **14**, 9368–9376 (2022).

18. Z. Gao, S. Chen, R. Li, Z. Lou, W. Han, K. Jiang, F. Qu, G. Shen, An artificial olfactory system with sensing, memory and self-protection capabilities. *Nano Energy* **86**, 106078 (2021).

19. O. S. Kwon, H. S. Song, S. J. Park, S. H. Lee, J. H. An, J. W. Park, H. Yang, H. Yoon, J. Bae, T. H. Park, J. Jang, An ultrasensitive, selective, multiplexed superbioelectronic nose that mimics the human sense of smell. *Nano Lett.* **15**, 6559–6567 (2015).

20. B. Wang, J. C. Cancilla, J. S. Torrecilla, H. Haick, Artificial sensing intelligence with silicon nanowires for ultraselective detection in the gas phase. *Nano Lett.* **14**, 933–938 (2014).

21. T. Yoshizumi, T. Goda, R. Yatabe, A. Oki, A. Matsumoto, H. Oka, T. Washio, K. Toko, Y. Miyahara, Field-effect transistor array modified by a stationary phase to generate informative signal patterns for machine learning-assisted recognition of gas-phase chemicals. *Mol. Syst. Des. Eng.* **4**, 386–389 (2019).

22. C. Bur, M. Bastuck, D. Puglisi, A. Schütze, A. Lloyd Spetz, M. Andersson, Discrimination and quantification of volatile organic compounds in the ppb-range with gas sensitive SiC-FETs using multivariate statistics. *Sens. Actuators B Chem.* **214**, 225–233 (2015).

23. H. M. Fahad, H. Shiraki, M. Amani, C. Zhang, V. S. Hebbar, W. Gao, H. Ota, M. Hettick, D. Kiriya, Y.-Z. Chen, Y.-L. Chueh, A. Javey, Room temperature multiplexed gas sensing using chemical-sensitive 3.5-nm-thin silicon transistors. *Sci. Adv.* **3**, e1602557 (2017).

24. C. Bur, M. Bastuck, A. Lloyd Spetz, M. Andersson, A. Schütze, Selectivity enhancement of SiC-FET gas sensors by combining temperature and gate bias cycled operation using multivariate statistics. *Sens. Actuators B Chem.* **193**, 931–940 (2014).

25. M. Khatib, S. Rapoport, O. Zohar, E. Mansour, Y. Zheng, N. Tang, W. Saliba, Y. Mulytin, T.-P. Huynh, H. Haick, Hierarchical graphene-dye bilayers for multimodal optoelectronic sensing and decoupling of complex stimuli. *Adv. Mater. Technol.* **8**, 2200920 (2023).

26. N. Alzate-Carvajal, J. Park, I. Bargaoui, R. Rautela, Z. J. Comeau, L. Scarfe, J.-M. Ménard, S. B. Darling, B. H. Lessard, A. Luican-Mayer, Arrays of functionalized graphene chemiresistors for selective sensing of volatile organic compounds. *ACS Appl. Electron. Mater.* **5**, 1514–1520 (2023).

27. N. C. Speller, N. Siraj, B. P. Regmi, H. Marzoughi, C. Neal, I. M. Warner, Rational design of QCM-D virtual sensor arrays based on film thickness, viscoelasticity, and harmonics for vapor discrimination. *Anal. Chem.* **87**, 5156–5166 (2015).

28. K. Minami, K. Shiba, G. Yoshikawa, Discrimination of structurally similar odorous molecules with various concentrations by using a nanomechanical sensor. *Anal. Methods* **10**, 3720–3726 (2018).

29. K. Yang, C. Zhang, K. Zhu, Z. Qian, Z. Yang, L. Wu, S. Zong, Y. Cui, Z. Wang, A programmable plasmonic gas microsystem for detecting arbitrarily combinated volatile organic compounds (VOCs) with ultrahigh resolution. *ACS Nano* **16**, 19335–19345 (2022).

30. G. Piszter, K. Kertész, Z. Vértesy, Z. Bálint, L. P. Biró, Vapor sensing of pristine and ALD modified butterfly wings. *Mater. Today Proc.* **1**, 216–220 (2014).

31. Y. Hu, Z. Zhou, F. Zhao, X. Liu, Y. Gong, W. Xiong, M. Sillanpää, Fingerprint detection and differentiation of gas-phase amines using a fluorescent sensor array assembled from asymmetric perylene diimides. *Sci. Rep.* **8**, 10277 (2018).

32. R. A. Potyrailo, M. Larsen, O. Riccobono, Detection of individual vapors and their mixtures using a selectivity-tunable three-dimensional network of plasmonic nanoparticles. *Angew. Chem. Int. Ed.* **52**, 10360–10364 (2013).

33. R. A. Potyrailo, R. K. Bonam, J. G. Hartley, T. A. Starkey, P. Vukusic, M. Vasudev, T. Bunning, R. R. Naik, Z. Tang, M. A. Palacios, M. Larsen, L. A. Le Tarte, J. C. Grande, S. Zhong, T. Deng, Towards outperforming conventional sensor arrays with fabricated individual photonic vapour sensors inspired by Morpho butterflies. *Nat. Commun.* **6**, 7959 (2015).

34. R. A. Potyrailo, Z. Ding, M. D. Butts, S. E. Genovese, T. Deng, Selective chemical sensing using structurally colored core-shell colloidal crystal films. *IEEE Sens. J.* **8**, 815–822 (2008).

35. R. A. Potyrailo, H. Ghiradella, A. Vertiatchikh, K. Dovidenko, J. R. Cournoyer, E. Olson, Morpho butterfly wing scales demonstrate highly selective vapour response. *Nat. Photonics* **1**, 123–128 (2007).

36. N. A. Joy, M. I. Nandasiri, P. H. Rogers, W. Jiang, T. Varga, S. V. N. T. Kuchibhatla, S. Thevuthasan, M. A. Carpenter, Selective plasmonic gas sensing: H_2, NO_2, and CO spectral discrimination by a single Au-CeO_2 nanocomposite film. *Anal. Chem.* **84**, 5025–5034 (2012).

37. S. Firestein, How the olfactory system makes sense of scents. *Nature* **413**, 211–218 (2001).

38. I. Gaillard, S. Rouquier, D. Giorgi, Olfactory receptors. *Cell Mol.Life Sci.* **61**, 456–469 (2004).

39. U. B. Kaupp, Olfactory signalling in vertebrates and insects: Differences and commonalities. *Nat. Rev. Neurosci.* **11**, 188–200 (2010).

40. C.-Y. Su, K. Menuz, J. R. Carlson, Olfactory perception: Receptors, cells, and circuits. *Cell* **139**, 45–59 (2009).

41. E. A. Hallem, J. R. Carlson, Coding of odors by a receptor repertoire. *Cell* **125**, 143–160 (2006).

42. Y. van de Burgt, A. Melianas, S. T. Keene, G. Malliaras, A. Salleo, Organic electronics for neuromorphic computing. *Nat. Electron.* **1**, 386–397 (2018).

43. K. H. Kim, D. Moon, J. E. An, S. J. Park, S. E. Seo, S. Ha, J. Kim, K. Kim, S. Phyo, J. Lee, H. Y. Kim, M. Kim, T. H. Park, H. S. Song, O. S. Kwon, Wireless portable bioelectronic nose device for multiplex monitoring toward food freshness/spoilage. *Biosens. Bioelectron.* **215**, 114551 (2022).

44. I. G. Denisov, S. G. Sligar, Nanodiscs in membrane biochemistry and biophysics. *Chem. Rev.* **117**, 4669–4713 (2017).

45. I. G. Denisov, S. G. Sligar, Nanodiscs for structural and functional studies of membrane proteins. *Nat. Struct. Mol. Biol.* **23**, 481–486 (2016).

46. H. Yang, D. Kim, J. Kim, D. Moon, H. S. Song, M. Lee, S. Hong, T. H. Park, Nanodisc-based bioelectronic nose using olfactory receptor produced in *Escherichia coli* for the assessment of the death-associated odor cadaverine. *ACS Nano* **11**, 11847–11855 (2017).

47. J. Oh, H. Yang, G. E. Jeong, D. Moon, O. S. Kwon, S. Phyo, J. Lee, H. S. Song, T. H. Park, J. Jang, Ultrasensitive, selective, and highly stable bioelectronic nose that detects the liquid and gaseous cadaverine. *Anal. Chem.* **91**, 12181–12190 (2019).

48. M. Lee, H. Yang, D. Kim, M. Yang, T. H. Park, S. Hong, Human-like smelling of a rose scent using an olfactory receptor nanodisc-based bioelectronic nose. *Sci. Rep.* **8**, 13945 (2018).

49. L. F. R. Abbott, W. G., Synaptic computation. *Nature* **431**, 796–803 (2004).

50. J. D. Mainland, A. Keller, Y. R. Li, T. Zhou, C. Trimmer, L. L. Snyder, A. H. Moberly, K. A. Adipietro, W. L. Liu, H. Zhuang, S. Zhan, S. S. Lee, A. Lin, H. Matsunami, The missense of smell: Functional variability in the human odorant receptor repertoire. *Nat. Neurosci.* **17**, 114–120 (2014).

51. N. Jovancevic, A. Dendorfer, M. Matzkies, M. Kovarova, J. C. Heckmann, M. Osterloh, M. Boehm, L. Weber, F. Nguemo, J. Semmler, J. Hescheler, H. Milting, E. Schleicher, L. Gelis, H. Hatt, Medium-chain fatty acids modulate myocardial function via a cardiac odorant receptor. *Basic Res. Cardiol.* **112**, 13 (2017).

52. C. A. de March, S. Ryu, G. Sicard, C. Moon, J. Golebiowski, Structure-odour relationships reviewed in the postgenomic era. *Flavour Fragr. J.* **30**, 342–361 (2015).

53. T. H. Bayburt, Y. V. Grinkova, S. G. Sligar, Self-assembly of discoidal phospholipid bilayer nanoparticles with membrane scaffold proteins. *Nano Lett.* **2**, 853–856 (2002).

54. I. G. Denisov, Y. V. Grinkova, A. A. Lazarides, S. G. Sligar, Directed self-assembly of monodisperse phospholipid bilayer nanodiscs with controlled size. *J. Am. Chem. Soc.* **126**, 3477–3487 (2004).

55. F. Hagn, M. L. Nasr, G. Wagner, Assembly of phospholipid nanodiscs of controlled size for structural studies of membrane proteins by NMR. *Nat. Protoc.* **13**, 79–98 (2018).

56. X. Ji, B. D. Paulsen, G. K. K. Chik, R. Wu, Y. Yin, P. K. L. Chan, J. Rivnay, Mimicking associative learning using an ion-trapping non-volatile synaptic organic electrochemical transistor. *Nat. Commun.* **12**, 2480 (2021).

57. Y. van de Burgt, E. Lubberman, E. J. Fuller, S. T. Keene, G. C. Faria, S. Agarwal, M. J. Marinella, A. Alec Talin, A. Salleo, A non-volatile organic electrochemical device as a low-voltage artificial synapse for neuromorphic computing. *Nat. Mater.* **16**, 414–418 (2017).

58. J. Rivnay, S. Inal, A. Salleo, R. M. Owens, M. Berggren, G. G. Malliaras, Organic electrochemical transistors. *Nat. Rev. Mater.* **3**, 17086 (2018).

59. L. Yang, L. Zhou, Pei Yuan, Jin Zou, α-MoO_3 nanobelts: A high performance cathode material for lithium ion batteries. *J. Phys. Chem. C* **114**, 21868–21872 (2010).

60. H. S. Kim, J. B. Cook, H. Lin, J. S. Ko, S. H. Tolbert, V. Ozolins, B. Dunn, Oxygen vacancies enhance pseudocapacitive charge storage properties of MoO_{3-x}. *Nat. Mater.* **16**, 454–460 (2017).

61. S. E. Ng, Y. B. Tay, T. Y. K. Ho, Ankit, N. Mathews, Inorganic electrochromic transistors as environmentally adaptable photodetectors. *Nano Energy* **97**, 107142 (2022).

62. Y. Yamashita, J. Tsurumi, M. Ohno, R. Fujimoto, S. Kumagai, T. Kurosawa, T. Okamoto, J. Takeya, S. Watanabe, Efficient molecular doping of polymeric semiconductors driven by anion exchange. *Nature* **572**, 634–638 (2019).

63. M. E. Adam, M. Fehervari, P. R. Boshier, S. T. Chin, G. P. Lin, A. Romano, S. Kumar, G. B. Hanna, Mass-spectrometry analysis of mixed-breath, isolated-bronchial-breath, and gastric-endoluminal-air volatile fatty acids in esophagogastric cancer. *Anal. Chem.* **91**, 3740–3746 (2019).

64. S. Kumar, J. Huang, J. R. Cushnir, P. Spanel, D. Smith, G. B. Hanna, Selected ion flow tube-MS analysis of headspace vapor from gastric content for the diagnosis of gastro-esophageal cancer. *Anal. Chem.* **84**, 9550–9557 (2012).

65. S. Kumar, J. Huang, N. Abbassi-Ghadi, H. A. Mackenzie, K. A. Veselkov, J. M. Hoare, L. B. Lovat, P. Spanel, D. Smith, G. B. Hanna, Mass spectrometric analysis of exhaled breath for the identification of volatile organic compound biomarkers in esophageal and gastric adenocarcinoma. *Ann. Surg.* **262**, 981–990 (2015).

66. S. R. Markar, T. Wiggins, S. Antonowicz, S. T. Chin, A. Romano, K. Nikolic, B. Evans, D. Cunningham, M. Mughal, J. Lagergren, G. B. Hanna, Assessment of a noninvasive exhaled breath test for the diagnosis of oesophagogastric cancer. *JAMA Oncol.* **4**, 970–976 (2018).

67. J. Huang, S. Kumar, N. Abbassi-Ghadi, P. Spanel, D. Smith, G. B. Hanna, Selected ion flow tube mass spectrometry analysis of volatile metabolites in urine headspace for the profiling of gastro-esophageal cancer. *Anal. Chem.* **85**, 3409–3416 (2013).

68. R. Niedermanh, Y. Buyle-Bodin, B.-Y. Lu, P. Robinson, C. Naleway, Short-chain carboxylic acid concentration in human gingival crevicular fluid. *J. Dent. Res.* **76**, 575–579 (1997).

69. R. M. Thorn, J. Greenman, Microbial volatile compounds in health and disease conditions. *J. Breath Res.* **6**, 024001 (2012).

70. B. U. Aylikci, H. Colak, Halitosis: From diagnosis to management. *J. Nat. Sci. Biol. Med.* **4**, 14–23 (2013).

71. C. S. Yang, D. S. Shang, N. Liu, E. J. Fuller, S. Agrawal, A. A. Talin, Y. Q. Li, B. G. Shen, Y. Sun, All-solid-state synaptic transistor with ultralow conductance for neuromorphic computing. *Adv. Funct. Mater.* **28**, 1804170 (2018).

72. C. S. Yang, D. S. Shang, N. Liu, G. Shi, X. Shen, R. C. Yu, Y. Q. Li, Y. Sun, A synaptic transistor based on quasi-2D molybdenum oxide. *Adv. Mater.* **29**, 1700906 (2017).

73. I. A. de Castro, R. S. Datta, J. Z. Ou, A. Castellanos-Gomez, S. Sriram, T. Daeneke, K. Kalantar-Zadeh, Molybdenum oxides–From fundamentals to functionality. *Adv. Mater.* **29**, 1701619 (2017).

74. M. F. L Seguin, R Cavagnat, J.-C Lassègues, Infrared and raman spectra of MoO_3 molybdenum trioxides and $MoO_3 \cdot xH_2O$ molybdenum trioxide hydrates. *Spectrochim. Acta A Mol. Biomol. Spectrosc.* **51**, 1323–1344 (1995).

75. C. B. Billesbølle, C. A. de March, W. J. C. van der Velden, N. Ma, J. Tewari, C. L. del Torrent, L. Li, B. Faust, N. Vaidehi, H. Matsunami, A. Manglik, Structural basis of odorant recognition by a human odorant receptor. *Nature* **615**, 742–749 (2023).

76. C. Choi, J. Bae, S. Kim, S. Lee, H. Kang, J. Kim, I. Bang, K. Kim, W.-K. Huh, C. Seok, H. Park, W. Im, H.-J. Choi, Understanding the molecular mechanisms of odorant binding and activation of the human OR52 family. *Nat. Commun.* **14**, 8105 (2023).

77. A. Mafi, S.-K. Kim, W. A. Goddard, The mechanism for ligand activation of the GPCR–G protein complex. *Proc. Natl. Acad. Sci. U.S.A.* **119**, e2110085119 (2022).

78. A. Lagunas, C. Belloir, L. Briand, P. Gorostiza, J. Samitier, Determination of the nanoscale electrical properties of olfactory receptor hOR1A1 and their dependence on ligand binding: Towards the development of capacitance-operated odorant biosensors. *Biosens. Bioelectron.* **218**, 114755 (2022).

79. D. Bajusz, A. Rácz, K. Héberger, Why is Tanimoto index an appropriate choice for fingerprint-based similarity calculations? *J. Chem.* **7**, 20 (2015).
